# *Listeria* promotes systemic infection by targeting the host deubiquitinase CYLD

**DOI:** 10.1038/s41467-026-77063-5

**Published:** 2026-08-31

**Authors:** Dustin A. Ammendolia, Cara J. Ellison, Yuelin Zheng, Etienne Coyaud, Estelle M. N. Laurent, Bing-Ru Yan, Bri Manning, Anna K. Waldmann, Joel M. J. Tan, Scott Frendo-Cumbo, Carina Lyons, Ji-Young Youn, Brian Raught, Keith Ireton, Darren E. Higgins, Paul R. Elliott, John H. Brumell

**Affiliations:** 1 Cell & Systems Biology Program, Hospital for Sick Children, Toronto, ON Canada; 2 Department of Molecular Genetics, University of Toronto, Toronto, ON Canada; 3 Department of Biochemistry, University of Oxford, Oxford, UK; 4 Princess Margaret Cancer Centre, University Health Network, Toronto, ON Canada; 5 Department of Microbiology, Blavatnik Institute, Harvard Medical School, Boston, MA USA; 6 Institute of Medical Science, University of Toronto, Toronto, ON Canada; 7 Department of Physiology, University of Toronto, Toronto, ON Canada; 8 Molecular Medicine Program, Hospital for Sick Children, Toronto, ON Canada; 9 Department of Medical Biophysics, University of Toronto, Toronto, ON Canada; 10 Department of Microbiology and Immunology, University of Otago, Dunedin, New Zealand; 11 SickKids IBD Centre, Hospital for Sick Children, Toronto, ON Canada

**Keywords:** Bacterial pathogenesis, Pathogens, Bacterial infection, Cellular microbiology

## Abstract

*Listeria monocytogenes* (*Lm*) is an intracellular pathogen that can cause life-threatening systemic infections. Internalin C (InlC) is a secreted factor required for full *Lm* virulence in systemic models of infection by mechanisms that remain unclear. Here, we show that InlC binds to CYLD, a host deubiquitinase and regulator of innate immunity. Structurally, we reveal the LRR domain of InlC binds the CAP-Gly2 domain of CYLD, independently of InlC’s binding site for other host target proteins. *Lm* strains harboring amino acid substitutions in InlC that selectively disrupt binding to CYLD were examined. We demonstrate that after *Lm* accesses the host cytosol, InlC recruits CYLD to ubiquitin-positive bacteria. Recruitment of CYLD was dependent on the host E3 ligase RNF213, a major initiator of ubiquitin-mediated defenses. Furthermore, InlC-CYLD binding contributed to *Lm* virulence in mice. Together, these findings reveal how a secreted bacterial factor promotes the recruitment of a host deubiquitinase to cytosolic bacteria in response to ubiquitin-mediated defenses.

## Abstract (web summary)

Secretion of protein InlC into the host cytosol is important for virulence of the intracellular pathogen *Listeria monocytogenes*. Here, Ammendolia et al. show that InlC recruits a host deubiquitinase to cytosolic bacteria in response to ubiquitin-mediated defenses.

## Introduction

Ubiquitin-mediated defenses are critical for the detection and control of intracellular pathogens, leading to outcomes such as immune signaling and autophagic clearance^1^. The host E3 ligase RNF213 is a key initiator of this process, capable of ubiquitinating a diverse set of pathogens from bacteria to parasites^2^. Bacterial countermeasures against RNF213 activity are best understood in the context of gram-negative bacteria upon ubiquitination of lipopolysaccharide^3–5^, whereas little is known about the response of gram-positive species.

*Listeria monocytogenes* (*Lm*) is a gram-positive, facultative intracellular bacterium that causes listeriosis, a systemic form of disease with a high mortality rate (20%–30%) amongst foodborne pathogens^6^. The ability of *Lm* to grow within the cytosol of host cells and to spread directly from cell to cell contributes to its virulence^7^. After uptake by host cells, *Lm* employs a pore-forming toxin and phospholipase C enzymes to facilitate vacuolar escape. Upon entering the cytosol, *Lm* undergoes actin-based motility through the expression of ActA, a bacterial surface protein^8^. Through binding to host actin regulatory factors, ActA promotes intracellular motility in the cytosol and the formation of cell surface protrusions, leading to eventual uptake by adjacent cells. ActA is also known to have other functions^9^, including evasion of host ubiquitin-mediated defenses^2,10,11^. Indeed, RNF213 has been shown to promote ubiquitination events at the surface of *Lm* that are greatly enhanced in non-motile (Δ*actA*) bacteria^2,11^. Whether additional virulence factors act cooperatively with ActA to mediate evasion of ubiquitin-mediated defenses remains unclear.

Internalin C (InlC) is a secreted *Lm* virulence factor that is highly expressed when bacteria access the cytosol of host cells^12–14^. While InlC is not required for *Lm* growth in the cytosol of various cell lines in vitro^12^, it has been linked to cellular phenotypes relevant to in vivo infection. Specifically, InlC binds the host actin regulator TUBA to promote cell-to-cell spread^14,15^ and also interacts with the IκB kinase (IKK) complex to dampen innate immune signaling^16^. These versatile roles led us to speculate that InlC has additional binding partners in the host cell cytosol that enable *Lm* to cause systemic infection.

Here, we identify the host deubiquitinase CYLD as a target of InlC. We reveal that InlC binds directly to the CAP-Gly2 domain of CYLD, independently of its known protein-protein interactions with other host binding partners. InlC binding does not impair CYLD’s deubiquitinase activity, but instead promotes CYLD recruitment to ubiquitinated bacteria in a manner dependent on RNF213 activity. We further demonstrate that InlC-CYLD binding contributes to *Lm* virulence in vivo by promoting bacterial dissemination in the liver. Collectively, our results reveal how a secreted bacterial factor promotes the recruitment of a host deubiquitinase in response to host ubiquitin defenses.

## Results

### InlC binds the host deubiquitinase CYLD

**Fig. 1:**
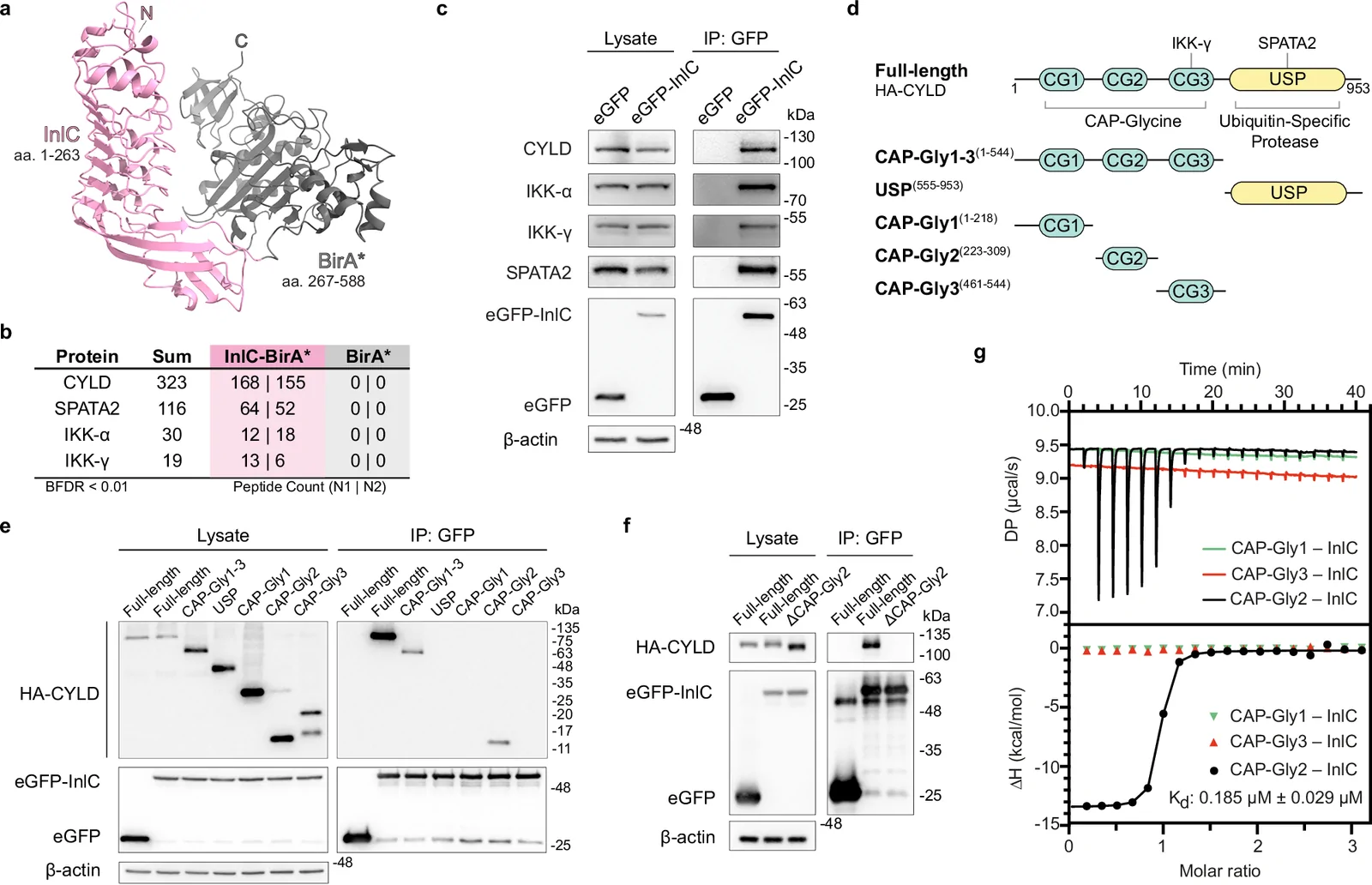
InlC binds the host deubiquitinase CYLD. **a** AlphaFold2-generated structure of the secreted form of InlC (aa. 35–297) fused to BirA* for BioID in HEK293 Flp-In T-REx cells. **b** Identity and sum of peptide counts of potential interactors detected by InlC-BirA*. Peptide counts of *N* = *2* biological replicates, each comprised of two technical replicates, are displayed with a Bayesian false discovery rate (BFDR) < 0.01. All proteins detected are included in Supplementary Data 1. **c** Immunoprecipitation of transfected eGFP-InlC in HEK293T cells. Immunoblot is representative of *N* = 3 biological replicates. **d** Schematic of HA-CYLD domains and the binding sites of previously identified interactors. **e**, **f** Co-immunoprecipitation of eGFP-InlC and HA-CYLD domain truncations in *CYLD* KO HEK293T cells. Immunoblots are representative of *N* = 3 biological replicates. **g** ITC thermograms of recombinant InlC (aa. 35–297) incubated with CYLD CAP-Gly1, CAP-Gly2, or CAP-Gly3. Raw isotherms are shown (top) with integrated fits (bottom) for InlC and CAP-Gly2. Data shown are representative of one (CAP-Gly1, CAP-Gly3) or three (CAP-Gly2) technical replicates. Source data are provided as a Source Data file.

To identify host protein interactors of InlC, we performed proximity-dependent biotin identification (BioID)^17,18^ in HEK293 cells by expressing the secreted portion of InlC fused to an abortive mutant of the *Escherichia coli*-derived biotin ligase BirA (R118G, termed BirA*)^19^ (Fig. 1a and Supplementary Fig. 1a, b). Relative to the BirA* control, we identified CYLD and its binding partner SPATA2 as high-confidence proximity interactors of InlC (Fig. 1b and Supplementary Data 1). A previous high-throughput screen identified CYLD as a potential interactor of InlC^20^. Two members of the IκB kinase (IKK) complex, IKK-α and IKK-γ, were also detected, consistent with prior observations^21^. Through co-immunoprecipitation using eGFP-InlC as bait, we validated the binding of InlC to CYLD, SPATA2, and IKK-α/-γ (Fig. 1c). As it remains unknown how *Lm* counteracts host ubiquitin defenses, we sought to further characterize the interaction of InlC with CYLD.

CYLD consists of three cytoskeleton-associated protein glycine-rich protein (CAP-Gly) domains and a C-terminal ubiquitin-specific peptidase (USP) domain (Fig. 1d). Established protein-protein interactions for these domains include IKK-γ (CAP-Gly3) and SPATA2 (USP) (Fig. 1d)^22,23^. To determine which region of CYLD mediates binding to InlC, we performed co-immunoprecipitation of eGFP-InlC in *CYLD* KO HEK293T cells complemented with individual domains of HA-CYLD (Fig. 1d, e). We observed that eGFP-InlC bound the N-terminal half of HA-CYLD (CAP-Gly1-3). Binding to the CAP-Gly2 domain was deemed both sufficient (Fig. 1e) and necessary (Fig. 1f) for binding to InlC.

As a complementary approach, we used isothermal titration calorimetry (ITC) to measure direct binding between recombinant InlC and CAP-Gly2 (Fig. 1g). InlC bound with high affinity to the CAP-Gly2 domain of CYLD with a Kd of 185 ± 29.5 nM. In contrast, InlC did not bind to either the CAP-Gly1 or CAP-Gly3 domains. These data demonstrate that InlC directly binds to CYLD via its CAP-Gly2 domain.

### Structural analysis of the InlC-CYLD binding interface

**Fig. 2:**
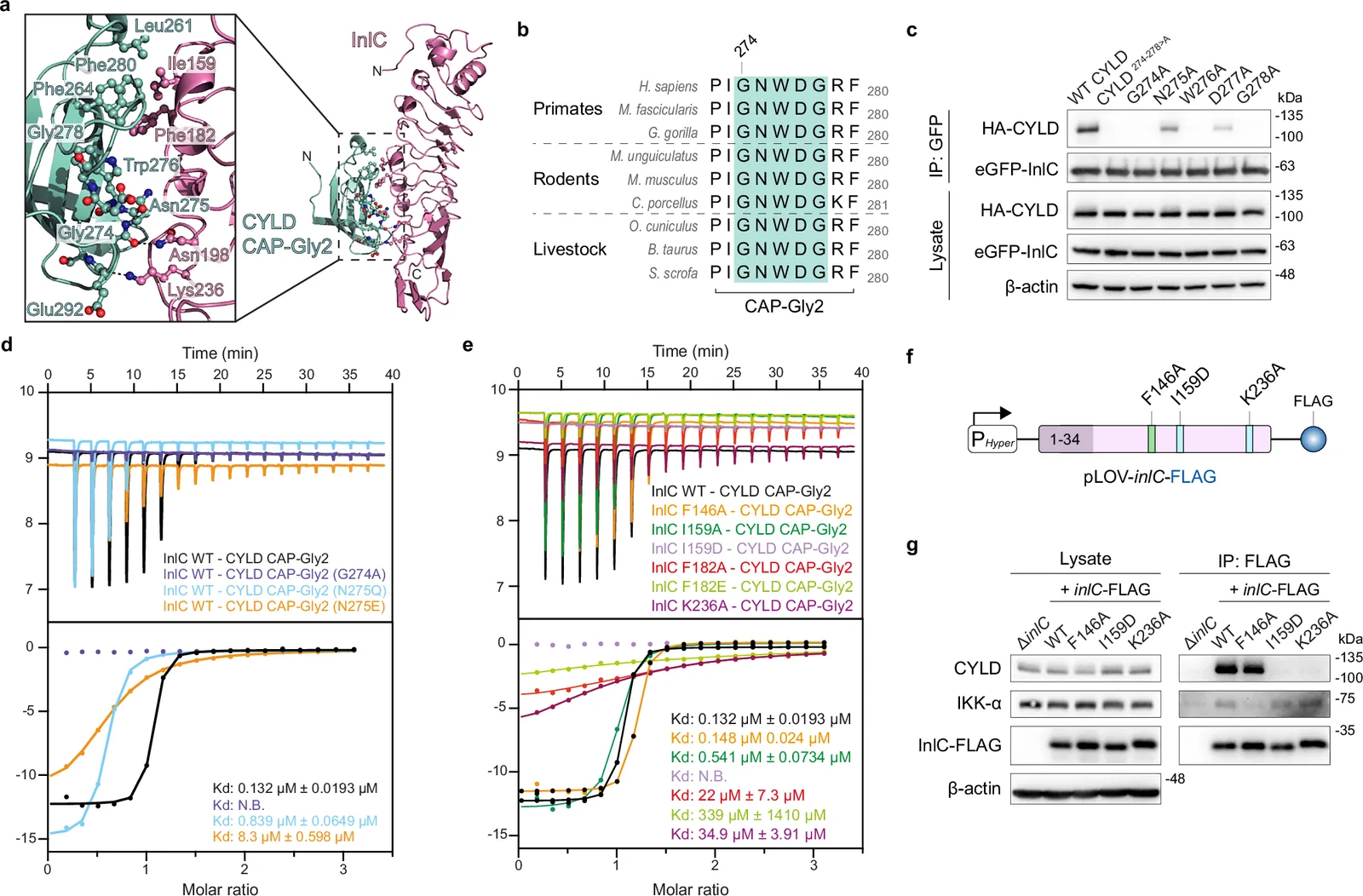
Structural analysis of the InlC-CYLD binding interface. **a** Crystal structure of InlC in complex with CAP-Gly2 of CYLD (PDB: 9TMK). **b** Sequence alignment of conserved CAP-Gly2 residues at the binding interface. **c**, Co-immunoprecipitation of eGFP-InlC and HA-CYLD harboring mutations in the CAP-Gly2 domain in *CYLD* KO HEK293T cells. Immunoblot is representative of *N* = 3 biological replicates. **d** ITC thermograms of recombinant InlC (aa. 35–297) incubated with CAP-Gly2 harboring single mutations at the binding interface. Raw isotherms are shown (top) with integrated fits for all samples except CAP-Gly2 G274A (bottom). Data shown are representative of one (CAP-Gly2 mutants) or two (WT CAP-Gly2) technical replicates. **e** ITC thermograms of recombinant CAP-Gly2 incubated with InlC (aa. 35–297) harboring single mutations at the binding interface. Raw isotherms are shown (top) with integrated fits for all samples except InlC I159D (bottom). Data shown are representative of one (InlC mutants) or two (WT InlC) technical replicates. **f** Schematic of pLOV-*inlC*-FLAG used for complementation of Δ*inlC Lm* with either full-length wildtype *inlC* (aa. 1–297) or single point mutants defective in binding to TUBA and IKK-α (F146A) or CYLD (I159D, K236A). **g** Immunoprecipitation of InlC-FLAG from *Lm*-infected mouse bone marrow-derived macrophages (6.5 h post-infection). Immunoblot is representative of *N* = 3 biological replicates. Source data are provided as a Source Data file.

The secreted form of InlC comprises an α-helical-cap, a leucine-rich repeat (LRR) domain, and an IgG-like domain^24^. To define the molecular interaction between InlC and CYLD CAP-Gly2, we determined the structure of the complex to 1.8 Å resolution by X-ray crystallography (Fig. 2a, Supplementary Fig. 2, and Supplementary Table 1). Dissection of this interaction highlighted two key interfaces employing different modes of action. First, a hydrophobic interface clustered around InlC residues Ile159 and Phe182 stabilizes a hydrophobic patch in CYLD including residues Leu261, Phe264 and Phe280. The second interface is driven by hydrogen bond interactions from InlC residues Asn198 and Lys236 to CYLD backbone carbonyl groups of Gly274 and Glu292, respectively (Fig. 2a).

Notably, Gly274 lies within the “GKNDG” motif signature of CAP-Gly domains^25^. Sequence alignment revealed that this motif is highly conserved amongst species where listeriosis has been reported^6^ (Fig. 2b), suggesting an important role in pathogenesis. Mutagenesis of the motif in its entirety or single residues (G274A, W276A, G278A) was found to block or greatly reduce CYLD binding to InlC as determined by co-immunoprecipitation (Fig. 2c). ITC analysis additionally confirmed that mutation of G274A prevented recombinant InlC binding, whereas mutation of CYLD Asn275 to introduce steric hinderance (N275Q) or a repulsive charge (N275E) only reduced the affinity for InlC (Fig. 2d).

The CAP-Gly2 domain can bind ubiquitin to regulate the deubiquitinase activity of CYLD^26^. However, mutation of Phe288 (F288A) within CAP-Gly2, a critical residue for binding ubiquitin, did not impact InlC binding to CYLD as determined by co-immunoprecipitation (Supplementary Fig. 3a, b). Furthermore, using a cell-free system with full-length recombinant CYLD, we did not observe any impact of InlC on CYLD deubiquitinase activity against either Lys63- or Met1-linkages (Supplementary Fig. 3c). Thus, InlC binds the CAP-Gly2 domain of CYLD, independently of CAP-Gly2-ubiquitin binding, and does not impact the deubiquitinase activity of CYLD.

Next, we examined critical residues of InlC that mediate binding to the CAP-Gly2 domain of CYLD (Fig. 2e). Mutation of bulky hydrophobic residues I159A or F182A within InlC reduced binding affinity to CAP-Gly2 (4-fold and 166-fold, respectively), whereas introduction of a negative charge abolished (I159D) or severely reduced (F182E, 2568-fold) binding. Also of note, the disruption of hydrogen bonds from InlC Lys236 reduced binding affinity to CAP-Gly2 by 264-fold. Previous work revealed that host-mediated ubiquitination of InlC impacts its interaction with host proteins during *Lm* infection^20^. This led us to question whether modification of other InlC lysine residues, potential targets of ubiquitination^20^, influenced binding to CYLD in a cellular context. However, mutagenesis of all other lysine residues of InlC did not affect CYLD binding (Supplementary Fig. 3d, e). Furthermore, using recombinant proteins, a GST pulldown assay confirmed that InlC K236A blocked binding to CAP-Gly2 (Supplementary Fig. 3f). We conclude that Ile159 and Lys236 of InlC play critical roles in binding to the CAP-Gly2 domain of CYLD.

### The LRR domain of InlC has two binding sites for its host protein targets

During the cytosolic phase of *Lm* infection, secreted InlC binds the cortical actin regulator TUBA to promote *Lm* protrusion formation^14,15^. The LRR domain of InlC mediates binding to the sixth Src Homology 3 domain (SH3-6) present in TUBA, and InlC Phe146 was identified as a critical residue at this binding interface^14^. Overlay of the InlC-CAP-Gly2 structure with a previously determined structure of InlC bound to the SH3-6 domain of TUBA (PDB: 4CC4) revealed independent binding sites on opposing faces of InlC (Supplementary Fig. 4a). This finding was experimentally validated through ITC as both WT and K236A InlC bound the purified SH3-6 domain of TUBA (Supplementary Fig. 4b). In contrast, mutation of InlC Phe146 to alanine (F146A) abrogated binding to TUBA, consistent with prior observations^14^ (Supplementary Fig. 4b).

InlC can also bind IKK-α, and this interaction is suggested to dampen immune signaling during infection^21^. Using a co-immunoprecipitation assay with eGFP-InlC as bait, we found that the InlC F146A mutation also blocked binding to IKK-α/-β/-γ (Supplementary Fig. 4c). In contrast, InlC mutants that perturb CYLD binding (I159D and K236A) did not impact the interaction of InlC with the IKK complex. We conclude that the LRR domain of InlC employs two different binding sites for its host protein targets.

### InlC binds CYLD during *Lm* infection

To understand the role of InlC-CYLD binding during infection, we complemented ∆*inlC* strains with InlC mutants defective in CYLD binding (Fig. 2f). Using tools previously described^27^, FLAG epitope-tagged mutants of *inlC* were stably integrated into the tRNA^Arg^ locus of the *Lm* chromosome. These strains were then used for infection of bone marrow-derived macrophages, major contributors of host defense amidst listeriosis^28^. Following 6.5 h post-infection, immunoprecipitation of InlC-FLAG recovered endogenous CYLD, whereas this interaction was ablated in strains harboring InlC I159D or K236A (Fig. 2g). As expected, CYLD-binding defective mutants of InlC retained binding to IKK-α. In contrast, the F146A mutant retained binding to CYLD but was defective in binding IKK-α. These interactions were also observed in *Lm*-infected HeLa cells (Supplementary Fig. 4d), indicating they are not specific to macrophages.

### InlC promotes CYLD recruitment to ubiquitinated *Lm* in the cytosol of host cells

**Fig. 3:**
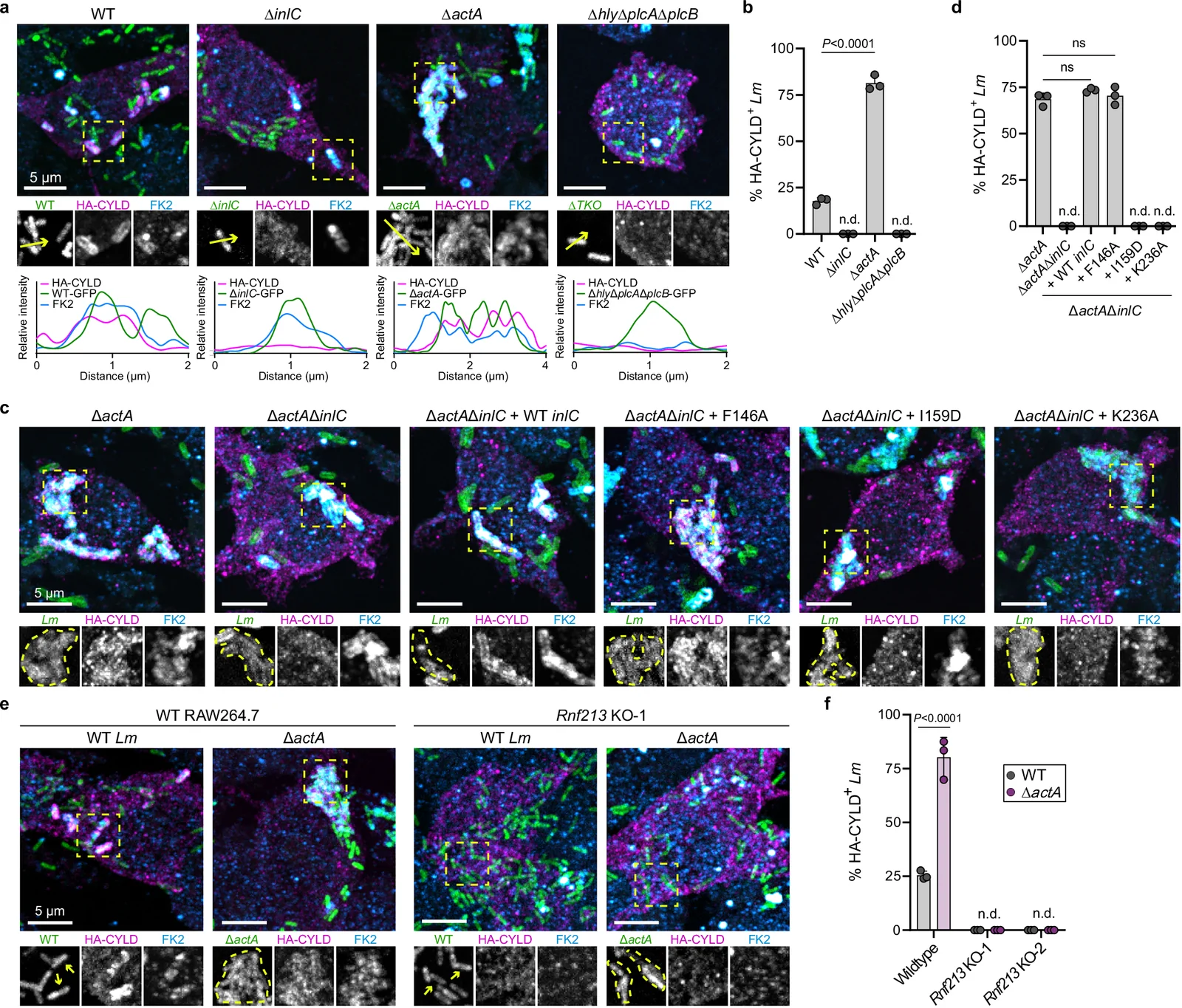
InlC targets CYLD to ubiquitinated *Lm* in the cytosol of host cells in a RNF213-dependent manner. **a**, **b** HA-CYLD recruitment in *Lm*-infected RAW264.7 macrophages (4 h post-infection). Scale bar, 5 μm. Relative intensity profiles correspond to the arrow in the *Lm* channel. **b** Quantification of HA-CYLD-positive bacteria per transfected cell. Data are presented as mean values ± SD based on quantification of *N* = 3 biological replicates (n.d. = not detected). Per biological replicate, the number of transfected, infected RAW264.7 cells analyzed ranged from 23 to 27 (WT), 20 (Δ*inlC*), 22 to 23 (Δ*actA*), and 20 (Δ*hly*Δ*plcA*Δ*plcB*). *P* values were determined using a one-way ANOVA with Dunnett’s post-hoc test. The exact *P* value between the WT and Δ*actA* condition was determined to be 0.000015. **c**, **d** HA-CYLD recruitment is dependent on InlC binding in RAW264.7 cells (4 h post-infection). Scale bar, 5 μm. Dashed lines highlight clusters of non-motile bacteria within the region of interest. **d** Quantification of HA-CYLD-positive bacteria per transfected cell. Data are presented as mean values ± SD based on quantification of *N* = 3 biological replicates (n.d. = not detected). For WT and Δ*inlC* + *inlC* conditions, the number of transfected, infected cells analyzed ranged from 20 to 21 per biological replicate. For all other strains tested, 20 transfected, infected cells were quantified per biological replicate. *P* values were determined using a one-way ANOVA with Dunnett’s post-hoc test (ns = not significant). **e**, **f** HA-CYLD recruitment is abolished in *Rnf213* knockout RAW264.7 cells (4 h post-infection). Scale bar, 5 μm. Arrows/dashed lines highlight bacteria within the region of interest. **f** Quantification of HA-CYLD-positive bacteria per transfected cell. Data are presented as mean values ± SD based on quantification of *N* = 3 biological replicates (n.d. = not detected). For wild-type RAW264.7 cells, the number of transfected, infected cells analyzed ranged from 22 to 24 for WT *Lm*, and 15 to 21 for Δ*actA Lm* per biological replicate. For both *Rnf213* KO clones, 20 transfected, infected cells were quantified per biological replicate. *P* values were determined using a two-way ANOVA with Tukey’s post-hoc test. The exact *P* value between the WT and Δ*actA* condition was determined to be 0.0000065. Source data are provided as a Source Data file.

We then visualized transiently expressed HA-CYLD during *Lm* infection of RAW264.7 macrophages. In WT *Lm*-infected cells at 4 h post-infection, HA-CYLD was recruited to a subset of bacteria (17%), where it co-localized with ubiquitinated proteins detected by the FK2 monoclonal antibody (Fig. 3a, b). Recruitment of HA-CYLD to bacteria was not observed in ∆*inlC* or a triple mutant strain (∆*hly*∆*plcA*∆*plcB*) that is unable to escape the phagosome. Prior studies demonstrated that the non-motile strain (Δ*actA*) is heavily ubiquitinated in the cytosol of host cells^2,10^. Consistent with this, we observed a significant increase in HA-CYLD recruitment to Δ*actA* mutant bacteria (81%) (Fig. 3b). A time course experiment revealed that InlC accumulation in host cells coincided with the emergence of HA-CYLD-positive Δ*actA* bacteria at 3–4 h post-infection (Supplementary Fig. 5a–c). In both the case of WT and Δ*actA* infection, all HA-CYLD-positive bacteria were found to be positive for ubiquitin (Supplementary Fig. 5d).

Given that HA-CYLD recruitment to ubiquitin-positive *Lm* coincided with InlC accumulation, we hypothesized that InlC promotes CYLD recruitment to a cytosolic population of bacteria upon recognition by the host ubiquitin system. To test this, we utilized the Δ*actA* mutant to exploit the prevalence of HA-CYLD recruitment during infection. A double knockout strain (∆*actA*∆*inlC*) revealed the essential role of InlC in recruiting CYLD to ubiquitinated bacteria (Fig. 3c, d). Complementation of the ∆*actA*∆*inlC* strain with either wild-type *inlC* or the F146A mutant restored HA-CYLD recruitment to bacteria; however, CYLD-binding defective (I159D, K236A) mutants of InlC could not complement this recruitment. Using an antibody to detect endogenous CYLD, we confirmed its recruitment to the bacterial surface in an InlC-dependent manner (Supplementary Fig. 6). Consistent with our structural observations, mutation of the CAP-Gly2 domain was also sufficient to disrupt HA-CYLD recruitment to Δ*actA* mutant *Lm* (Supplementary Fig. 7a). InlC-dependent HA-CYLD recruitment was also observed in mouse embryonic fibroblasts, a cell type previously used to study host ubiquitin defenses against *Lm*^2^ (Supplementary Fig. 7b). Thus, InlC promotes CYLD recruitment to ubiquitinated *Lm* in the cytosol of host cells.

### InlC does not accumulate alongside CYLD at the bacterial surface

Next, we asked if InlC also accumulates at the surface of ubiquitin-positive bacteria. For this, we infected cells with *Lm* expressing pLOV-*inlC*-FLAG for complementation of Δ*inlC Lm* with either wildtype *inlC* or single-point mutants defective in binding to CYLD (I159D, K236A). At 4 h post infection, cells were fixed and stained for the FLAG epitope to visualize the localization of InlC-FLAG. In accordance with previous studies^13,21^, we observed a diffuse, cytosolic signal for InlC that was above the background of the FLAG epitope antibody observed in cells infected with *Lm* transformed with an empty vector (i.e., no FLAG-tag expressed) (Supplementary Fig. 8a). However, we did not observe enrichment of InlC at ubiquitin-positive bacteria, including non-motile *Lm* (Δ*actA*Δ*inlC*). Similar cytosolic staining was observed in *Lm* strains expressing either WT InlC or CYLD-defective binding mutants, suggesting that InlC localization in the cytosol is unaltered by CYLD binding. Accordingly, we did not detect co-enrichment of InlC alongside HA-CYLD at ubiquitin-positive bacteria using both motile and non-motile *Lm* (Supplementary Fig. 8b). Taken together, these results suggest that InlC does not accumulate at the surface of ubiquitin-positive *Lm* alongside CYLD.

### The B-box module of CYLD is required for its recruitment to ubiquitin-positive bacteria

The absence of InlC enrichment at CYLD-positive bacteria led us to question how CYLD is recruited to cytosolic *Lm*. We postulated that beyond InlC binding, there may be intrinsic factors of CYLD required for successful delivery and/or retention at the bacterial surface. Within CYLD’s USP domain lies a zinc-binding B-box module (Supplementary Fig. 9a) that permits intermolecular interactions and partially regulates CYLD localization^23,29^. In uninfected RAW264.7 macrophages, HA-CYLD retained cytosolic localization upon deletion of this module (Supplementary Fig. 9b). While deletion of this module did not impair InlC-CYLD binding (Supplementary Fig. 9c), we observed impaired recruitment of HA-CYLD^ΔB-box^ to ubiquitin-positive Δ*actA* bacteria (Supplementary Fig. 9d). A lack of recruitment was also observed with truncated versions of HA-CYLD lacking either its USP or CAP-Gly domains, despite the former retaining InlC-CAP-Gly2 binding (Supplementary Fig. 9c, d). These findings suggest a co-requirement of InlC-CAP-Gly2 binding and the presence of CYLD’s B-box module for InlC-dependent CYLD recruitment to the bacterial surface.

### CYLD is recruited to the bacterial surface prior to actin-based motility

Previous studies highlighted the critical role of ActA in evading host-ubiquitin defenses^10,30,31^. As Δ*actA* bacteria are vulnerable to detection by host ubiquitin defenses, we questioned the motility status of CYLD-positive WT *Lm*. Co-staining of HA-CYLD alongside filamentous actin (F-actin) revealed that the majority of CYLD-positive WT *Lm* were surrounded by non-polarized actin (referred to as actin clouds) (87%), indicating that the bacteria were not yet motile (Supplementary Fig. 10a, b). Only a small fraction of CYLD-positive *Lm* was devoid of polymerized actin (11%), and in rare instances, displayed a comet tail (<2%). In agreement with these observations, we found that actin clouds were present at most ubiquitin-positive WT and Δ*inlC* bacteria (Supplementary Fig. 10c, d). Notably, amongst motile strains we observed a modest yet significant increase (12%) in the frequency of ubiquitin-positive bacteria upon loss of InlC (Supplementary Fig. 10e). These observations led us to speculate that the InlC-dependent recruitment of CYLD to the bacterial surface serves to accelerate ubiquitin clearance prior to actin-based motility.

### CYLD catalyzes Lys63-linked deubiquitination at the bacterial surface

To determine the functional impact of CYLD recruitment to ubiquitinated bacteria, we performed CRISPR-mediated knockout of *Cyld* in RAW264.7 cells (Supplementary Fig. 11a) and complemented these cells by expression of either wildtype (Supplementary Fig. 11b, upper panels) or catalytically inactive HA-CYLD (C601A) (Supplementary Fig. 11b, lower panels). Macrophages were then infected with Δ*actA* bacteria, fixed and stained for total ubiquitinated proteins (FK2^+^), Lys63-linked ubiquitin, and Met1-linked ubiquitin as indicated (Supplementary Fig. 11b). While both forms of HA-CYLD were recruited to bacteria at a similar frequency (Supplementary Fig. 11c), we measured a significant decrease of Lys63-linked ubiquitin staining in wildtype HA-CYLD-transfected cells compared to catalytically inactive HA-CYLD (Supplementary Fig. 11d). In contrast, total ubiquitinated proteins or Met1-linked ubiquitin were unaffected by wildtype HA-CYLD recruitment to bacteria. These findings suggest that CYLD recruited to the bacterial surface is catalytically active, with a preference towards deubiquitinating Lys63-linkages.

### CYLD recruitment to *Lm* requires the host E3 ligase RNF213

The E3 ligase RNF213 plays a major role in ubiquitin-dependent cellular defenses against cytosolic pathogens and is known to promote ubiquitination events on the surface of *Lm*^2,11^. Given that HA-CYLD was recruited to ubiquitin-positive *Lm*, we hypothesized that RNF213 may be required. To test this possibility, we performed CRISPR-mediated knockout of *Rnf213* in RAW264.7 cells (Supplementary Fig. 12a) and found these cells were unable to accumulate ubiquitin on the surface of Δ*actA* mutant bacteria (Supplementary Fig. 12b, c), consistent with previous findings^2^. In *Rnf213* KO RAW264.7 cells, we also did not observe HA-CYLD recruitment to either WT or Δ*actA* bacteria (Fig. 3e, f). Thus, RNF213 is required for the InlC-dependent recruitment of CYLD to cytosolic *Lm*.

### InlC-CYLD binding contributes to *Lm* virulence during systemic infection

In agreement with original findings in macrophages^12^, we observed that loss of InlC did not affect the growth of motile or non-motile *Lm* strains in RAW264.7 cells (Supplementary Fig. 13a, b). While RNF213-mediated defenses can be augmented upon interferon stimulation^32^, we similarly did not observe an InlC-dependent impact on bacterial growth in IFN-γ-primed cells (Supplementary Fig. 13a, b). We also observed that loss of InlC did not affect *Lm* cell-to-cell spread in either RAW264.7 cells (Supplementary Fig. 13c, d) or MEFs (Supplementary Fig. 13e, f), including untreated or IFN-γ-primed cells. These findings led us to speculate that InlC-CYLD binding may counteract host defenses encountered in vivo.

Prior studies established that *Rnf213* knockout mice are more susceptible to *Lm* infection^11^, indicating an important role for host ubiquitin defenses during systemic disease. In addition, optimal *Lm* virulence in mice was found to require InlC^12,33^. Building on this knowledge, we sought to determine the contribution of InlC-CYLD binding to *Lm* virulence using a murine model of systemic listeriosis.

**Fig. 4:**
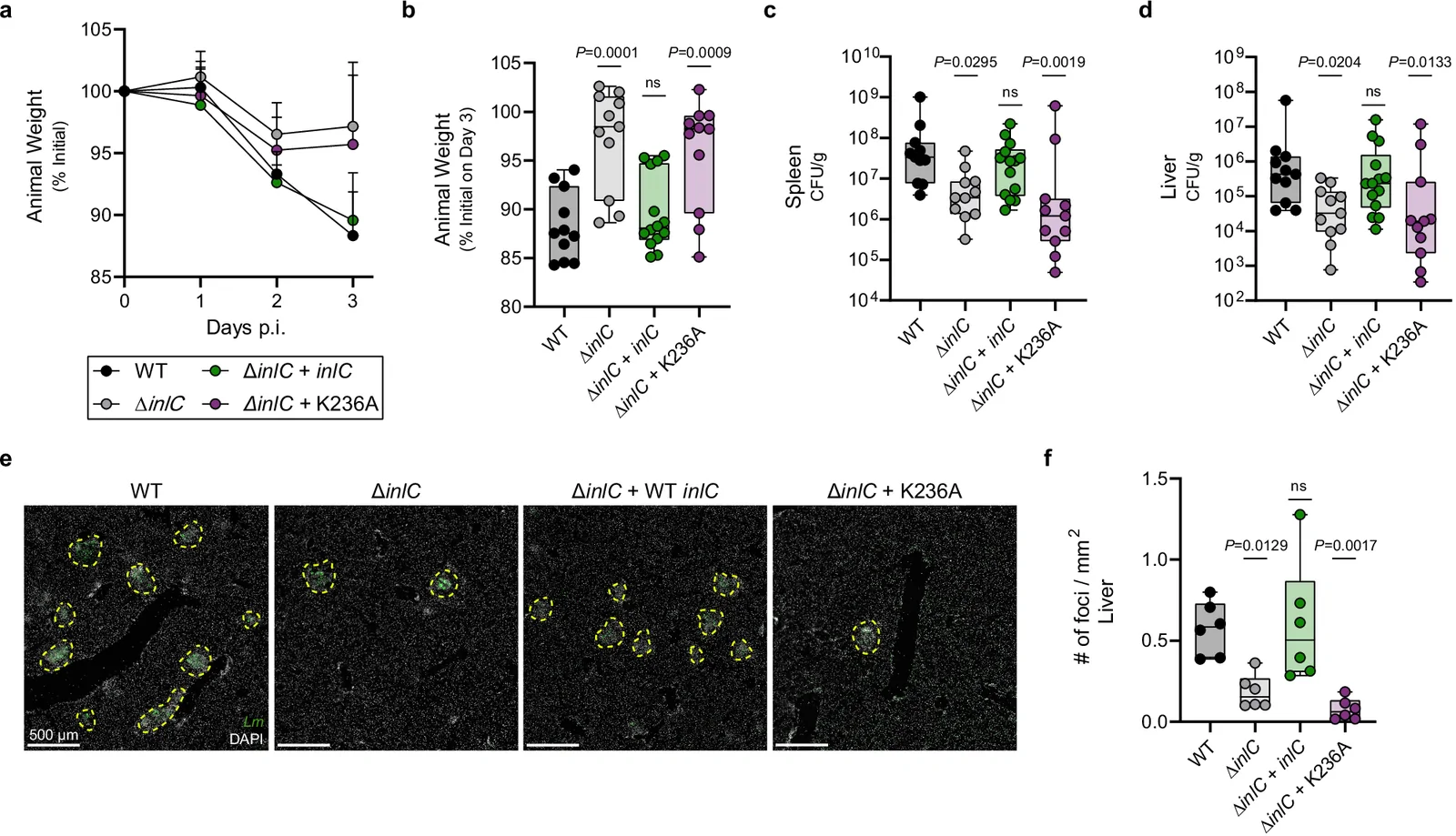
InlC-CYLD binding promotes *Lm* virulence during systemic infection. **a**, **b** Average weight of C57BL/6N mice systemically challenged with *Lm* across two biological replicates (*N* = 11 mice for WT, Δ*inlC*, Δ*inlC* + K236A groups and *N* = 14 mice for the Δ*inlC* + *inlC* group). **b** Distribution of animal weights on day 3 post-infection. Box and whisker plots represent the 25th to 75th percentile (box) including the median (line), with minimum and maximum values outlined (whiskers) across two biological replicates. *P* values were determined using a one-way ANOVA with Dunnett’s post-hoc test (ns = not significant). Bacterial burden in the spleen (**c**) and liver (**d**) of mice on day 3 post-infection across two biological replicates (*N* = 11 mice for WT, Δ*inlC*, Δ*inlC* + K236A groups and *N* = 14 mice for the Δ*inlC* + *inlC* group). Box and whisker plots represent the 25th to 75th percentile (box) including the median (line), with minimum and maximum values outlined (whiskers) across two biological replicates. *P* values were determined using a one-way ANOVA with Kruskal–Wallis post-hoc test (ns = not significant). **e**, **f** Representative liver sections of mice from (A) with infection foci circled. Scale bar, 500 μm. **f** Quantification of infection foci per area of tissue (*N* = 6 mice, three mice from each of the two biological replicates). Box and whisker plots represent the 25th to 75th percentile (box) including the median (line), with minimum and maximum values outlined (whiskers) across two biological replicates. *P* values were determined using a one-way ANOVA with Dunnett’s post-hoc test (ns = not significant). Source data are provided as a Source Data file.

Animals were infected by tail vein injection with WT or ∆*inlC* strains complemented with either wildtype *inlC* or the K236A mutant. Animal weight was monitored over the course of infection (Fig. 4a). Three days post-infection, we observed significant weight loss in mice infected with WT (11.66%) or ∆*inlC* + *inlC* bacteria (10.42%) compared to groups infected with ∆*inlC* (2.84%) or ∆*inlC* + K236A (4.28%) (Fig. 4b). Higher bacterial burdens (~1 log) were also observed in the spleen and liver of animals infected with wildtype *inlC*-expressing strains (Fig. 4c, d). We then visualized the distribution of infection within the liver (Fig. 4e), a major site of *Lm* growth^6^. In accordance with animal weight loss and higher bacterial burden, we found that strains expressing wild-type *inlC* displayed a greater number of infection foci throughout the liver compared to Δ*inlC* or InlC K236A-expressing strains (Fig. 4f). Thus, we conclude that InlC-CYLD binding promotes optimal *Lm* virulence during systemic infection in mice.

## Discussion

**Fig. 5:**
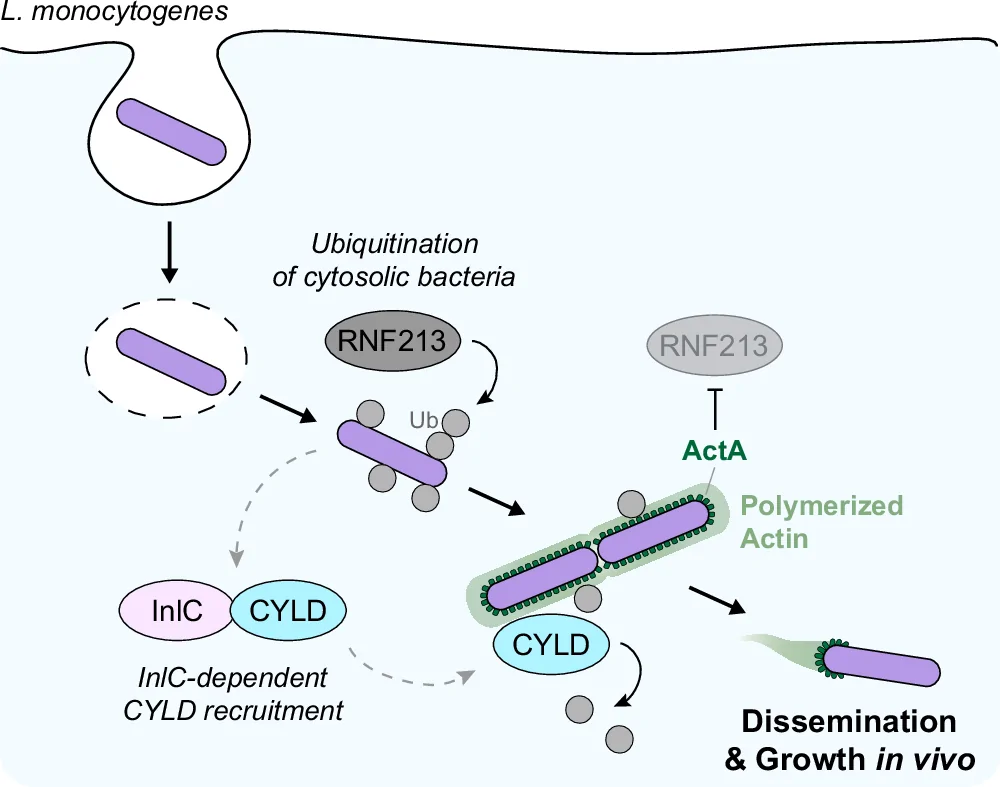
Model depicting the InlC-dependent recruitment of CYLD to the *Lm* surface to promote systemic disease. Upon internalization by a host cell, *Listeria monocytogenes* (*Lm*) undergoes phagosome escape through expression of a pore-forming toxin and phospholipase C enzymes. Entry into the cytosol permits RNF213-mediated ubiquitination of cytosolic bacteria. However, *Lm* can overcome this defense through secretion of InlC, which interacts with the CAP-Gly2 region of CYLD. InlC targets CYLD to the surface of ubiquitinated bacteria, where it can remove Lys63-linked ubiquitin. At the bacterial surface, ActA production also contributes to evasion of ubiquitin-mediated defenses. During systemic disease, InlC-CYLD binding promotes bacterial growth in the liver and spleen of mice.

Initial findings by Perrin et al. demonstrated the importance of ActA-mediated evasion from the host ubiquitin system^10^. Here, we reveal a cooperative role for InlC in the *Lm* strategy to counteract ubiquitin-mediated host defenses by promoting recruitment of the host deubiquitinase CYLD to the bacterial surface following recognition by the ubiquitin E3 ligase, RNF213 (Fig. 5). Despite the protection conferred by ActA, we postulate the need for a complementary evasion mechanism during the stage of infection following phagosome escape and prior to actin-based motility.

Our findings reveal that the LRR domain of InlC has two binding sites for its host protein targets. We propose that InlC acts through these host binding partners to execute different functions over the course of infection. Upon phagosome escape, bacteria are exposed to the cytosol and susceptible to innate detection such as RNF213-mediated ubiquitination^11^. To counter this, InlC binds CYLD to indirectly promote deubiquitinase recruitment to the bacterial surface. At later stages of infection, bacteria undergo actin-based motility to evade further recognition by the ubiquitin system. InlC then serves a second function, targeting TUBA through its SH3-6 domain to promote protrusion formation^14^. In this manner, InlC may serve as a protein scaffold, analogous to viral proteins such as Tax (HTLV-1)^34^ and Nsp1α (PRRSV)^35^, which control different aspects of the virus lifecycle.

How does InlC promote recruitment of CYLD to the bacterial surface? The lack of InlC enrichment at the surface of ubiquitin-positive bacteria suggests several possible mechanisms. First, InlC may traffic CYLD to the bacterial surface and then become rapidly displaced upon the association of CYLD with target substrates. Such a dynamic interaction of InlC with the bacterial surface would likely be undetected with the immunofluorescence microscopy approach applied in this study. Second, InlC-CYLD binding may operate through a displacement model, analogous to InlC-TUBA binding, which involves the displacement of N-WASP^14^. In this scenario, InlC binding to the CAP-Gly2 domain would displace CYLD from preexisting locations, such as microtubules and immune receptors^25,26^. In support of this mechanism, recent evidence highlights the peptide-binding abilities of CYLD’s CAP-Gly domains, which serve as auxiliary regions to regulate deubiquitinase localization^36^. A major limitation in our ability to assess this displacement model arises from the use of CYLD overexpression. Live-cell imaging of endogenously tagged CYLD may elucidate whether InlC promotes the recruitment of distinct populations of CYLD. Third, it remains possible that another host or bacterial factor is required to translocate CYLD to the bacterial surface. The inability of InlC to promote the recruitment of a truncated form of CYLD comprising only CAP-Gly1-3, despite proper binding, revealed a requirement for CYLD’s C-terminus for successful recruitment. In the context of immune signaling, the B-box-dependent dimerization of CYLD permits binding to SPATA2, an adapter protein that facilitates CYLD recruitment to immune receptors^23^. Our identification of the B-box as a necessary determinant for CYLD translocation suggests a similar role for CYLD dimerization and additional host factors to mediate CYLD delivery.

Here, we identify RNF213 as a critical host determinant for the ubiquitination of *Lm* in macrophages. While RNF213 possesses a RING domain, coating of *Lm* has been demonstrated to occur non-canonically through an RZ finger domain^2^. In the case of gram-negative bacteria, RNF213-mediated ubiquitination leads to the recruitment of additional E3 ligases such as the linear ubiquitin chain assembly complex (LUBAC)^3^. Recent evidence suggests that ubiquitination by RNF213 is the predominant cue for CYLD recruitment to *Lm* rather than ensuing E3 ligases such as LUBAC^37^. The ability of RNF213 to interact with, and even ubiquitinate CYLD^38,39^ raises the possibility of interplay between these two proteins at the bacterial surface. Whether RNF213 has scaffolding functions at the bacterial surface independent of its E3 ligase activity remains an area of future exploration.

Other intracellular pathogens can disrupt ubiquitin-mediated cellular defenses through secreted virulence proteins. For example, *Chlamydia trachomatis* secretes a deubiquitinase (Cdu1)^40^ that acts cooperatively with GarD^32^ to protect pathogen-containing inclusions from RNF213-mediated ubiquitination. *Shigella flexneri* translocates a member of the IpaH family of bacterial E3 ubiquitin ligases into host cells to selectively target RNF213 for degradation^4^. During *Burkholderia pseudomallei* infection, a secreted deubiquitinase (TssM) reverses RNF213-dependent ubiquitination^5^. The host and bacterial factors subject to ubiquitination/deubiquitination reactions during *Lm* infection remain unknown. RNF213 is known to ubiquitinate lipopolysaccharide on the surface of *Salmonella enterica* serovar Typhimurium^3^, a target lacking on gram-positive bacteria. It remains plausible that an abundant lipid species of the *Listeria* cell wall, such as teichoic acids, is a substrate of RNF213 activity. Our findings indicate that during *Lm* infection, CYLD acts to selectively reverse RNF213-dependent ubiquitination events with a preference for Lys63-linkages, akin to CYLD’s role at innate receptors^23^. Whether CYLD catalyzes the removal of Lys63-linked ubiquitin attributed to RNF213 activity^41^ or ensuing E3 ligases remains to be elucidated. InlC-CYLD binding may also have additional roles beyond the bacterial surface.

*Cyld*^−/−^ mice are known to be protected from systemic *Lm* infection^42^. This protection is thought to be conferred by CYLD’s homeostatic role in the regulation of ubiquitin-dependent immune responses^43^. In support of this notion, *Cyld*^−/−^ mice display enhanced inflammation, reduced cell apoptosis and impaired intracellular growth of *Lm*^42,44,45^. Here, using site-specific binding mutants of InlC, we show that InlC-CYLD binding is required for full virulence of *Lm* in mice. During listeriosis, immune cells express an isoform of CYLD lacking the CAP-Gly3 domain to augment immune signaling^46,47^. Our results suggest *Lm* exploits the conserved nature of the CAP-Gly2 domain to retain InlC-CYLD binding and bypass this host adaptation.

Lastly, our in vivo findings suggest InlC-CYLD binding contributes to *Lm* virulence. We propose that InlC-dependent CYLD recruitment promotes bacterial fitness against ubiquitin-mediated defenses encountered in vivo. We speculate that InlC promotes bacterial survival within primary-infected regions during the initial stages of infection, resulting in more dissemination events as infection proceeds. The importance of establishing multiple foci for virulence is reflected in clinical observations of listeriosis where increased focus number is associated with a poorer prognosis^48^. We conclude that CYLD is relevant to *Lm* infection for both its homeostatic role in immune regulation and as a specific target of a bacterial virulence factor. As such, our findings provide further insight into how intracellular pathogens modulate host ubiquitin defenses.

## Methods

### Cell lines

HeLa (#CCL-2, female, human), HEK293T (#CRL-3216, female, human), RAW264.7 (#TIB-71, male, murine), Mouse Embryonic Fibroblasts (MEFs) (#CRL-2991, murine), and L929 cells (#CCL-1, male, murine) were obtained from the American Type Culture Collection (ATCC) except for HEK293 Flp-In T-REx cells used for BioID (Invitrogen, R78007, female, human). All cell lines were grown at 37 °C in 5% CO_2_ and maintained in high-glucose DMEM (Wisent, #319-005) supplemented with 10% FBS (Wisent, #090-450) except for RAW264.7 cells (5% FBS). Cellstripper (Corning, #25-056-Cl) was used for the detachment of RAW264.7 cells during passaging. All cell lines were used for no more than 20 passages and routinely assessed for the absence of *Mycoplasma*. All cell lines used in this study can be found in Supplementary Data 2.

### Primary macrophage generation

Bone marrow-derived macrophages (BMDMs) were obtained from dissected femurs and tibias of 6–12-week-old, male C57BL/6N mice (Strain #027, Charles River Laboratories). Cells were differentiated in polystyrene petri dishes for 6–7 days with supplemented media: RPMI (Wisent, #350-000) containing 10% FBS, 1% sodium pyruvate (Wisent, #600-100), 1% non-essential amino acids (Wisent, #321-011), 0.5 % β-mercaptoethanol (Gibco, #21985023), 1% Pen-Strep (Wisent, #450-201) and 10% L929-conditioned media (homemade). Differentiation media was replaced 72 h post-isolation and every 1–2 days as required, followed by plating for experiments in solely RPMI + 10% FBS. Filtered, conditioned media containing M-CSF was generated from confluent layers of L929 cells cultured in DMEM containing 10% FBS for 8 days and subsequently stored at −20 °C until use.

### Generation of CRISPR knockout cell lines

Parental RAW264.7 cells (ATCC, #TIB-71) were used to generate single cell-derived knockout lines (*Cyld*, *Rnf213*) via CRISPR-mediated genome editing^49^. For each gene, three mouse-specific sgRNAs targeting proximal exons were selected using CHOPCHOP^50^ and cloned (BbsI) into the GFP-containing vector, pX458 (Addgene, #172221). Upon sequence validation, RAW264.7 cells were transfected overnight with either a pool of sgRNAs or an empty vector as a control and subsequently prepared for FACS sorting. Single, GFP-positive cells were sorted into a 96-well plate and expanded for 2–3 weeks prior to assessing knockout efficiency. Loss of CYLD protein expression was confirmed by Western blot, whereas perturbation of RNF213 was assessed on the genomic level (gDNA PCR band shift) and functionally (impaired *Lm* ubiquitination). For depletion of CYLD in HEK293T cells (ATCC, #CRL-3216), human-specific sgRNAs were cloned into pX459 (Addgene, # 62988) and bulk selection was performed using puromycin for a period of 48 h.

### Bacterial strains

*Listeria monocytogenes* (*Lm*) strains used were generated in the 10403S background: Wildtype^51^, Δ*inlC* (DP-L7704), Δ*actA* (DP-L3078), Δ*actA*Δ*inlC* (DH-L2196), and Δ*hly*Δ*plcA*Δ*plcB* (DP-L2319). Electrocompetent bacteria, prepared according to prior methods^52^, were used for transformation with the chromosomal integration vector pLOV^27^ to generate fluorescent strains (bacterial codon-optimized GFPmut3) or complement *inlC*. Briefly, the full-length coding sequence of *inlC* (GenBank: X95822.1) was synthesized (BioBasics) and inserted downstream of the *hly* 5’ UTR sequence under the Hyper-SPO1 promoter. Control strains used for complementation studies (WT, Δ*inlC*, ∆*actA*, ∆*actA*Δ*inlC*) were transformed with an empty vector to confer chloramphenicol resistance (7.5 µg/mL). All strains were validated for *inlC* integration at the genomic level and comparable levels of InlC secretion during infection.

An in-frame deletion of *actA* in the Δ*inlC* background (DP-L7704) was generated via allelic exchange using the temperature-sensitive plasmid pKSV7, as previously described^53^. Briefly, the knockout plasmid (pKSV7-Δ*actA*) harbored in *E. coli* (DH-E2157) was electroporated into the Δ*inlC* strain, and transformants were selected on BHI agar containing chloramphenicol (7.5 μg/mL) at 30 °C. A single colony harboring pKSV7-Δ*actA* was inoculated into BHI broth with chloramphenicol and incubated at 41 °C for several passages to promote chromosomal integration. Integration was further selected by plating for single colonies on chloramphenicol-containing BHI agar at 41 °C. The resulting strain was then serially passaged at 30 °C without antibiotic to enrich for plasmid excision and chloramphenicol sensitivity. Successful allelic exchange was confirmed by PCR and western blot. All bacterial strains used in this study can be found in Supplementary Data 2.

### Plasmids, cloning, and transfection

For BioID experiments, *inlC* was amplified from WT *Lm* (10403S) and inserted into pcDNA5-InlC-FlagBirA* (R118G)- FRT/TO by restriction cloning (BsrGI/NotI), whereas pcDNA5-FlagBirA* was used as a control. AlphaFold2^54^ was used to generate a predicted structure of InlC-BirA*. For immunofluorescence and pulldown experiments, HA-CYLD (Addgene #15506) was used as a template for site-directed mutagenesis and restriction enzyme-based cloning of domain truncations (FseI/AscI). eGFP-InlC (aa. 35–297) was previously described^33^, and peGFP-N1 (Clontech) served as a control for pulldown studies. Transfection of HEK293T and RAW264.7 cells was achieved using GeneJuice (Sigma-Aldrich) or FuGene HD (Promega), respectively, at a ratio of 3:1. For complementation studies, pLOV*-inlC* (aa. 1–297) and pLOV*-inlC-*FLAG (BioBasics) were used for site-directed mutagenesis. Cloning was performed using PCR conditions adapted to Q5 Polymerase (NEB, E0554). All plasmids, primers (Sigma-Aldrich), and cloning methods used in this study can be found in Supplementary Data 3.

### BioID of InlC-BirA*

AlphaFold2^54^ was used to generate a predictive structure of InlC-BirA* based on the coding sequence of the pcDNA5-InlC-FlagBirA* (R118G) vector generated. The coding sequence is comprised of the secreted form of InlC (PDB: 1XEU), a triple alanine linker, and BirA (PDB: P06709) with an R118G mutation. Version 1 of the model was last accessed on January 19th 2026, through the following https://alphafoldserver.com/fold/55b0453bd9b4eab4. Different views of the model, including pIDDT and PAE scores, can be found in Supplementary Fig. 1.

Stable HEK293 T-REx cells carrying pcDNA5-InlC-FlagBirA*- FRT/TO were prepared using the accessory plasmid pOG44 (Invitrogen, V600520) as per the manufacturer’s instructions, followed by selection with hygromycin B (200 μg/mL). For induction and labeling, tetracycline (1 μg/mL) and biotin (50 μM) were added to the culture medium, respectively, for a 20 h period^19^. Cell pellets were resuspended in 10 mL lysis buffer (50 mM Tris-HCl pH 7.5, 150 mM NaCl, 1 mM EDTA, 1 mM EGTA, 1% Triton X-100, 0.1% SDS) containing 1:500 protease cocktail (Sigma-Aldrich, #P8340) and Benzonase (25 U/mL, Novagen), sonicated, and clarified by centrifugation (30 min, 16,000 × *g*, 4 °C). Supernatants were transferred to a 15 mL tube containing 30 μL streptavidin sepharose beads (GE HealthCare) and incubated for 3 h at 4 °C with end-over-end rotation. Beads were rinsed 6 times with 1 mL of 50 mM ammonium bicarbonate (pH = 8.3) followed by tryptic digestion (1 μg, Promega, #V5111) overnight at 37 °C. Following incubation, an additional 0.5 μg trypsin was added for 2 h at 37 °C. Supernatants were collected, beads were rinsed with 50 mM ammonium bicarbonate, and eluates were pooled. Samples were desalted using C18 tips, lyophilized, and resuspended in 0.1% formic acid prior to LC-MS analysis.

### LC-MS/MS

Analytical columns (75-μm inner diameter) and pre-columns (150-μm inner diameter) were made in-house from fused silica capillary tubing from InnovaQuartz and packed with 100 Å C18-coated silica particles (Magic, Michrom Bioresources). Digested, de-salted and lyophilized peptides were subjected to liquid chromatography-electrospray ionization-tandem mass spectrometry (LC-MS/MS) using a 120 min reversed-phase (100% water to 100% acetonitrile, 0.1% formic acid) buffer gradient running at 250 nL min^−1^ on a Proxeon EASY-nLC pump in-line with a hybrid LTQ-Orbitrap Velos mass spectrometer (ThermoFisher). A parent ion scan was performed in the Orbitrap using a resolving power of 60,000, then up to 20 of the most intense peaks were selected for MS/MS (minimum ion count of 1000 for activation), using standard collision-induced dissociation fragmentation. Fragment ions were detected in the LTQ. Dynamic exclusion was activated such that MS/ MS of the same m/z (within a range of 15 ppm; exclusion list size = 500) detected twice within 15 s were excluded from analysis for 30 s. For protein identification, Thermo.RAW files were converted to the.mzXML format using Proteowizard54, then searched using iProphet55 against the human (Human RefSeq v.45) database. X!Tandem search parameters were: 15 ppm parent mass error; 0.4 Da fragment mass error; complete modifications, none; cysteine modifications, none; potential modifications, +16@M and W, +32@M and W, +42@N-terminus, +1@N and Q. Each of the two biological replicates was analyzed using two technical replicates. Data were analyzed using the Trans Proteomic Pipeline56, via the ProHits software suite57. Proteins identified with a Protein Prophet cut-off of 0.9 and at least two unique peptides were analyzed with the SAINT Express algorithm (v.3.6.1). Twelve control runs (consisting of Flag BirA*only) were collapsed to the two highest spectral counts for each prey, and the SAINT score cut-off value was set to a Bayesian false discovery rate (BFDR) < 0.01 (1% FDR). All raw data are available through the MassIVE repository under accession #: MSV000100210.

### Protein expression and purification

Mutants of InlC (aa. 35–297), CYLD CAP-Gly2 (aa. 229–313), and TUBA SH3-6 (aa. 1512–1577) were cloned into pOPINB (Addgene, #41142) for *E.coli* expression. pOPINB plasmids were transformed into Rosetta BL21 *E.coli* (Novagen, #70954), and cultures were grown in 2xTY medium supplemented with kanamycin (30 μg/mL) and chloramphenicol (34 μg/mL). Expression was induced with 400 μM IPTG at 18 °C overnight, followed by pelleting at 3990 × *g*.

InlC, CYLD CAP-Gly2 mutants and TUBA SH3-6 were purified by resuspending *E.coli* pellets in lysis buffer: 20 mM Tris pH 8−8.5, 500 mM NaCl, 20 mM Imidazole, 2 mM β-mercaptoethanol supplemented with lysozyme (Sigma-Aldrich, #L6876), DNase (Sigma-Aldrich, #AMPD1), 2 µM Leupeptin (BioShop), 1 µM Pepstatin (BioShop) and 1 mM PMSF (BioShop) and lysed by sonication. Following clarification of the lysate, supernatants were subjected to metal affinity chromatography (Hi-Trap Cytiva) and eluted in buffer containing: 20 mM Tris pH 8.5, 500 mM NaCl, 2 mM β-mercaptoethanol, and 500 mM Imidazole. His_6_ tags were removed by 3C protease. InlC and CYLD CAP-Gly2 mutants were further purified by anion exchange chromatography (Resource Q, Cytiva) in 20 mM Tris pH 8.5, 4 mM DTT, eluting with 1 M NaCl across a linear gradient. Appropriate fractions were pooled, concentrated, and further purified by size exclusion chromatography in 20 mM HEPES pH 7.5, 200 mM NaCl, 2 mM TCEP (HiLoad Superdex 75, Cytiva). TUBA SH3-6 was subjected to cation exchange chromatography (Resource S, Cytiva) in 20 mM MES pH 6.5, 20 mM NaCl, 4 mM DTT to purify TUBA SH3-6 from contaminants that bound the Resource S column. TUBA SH3-6 was then further purified by two rounds of size exclusion chromatography in 20 mM HEPES pH 7.5, 200 mM NaCl, 2 mM TCEP (HiLoad Superdex 75, Cytiva).

Full-length CYLD (aa. 1–956) was cloned into a modified pACEBac1 vector as described^55^ for expression in Sf9 cells. One litre of Sf9 cells was infected with 12–15 mL of P1 virus and harvested 3 days post-infection by pelleting at 3990 × *g* 4 °C and washed with PBS. The pellet was lysed in 20 mM Tris pH 8.5, 200 mM NaCl, 4 mM DTT, 10 % (v/v) glycerol supplemented with cOmplete Protease Inhibitor (Roche), DNase, 1 mM PMSF, and 0.5 % (v/v) Tween-20. Recombinant protein was enriched from clarified lysate using Strep-Tactin^®^-XT resin (IBA) followed by on-column elution through incubation with 3C protease at 4 °C overnight and washing with 20 mM Tris pH 8.5, 4 mM DTT. Eluate was then subjected to anion exchange chromatography (Resource Q, Cytiva) and further purified by size exclusion chromatography (HiLoad Superdex 200, Cytiva) in 20 mM HEPES pH 7.5, 200 mM NaCl, 4 mM DTT.

### Deubiquitination assays

Lys63- and Met1-linked tetra-ubiquitin chains were assembled as previously described^56^ and deubiquitination assays were performed accordingly^26^. Briefly, 10 nM full-length CYLD (aa. 1–956) was incubated with 100 nM InlC (aa. 35–297) and 0.08 mg/mL (2 μM) Lys63 Ub_4_ or Met1 Ub_4_ in assay buffer (50 mM Tris pH 7.4, 50 mM NaCl, 5 mM DTT) at room temperature. At indicated time points, samples were quenched with reducing 4× LDS sample buffer (Invitrogen) followed by SDS-PAGE gel electrophoresis and visualized by silver stain (Bio-Rad).

### Isothermal titration calorimetry

Measurements were performed in 20 mM HEPES pH 7.5, 200 mM NaCl, 2 mM TCEP. ITC data were collected using an Auto-iTC200 instrument (Malvern Instruments, Malvern, UK) at 25 °C. Sixty micromolar CYLD CAP-Gly2 mutants or TUBA SH3-6 in the calorimeter cell were titrated against 1 mM InlC mutants in the syringe using 19 injections of 2 µL preceded by a small 0.4 µl pre-injection. Experiments determining CYLD CAP-Gly2 affinity to InlC were performed in technical triplicate. The changes in heat release were integrated over the entire titration and fitted to a single-site binding model using the PEAQ Analysis software (Malvern Instruments), and graphs were plotted in Prism (GraphPad, v9).

### Crystallization data collection and refinement

InlC–CYLD CAP-Gly2 crystals were grown by sitting-drop vapor diffusion with 1.1 molar excess of CAP-Gly2. Crystals grew from conditions containing 10% (w/v) PEG 8,000, 100 mM Tris pH 7.0, and 200 mM MgCl_2_ and were transferred to a reservoir solution containing 28 % (v/v) glycerol prior to cryo-cooling. Diffraction data were collected at Diamond Light Source beamline I04 (wavelength = 0.95374 Å, temperature = 77 K). Diffraction images were processed using DIALS^57^ and manually scaled using AIMLESS^58^ as part of the CCP4 software package^59^. The structure of InlC bound to CAP-Gly2 was determined by molecular replacement using PHASER^60^, placing CAP-Gly2 and InlC (PDB IDs: 1WHM and 1XEU, respectively). Iterative rounds of model building and refinement were performed with COOT^61^ and PHENIX^62^, respectively. Data collection and refinement statistics can be found in Supplementary Table 1. All structure figures were generated with Pymol (www.pymol.org) and multiple sequence alignments of CAP-Gly2 were performed using Clustal Omega (EMBL-EBI).

### Western blotting

Cells were placed on ice, washed twice with cold PBS, and collected in lysis buffer supplemented with protease and phosphatase inhibitors (BioShop): 50 mM Tris-HCl pH 7.4, 150 mM NaCl, 2 mM EDTA, 1% Triton X-100, 2 µM Leupeptin, 1 mM PMSF, 1.5 µM Aprotinin, 1 µM Pepstatin A, 5 µM Na_3_VO_4_, and 5 µM NaF. Samples were rotated for 10 min at 4 °C, and the soluble fraction was isolated following max-speed centrifugation at 16,000 × *g*. Clarified lysate was then used for immunoprecipitation as noted below. All samples were resolved by SDS-PAGE (10% or 12%), transferred to PVDF membrane (Bio-Rad), and subsequently blocked with 5% skim milk in TBS-T (20 mM Tris, 150 mM NaCl, 0.1% Tween-20, pH 7.4). Incubation with primary antibody was performed overnight at 4 °C, followed by secondary antibody for 1 h at room temperature. Detection was achieved using Super Signal chemiluminescent substrates (Thermo, #34580, #34094) and images were acquired on a Gel Doc XR system equipped with Image Lab software (Bio-Rad, v6.1). All immunoblots are representative of findings from three biological replicates.

### Immunoprecipitation

For immunoprecipitation of eGFP-InlC, HEK293T cells were seeded in 6 cm dishes and transfected upon reaching 80% confluency. Following 36–48 h, cell lysates were prepared, and 20% (v/v) of the lysate was retained as the input fraction, whereas the remaining sample was rotated with pre-washed GFP-Trap resin (Abnova, #U0391) for 2 h at 4 °C. Beads were then pelleted at 2500 × *g* for 2 min, briefly washed three times in lysis buffer, and boiled in 100 µL 2x sample buffer to elute the bound fraction.

For immunoprecipitation of InlC-FLAG from primary macrophages, differentiated cells were seeded at 10 × 10^6^ cells per 10 cm dish and infected with an MOI = 1. The first hour of infection was performed in a total volume of 6 mL to avoid the need for centrifugation. At 6.5 h post-infection, cells were rinsed twice in cold PBS followed by the addition of 400 µL lysis buffer. Upon lysate clarification, 20% (v/v) was saved for input, and the remainder was incubated with 25 µL pre-washed Anti-FLAG^®^ M2 beads (Millipore, A2220) for 2 h at 4 °C. Beads were then briefly washed three times in lysis buffer, and bound proteins were eluted by boiling samples in 100 µL of 2× sample buffer. Identical methods were used for InlC-FLAG recovery from HeLa cells following a 20 h infection period (MOI = 10).

### GST binding assay

Recombinant GST, GST-InlC (aa. 35–297) and His-CAP-Gly2 (aa. 223–309) were expressed and purified from BL21 *E. coli* (NEB, #C2527). GST or GST-InlC (20 µg) were incubated with 15 µL Sepharose 4B GST-tagged resin (Cytivia, GE17-0756-01) in binding buffer (150 mM NaCl, 20 mM Tris pH 8.0, 1 mM MgCl2, 0.1% Triton X-100, 10% Glycerol) for 1 h at room temperature, followed by overnight blocking in 5% BSA at 4 °C. Beads were then briefly washed in binding buffer before the addition of His-CAP-Gly2 (5 µg) for 2 h at 4 °C. Following incubation, beads were pelleted, and the unbound sample was collected. Beads were then washed three times and eluted in glutathione-containing buffer as per the manufacturer’s instructions.

### *Lm* infection of cell lines

All *Lm* infections were set up as follows: overnight cultures in Brain Heart Infusion (BHI) were sub-cultured the next day (1:20), shaking at 37 °C, until the OD_600_ reached 0.3 (~1.5 h). Corresponding to ~3 × 10^8^ bacteria/mL, 1 mL of bacteria was pelleted and washed three times in PBS. Bacteria were then diluted in growth medium without serum to the corresponding multiplicity of infection (MOI) and added to cells. Unless noted otherwise, plates were centrifuged at 300 × *g* for 5 min to promote *Lm*-host cell contact. Following 1 h post-infection, complete medium was replaced to include gentamycin (50 µg/mL), and when required, mouse IFN-γ (100 ng/mL) (Biolegend, #575306).

For localization experiments in RAW264.7 cells, cells were seeded at a density of 0.25 × 10^6^ cells upon 12 mm glass coverslips in a 24-well plate. The following day, RAW264.7 cells were transfected for 16–20 h and subsequently infected with *Lm* (1–4 h). For transfection experiments, an MOI = 10 was used to increase the frequency of transfected, infected cells, whereas an MOI = 2.5 was used for experiments without transfection. For localization experiments in MEFs, cells were seeded until confluence, transfected for 16–20 h, and subsequently infected with *Lm* (MOI = 50) for 6 h.

For *Lm* cell-to-cell spread experiments, untreated or IFN-γ-treated (100 ng/mL) RAW264.7 cells and MEFs were seeded to confluence (2 × 10^6^ or 0.5 × 10^6^ cells, respectively) upon 25 mm glass coverslips in a 6-well plate. Following 24 h, RAW264.7 cells (MOI = 0.001) or MEFs (MOI = 0.035) were infected with *Lm* and subsequently fixed at 20 h post-infection.

For bacterial growth experiments, untreated or IFN-γ-treated (100 ng/mL) RAW264.7 cells were seeded at a density of 0.75 × 10^6^ per well in a 24-well plate. Following 24 h, cells were infected with *Lm* (MOI = 1) and subsequently lysed in 1 mL PBS containing 0.5% Triton X-100 at respective time points. Serial dilutions (1–10) were performed in micro test tubes and plated on BHI-agar plates to enumerate colony forming units (CFUs) following overnight incubation at 37 °C.

### Immunofluorescence and confocal microscopy

Cells were fixed in 2.5% paraformaldehyde for 15 min at 37 °C and incubated in staining buffer (1x PBS, 10% goat serum, 0.2% saponin) for 30 min at room temperature. Coverslips were then transferred to a humidified chamber for primary antibody staining overnight at 4 °C. The next day, secondary antibody was added for 1 h at room temperature, and coverslips were mounted in fluorescence mounting medium (Agilent, S3023). Samples were imaged on a spinning disk system (Quorum Technologies) comprised of a DMI6000 B inverted fluorescence microscope (Leica) equipped with a Hamamatsu CMOS FL-400 camera and Volocity v6.3.1 software. For localization studies, samples were imaged using a 63× oil immersion objective, and confocal z-stacks of 0.25 µm were acquired. Representative images were deconvoluted in Volocity using the fast restoration tool with respective point spread functions (Quorum). Images of endogenous CYLD and InlC-FLAG were not deconvoluted due to relatively weak signal. For *Lm* cell-to-cell spread studies, samples were imaged using a 10× air objective. Representative images from one of three biological replicates were assembled using ImageJ^63^, and the line selection tool was used to generate intensity profiles. Spline curves of relative fluorescence values, displayed as arbitrary units, were generated in Prism (GraphPad, v10.2.1).

### Immunofluorescence quantification

Confocal images of *Lm*-infected cells were analyzed in Volocity as follows. For quantification of HA-CYLD-positive *Lm* (%) (Fig. 3b, d, f and Supplementary Figs. 5c, 10b, 11c), individual bacteria were manually scored from randomly selected transfected cells. For quantification of ubiquitin (FK2)-positive *Lm* (%) (Supplementary Figs. 5d and 10d, e), individual bacteria were manually scored from transfected cells that displayed a ubiquitin response against *Lm* (>1 FK2+ bacteria). For quantification of ubiquitin (FK2)-positive Δ*actA* clusters (Supplementary Fig. 12c), (35–50) clusters were manually scored across (5–8) randomly selected fields of view.

For quantification of ubiquitin (FK2, Lys63-, or Met1-linked) intensity (Supplementary Fig. 11d), confocal planes were merged, and single clusters of HA-CYLD-positive bacteria were selected across (>15) individual cells per biological replicate. Using Volocity, a region of interest was determined based on *Lm*-GFP intensity, applying the dilation tool (1×) to account for ubiquitin signal surrounding the bacterial membrane. The sum of intensity for ubiquitin fluorescence was recorded and normalized to the area (μm^2^) of the region of interest.

For quantification of *Lm* cell-to-cell spread (Supplementary Fig. 13d, f), regions of interest were manually drawn around *Lm*-infected regions, and the area (μm^2^) was recorded using Volocity.

### Antibodies

Primary antibodies used for immunofluorescence with corresponding dilutions include rabbit anti-*Lm* (BD, #223021) (1:500), rabbit anti-HA-Tag (Cell Signaling, #C29F4) (1:200), mouse anti-HA-Tag (Cell Signaling, #2367) (1:200), mouse anti-FLAG-Tag (Cell Signaling, #8146) (1:200), mouse anti-CYLD (Santa-Cruz, #sc-74435, C-term.) (1:100), mouse anti-ubiquitin (Sigma-Aldrich, #04-263, FK2) (1:200), rabbit anti-Lys63 ubiquitin (Sigma-Aldrich, #05-1308, Apu3) (1:100), and rabbit anti-linear ubiquitin (Sigma-Aldrich, #ZRB2114, 1E3) (1:200). All secondary antibodies (1:200) were Alexa Fluor-conjugated (Thermo) and a Zenon labeling kit (Thermo, #Z25306 or #Z25308) was used to co-stain *Lm* alongside rabbit antibodies, when necessary. Nuclei were stained using 4′,6-diamidino-2-phenylindole (DAPI) (Thermo, #D1306), and Acti-stain 670 (Cytoskeleton Inc, #PHDN1) was used to visualize filamentous actin.

Primary antibodies for western blot (1:1000 unless otherwise noted) include mouse anti-GFP (Santa Cruz, #sc-9996) (1:10,000), mouse anti-CYLD (Santa-Cruz, #sc-74435, C-term.) rabbit anti-CYLD (Cell Signaling, #8462, N-term.), rabbit anti-FLAG (Cell Signaling, #14793), rabbit anti-SPATA2 (Bethyl, #A302-494A), rabbit anti-IKK-α (Cell Signaling, #61294), rabbit anti-IKK-β (Cell Signaling, #8943), mouse anti-IKKγ (Cell Signaling, #2695), mouse anti-p60 (Novus Biologicals, # NBP2-80120), rabbit anti-InlC (Gift from Dr. Keith Ireton, PMID: 19767742) and mouse anti-β-actin (Sigma-Aldrich, A5441) (1:10,000). All secondary antibodies (1:5000) were HRP-conjugated (Jackson ImmunoResearch). All antibodies used in this study for immunofluorescence and western blot can be found in Supplementary Data 4.

### *Lm* infection of mice

Wildtype, male C57BL/6 N mice were purchased from Charles River Laboratories (strain #: 027) and acclimatized for at least one week in a specific pathogen-free unit (22 °C, 40% humidity) maintained on a 12 h light-dark cycle with food and water available ad libitum. Animal experiments were strictly performed using male mice based on known sex-dependent differences in susceptibility to *Listeria monocytogenes*^64^. Intravenous infection (tail vein) experiments were performed with 8–9-week-old mice (22 ± 2 g) challenged with 5 × 10^4^ bacteria/mouse in a final volume of 200 µL PBS. Three days post-inoculation, animals were sacrificed by CO_2_ asphyxiation and tissues were harvested in PBS containing 0.1% Triton X-100 for bead homogenization. For each liver, the right medial lobe was used for homogenization, whereas the left later lobe was preserved in OCT compound (Tissue-Tek) for tissue sectioning. Tissue lysates were serially diluted (1–10) and spotted on BHI agar plates to enumerate CFU expressed per gram of tissue. All experiments described were carried out in accordance with the Guide for the Humane Use and Care of Laboratory Animals, and ethical approval was obtained from The Hospital for Sick Children’s Animal Care Committee (AUP #65606).

### Tissue sections

OCT-embedded livers were flash frozen upon dissection, and serial sections were performed using a cryostat (Leica, CM1860). Five tissue slices (6 μm) were systematically collected throughout the liver (250 μm apart) for 6 mice per group (3 mice from each biological replicate). Slides were fixed in ice-cold methanol for 10 min followed by three washes in 1× TBS. Tissues were encircled with a hydrophobic pen (Abcam, ab2601) and blocked for 30 min (1× TBS, 10% goat serum, 0.2% Triton X-100) prior to antibody staining. Slides were then scanned (3DHISTECH) and whole tissue area alongside bacterial foci were enumerated in CaseViewer software.

### Quantification and statistical analyses

Statistical analyses were conducted using Prism (GraphPad, v10.2.1). A *P*-value < 0.05 was considered statistically significant with annotations as follows: *P* < 0.05 (*), 0.001 < *P* < 0.01 (**), *P* < 0.001 (***), and *P* < 0.0001 (****). Where applicable, “ns” represents a comparison that is not statistically significant (*P* > 0.05) and “n.d.” denotes events that were not detected. With the exception of box and whisker plots (median ± SD), all graphs display the mean ± SD as determined by the respective statistical test described in the figure legend.

### Reporting summary

Further information on research design is available in the Nature Portfolio Reporting Summary linked to this article.

## Supplementary information

Supplementary Information

Description of Additional Supplementary Files

Supplementary Data 1

Supplementary Data 2

Supplementary Data 3

Supplementary Data 4

Reporting Summary

Transparent Peer Review File

## Source data

Source Data file

## Data Availability

The raw mass spectrometry data for the InlC-BirA* BioID generated in this study has been deposited to the MassIVE public repository (https://massive.ucsd.edu) under accession code #MSV000100210 [https://doi.org/10.25345/C5CN6ZC5P]. The protein structure of InlC-CYLD CAP-Gly2 has been deposited to the Protein Data Bank (PDB: 9TMK) [https://doi.org/10.2210/pdb9TMK/pdb]. All primers and cloning methods used to generate the constructs used in this study are provided in the Supplementary Data. Source data are provided with this paper. Requests for resources and reagents should be directed to corresponding author. Source data are provided with this paper.
