# Supplementary material for: Listeria promotes systemic infection by targeting the host deubiquitinase CYLD
Source: Nat Commun. 2026 Aug 31;17:10351. doi: 10.1038/s41467-026-77063-5 (PMC13623971; doi:10.1038/s41467-026-77063-5)
Supplement: Supplementary file 1 [file 41467_2026_77063_MOESM1_ESM.pdf]

SUPPLEMENTARY INFORMATION

Listeria promotes systemic infection by targeting the host deubiquitinase CYLD

Dustin A. Ammendolia[1,2], Cara J. Ellison[3], Yuelin Zheng[1,2], Etienne Coyaud[4], Estelle M.N. Laurent[4], Bing-Ru Yan[1], Bri Manning[5], Anna K. Waldmann[1,2], Joel M.J. Tan[1,6], Scott Frendo-Cumbo[1,7], Carina Lyons[1,2,8], Ji-Young Youn[1,2,8], Brian Raught[4,9], Keith Ireton[10], Darren E. Higgins[5], Paul R. Elliott[3], John H. Brumell[1,2,6,11*].

[1] Cell & Systems Biology Program, Hospital for Sick Children, Toronto, ON, M5G 1X8, Canada
[2] Department of Molecular Genetics, University of Toronto, Toronto, ON, M5S 1A8, Canada
[3] Department of Biochemistry, University of Oxford, Oxford, OX1 3QU, UK
[4] Princess Margaret Cancer Centre, University Health Network, Toronto, ON, M5G 2C4, Canada
[5] Department of Microbiology, Blavatnik Institute, Harvard Medical School, Boston, MA, 02115 USA
[6] Institute of Medical Science, University of Toronto, Toronto, ON M5S 1A8, Canada
[7] Department of Physiology, University of Toronto, Toronto, ON, M5S 1A8, Canada
[8] Molecular Medicine Program, Hospital for Sick Children, Toronto, ON, M5G 1X8, Canada
[9] Department of Medical Biophysics, University of Toronto, Toronto, ON, M5S 1A8, Canada
[10] Department of Microbiology and Immunology, University of Otago, Dunedin 9016, New Zealand
[11] SickKids IBD Centre, Hospital for Sick Children, Toronto, ON, M5G 0A4, Canada

[*]Corresponding author:

John H. Brumell, Cell & Systems Biology Program, Hospital for Sick Children, 686 Bay Street PGCRL, Toronto, ON, M5G 0A4, Canada Tel: 416-813-7654 ext. 303555. E-mail: john.brumell@sickkids.ca

Key Words: Bacterial pathogenesis, CYLD, deubiquitinase, Internalin C, *Listeria monocytogenes,* RNF213, ubiquitin

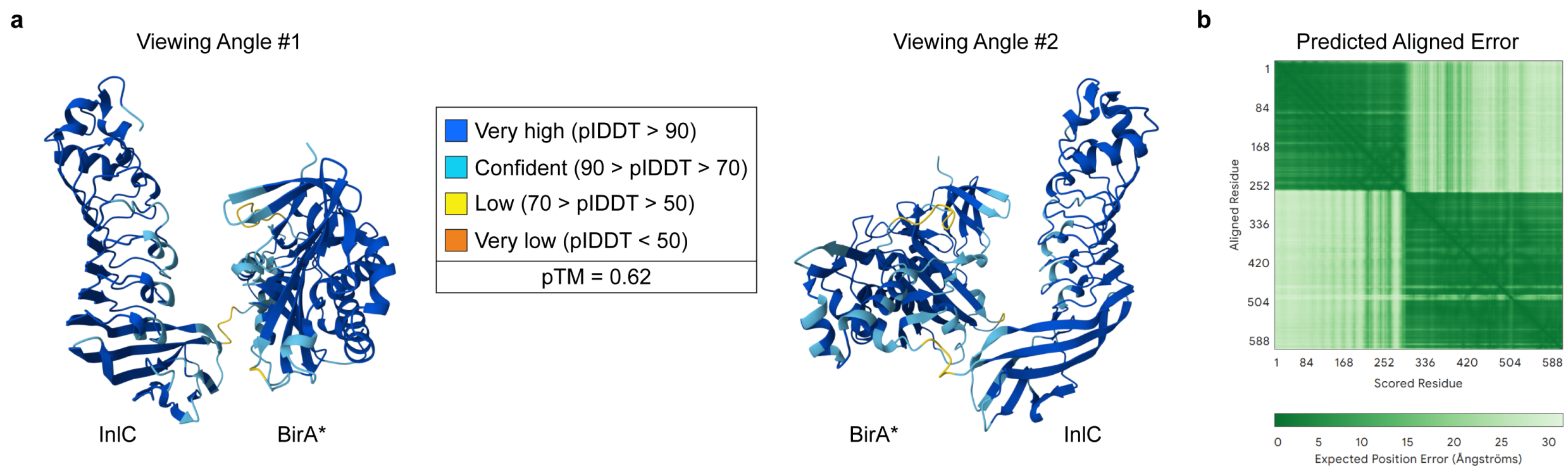

Supplementary Figure 1. AlphaFold2 structural prediction of InlC-BirA*.

a, Alternative viewing angles of InlC-BirA* with pIDDT scores superimposed and color-coded.

b, Predicted aligned error (PAE) matrix for the corresponding structure in (**a**), obtained from AlphaFold2.

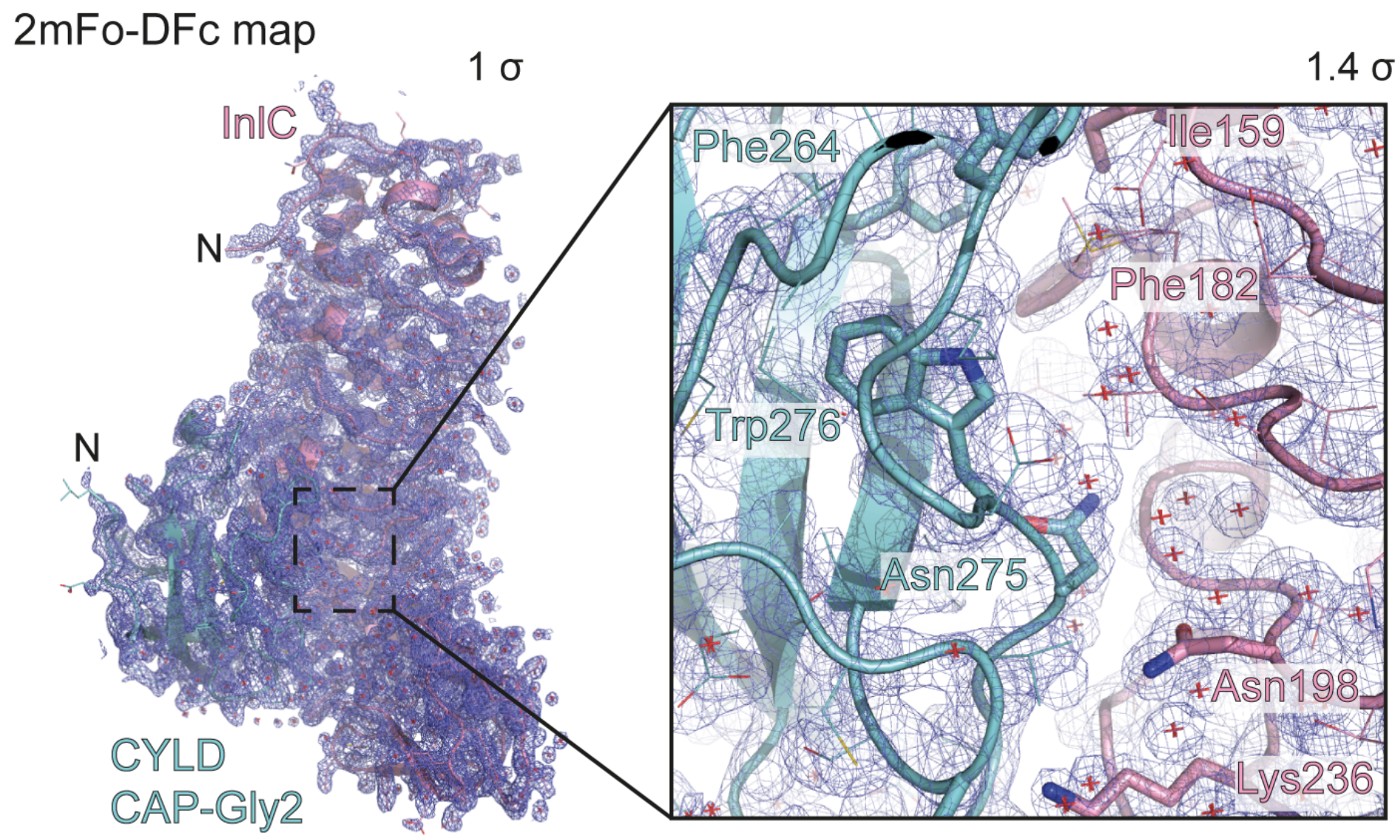

Supplementary Figure 2. Electron density map (2mFo-DFc) of InlC-CAP-Gly2 contoured at 1.0 σ (left) and 1.4 σ (right).

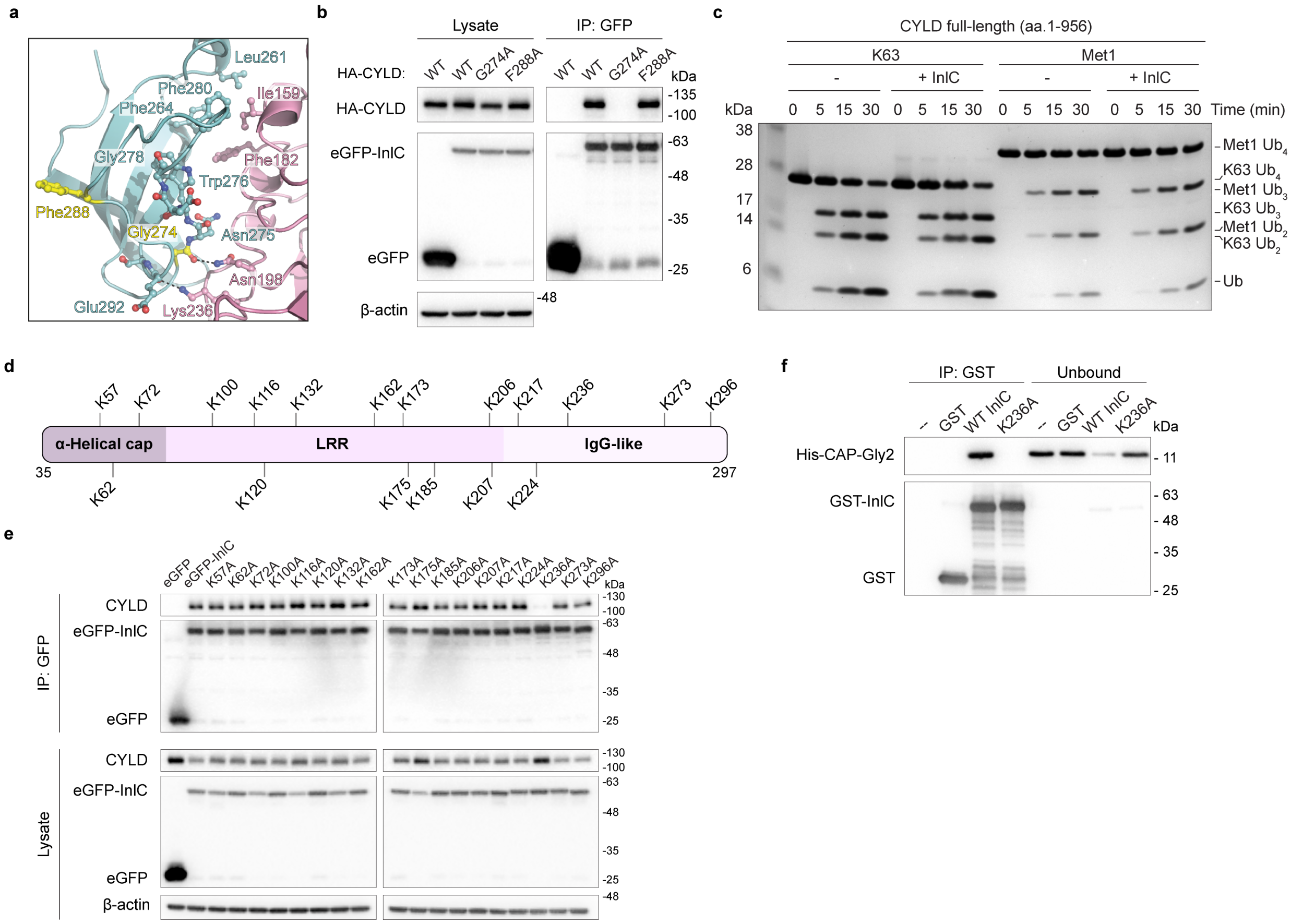

Supplementary Figure 3. InlC-CYLD binding is independent of ubiquitin modifications.

a, Alternative view of InlC-CYLD (PDB: 9TMK) depicting the location of CAP-Gly2 Phe288 (ubiquitin-binding) relative to Gly274 (InlC-binding). Selected residues are highlighted in yellow.

b, Co-immunoprecipitation of eGFP-InlC and HA-CYLD harboring CAP-Gly2 point mutations in *CYLD* KO HEK293T cells. Immunoblot is representative of *N*=3 biological replicates.

c, Qualitative CYLD deubiquitinase assays. Lys63-Ub4 or Met1-Ub4 were incubated with full-length CYLD in the absence or presence of recombinant InlC (aa. 35-297) over a time course of 30 minutes. Representative samples from *N*=2 biological replicates were resolved by SDS-PAGE and visualized using silver stain.

d, Schematic of the secreted form of InlC (aa. 35-297) and distribution of all (18) lysine residues.

e, Immunoprecipitation of eGFP-InlC lysine mutants, noted in (**d**), in HEK293T cells. Immunoblot is representative of *N*=3 biological replicates.

f, Recombinant protein binding assay using GST-InlC (wildtype or K236A) and His-CAP-Gly2. Immunoblot is representative of *N*=3 biological replicates. Source data are provided as a Source Data file.

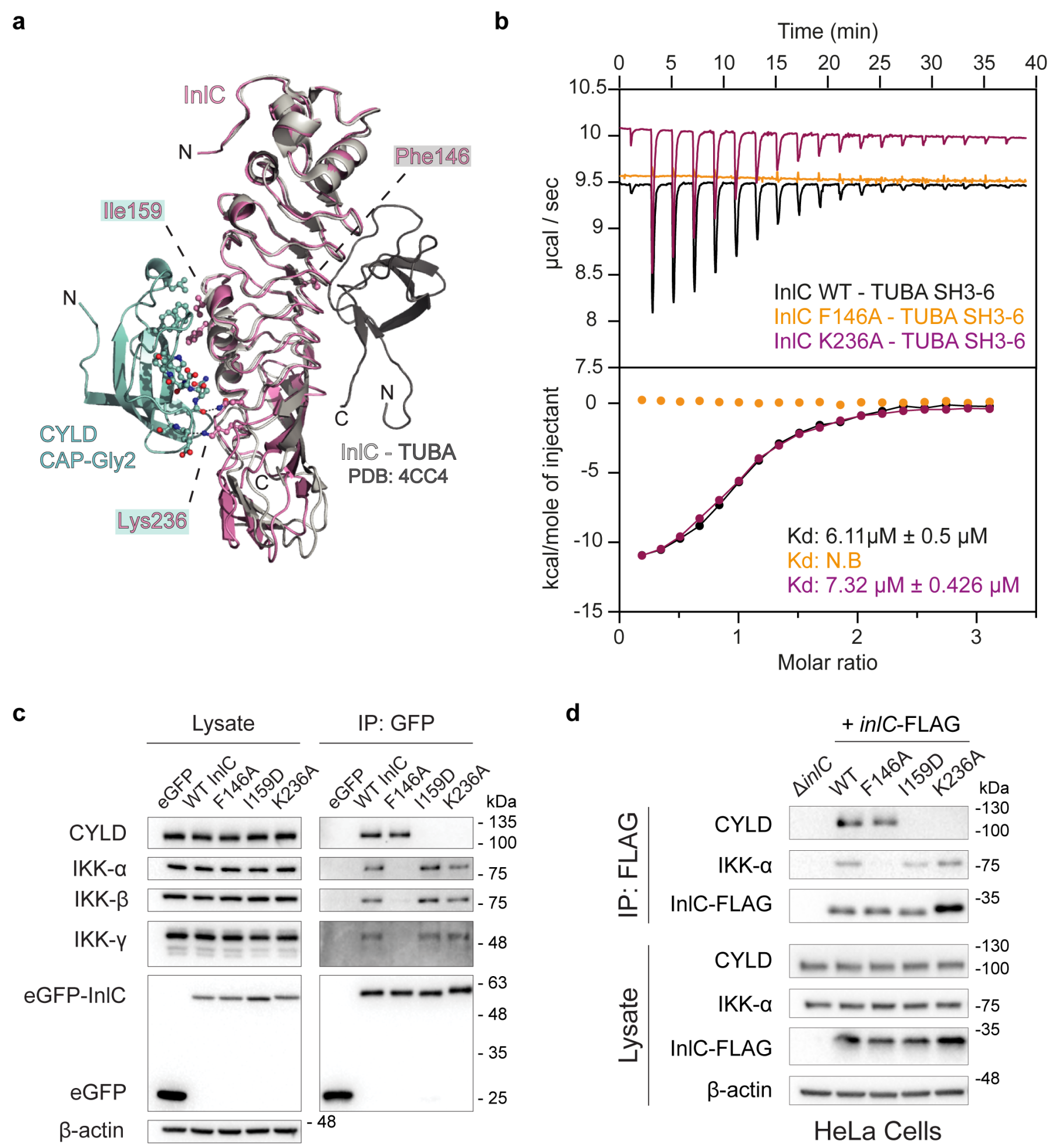

Supplementary Figure 4. The LRR domain of InlC has two distinct binding sites for its host protein targets.

a, Superimposed protein structures of InlC-CYLD (CAP-Gly2) (PDB: 9TMK) and InlC-TUBA (SH3-6) (PDB: 4CC4). Critical residues of InlC at each binding interface are noted.

b, ITC thermograms of TUBA (SH3-6) incubated with wildtype InlC (aa. 35-297) or mutants (F146A or K236A). Raw isotherms are shown (top) with integrated fits (bottom) for wildtype InlC and K236A. Data shown are representative of one technical replicate per condition.

c, Immunoprecipitation of eGFP-InlC mutants and the IKK complex in HEK293T cells. Immunoblot is representative of *N*=3 biological replicates.

d, Immunoprecipitation of InlC-FLAG from *Lm*-infected HeLa cells (20 h post-infection). Immunoblot is representative of *N*=3 biological replicates. Source data are provided as a Source Data file.

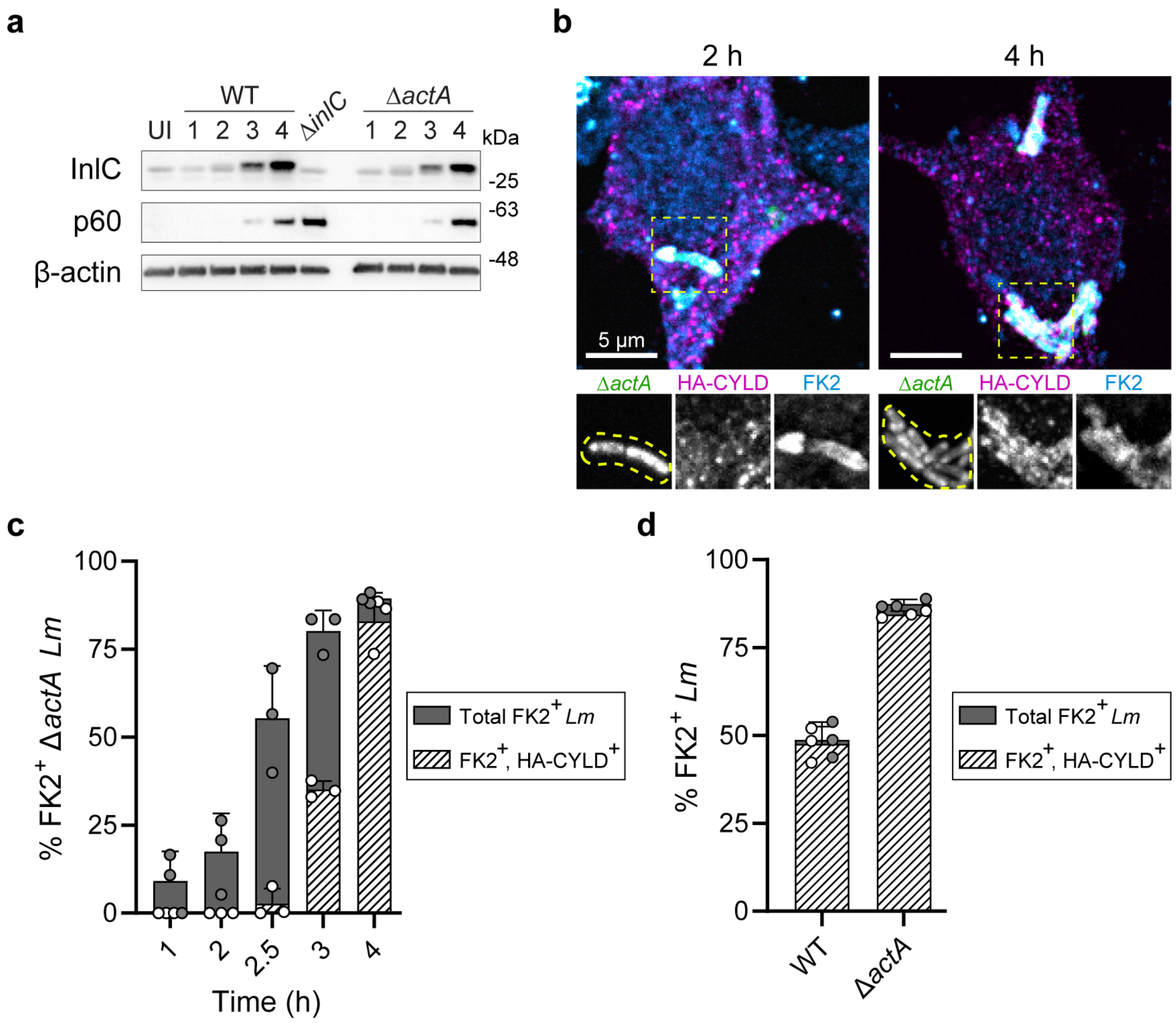

Supplementary Figure 5. InlC secretion coincides with CYLD recruitment to ubiquitin-positive bacteria.

a, Immunoblot of InlC secretion during *Lm* infection of RAW264.7 cells. Detection of *Lm* p60 confirms infection efficacy. Immunoblot is representative of *N*=3 biological replicates.

b-c, Time course of HA-CYLD recruitment to ubiquitin (FK2)-positive Δ*actA* bacteria during infection of RAW264.7 cells. Representative images of ubiquitin-positive *Lm* at 2- and 4-hours post-infection. Dashed lines highlight clusters of non-motile bacteria within the region of interest. Scale bar, 5 μm. Images are representative of *N*=3 biological replicates. **c**, Quantification of total FK2-positive and FK2-, CYLD-positive bacteria. Data are presented as mean values ± SD based on quantification of *N*=3 biological replicates. The number of transfected, infected RAW264.7 cells analyzed ranged from 15 to 20 per biological replicate.

d, Quantification of total FK2-positive and FK2-, CYLD-positive WT and Δ*actA* bacteria during infection of RAW264.7 cells (4 hours post-infection). Data are presented as mean values ± SD based on quantification of *N*=3 biological replicates. For WT and Δ*actA* conditions, the number of transfected, infected RAW264.7 cells analyzed was 15 and 16, respectively, per biological replicate. Source data are provided as a Source Data file.

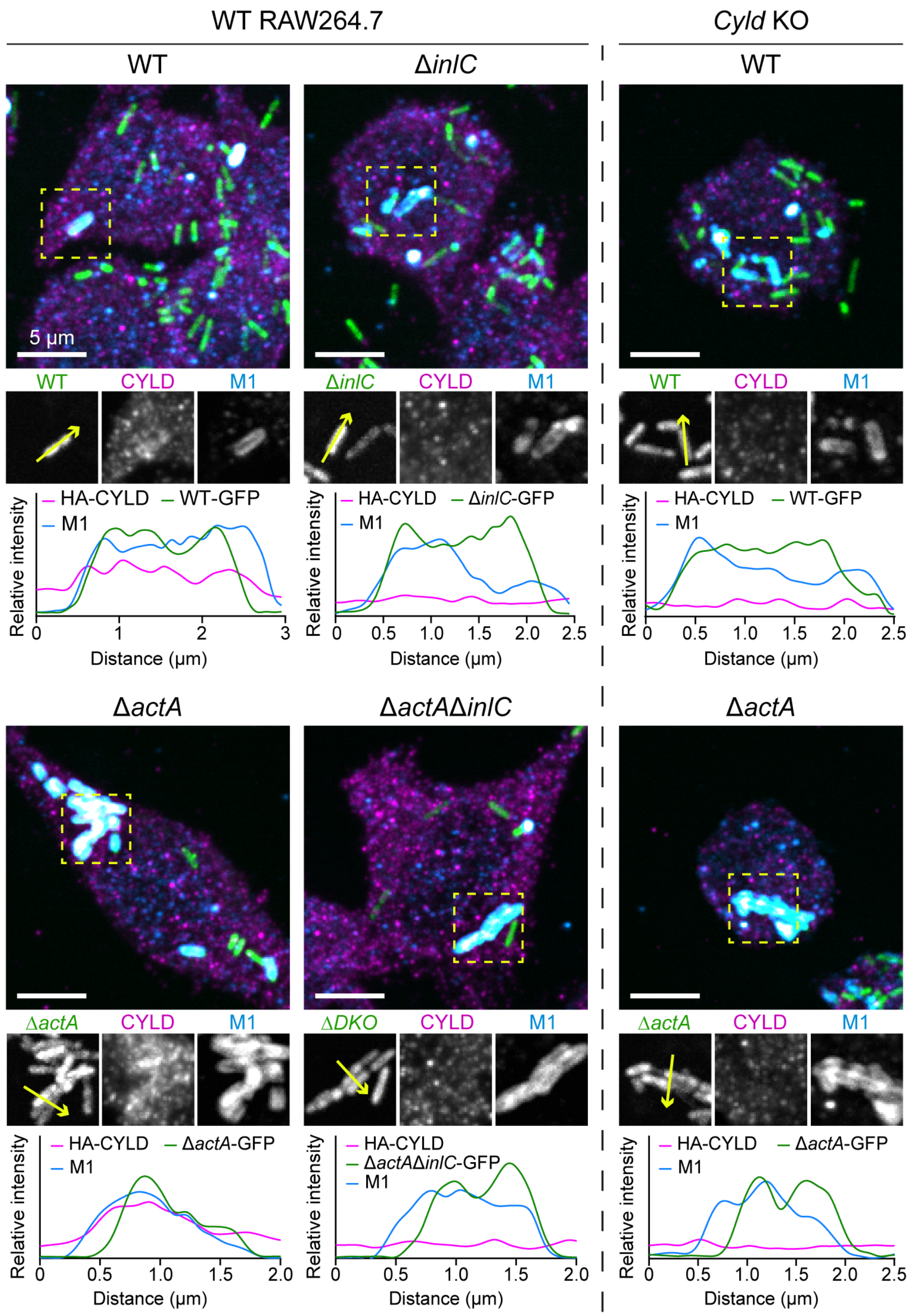

Supplementary Figure 6. Detection of endogenous CYLD at ubiquitin-positive *Lm*. Following 4 hours post-infection with either motile or non-motile *Lm*, WT or *Cyld* KO RAW264.7 cells were stained for endogenous CYLD and ubiquitin (Met1-linked). Met1-linked ubiquitin was stained due to same species conflict for antibodies against endogenous CYLD and FK2. Scale bar, 5 μm. Relative intensity profiles correspond to the arrow in the *Lm* channel. Images are representative of *N*=3 biological replicates. Source data are provided as a Source Data file.

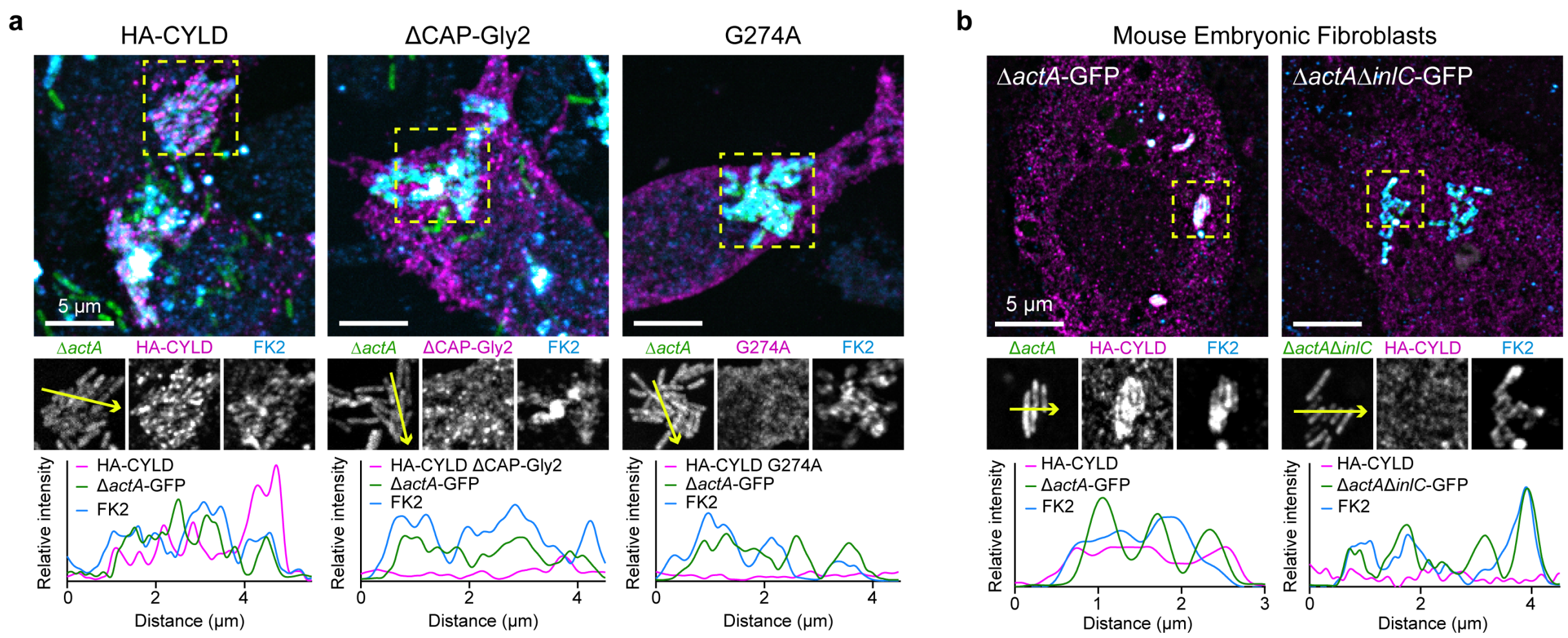

Supplementary Figure 7. Supportive evidence that InlC-CYLD binding is required for CYLD recruitment to ubiquitin-positive *Lm*.

a, HA-CYLD localization in Δ*actA*-infected *Cyld* KO RAW264.7 cells upon mutation of the CAP-Gly2 domain (4 hours post-infection). Scale bar, 5 µm. Relative intensity profiles correspond to the arrow in the *Lm* channel. Images are representative of *N*=3 biological replicates.

b, HA-CYLD localization in *Lm*-infected mouse embryonic fibroblasts (6 hours post-infection). Scale bar, 5 µm. Relative intensity profiles correspond to the arrow in the *Lm* channel. Images are representative of *N*=3 biological replicates. Source data are provided as a Source Data file.

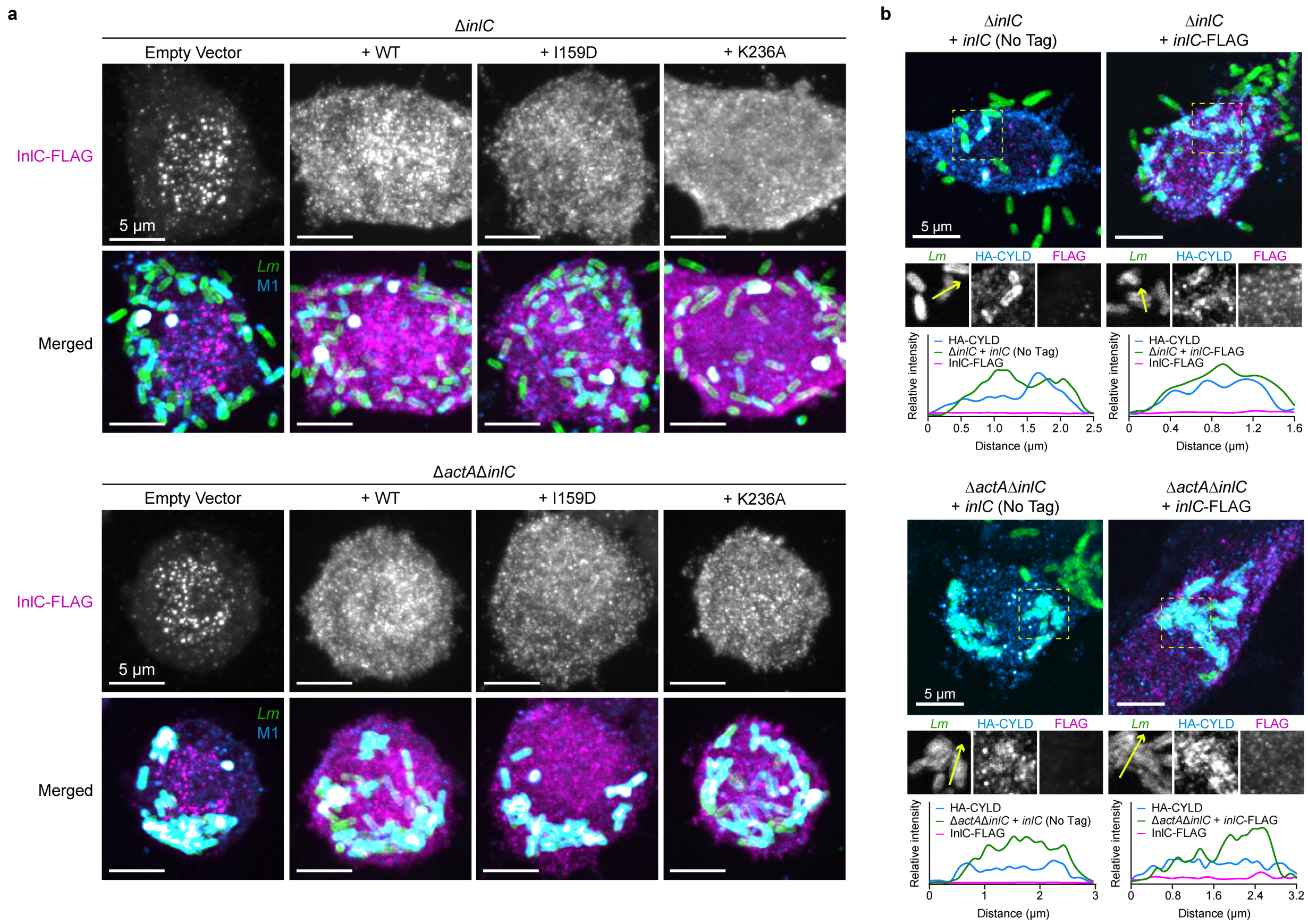

Supplementary Figure 8. InlC is not recruited to ubiquitin- or HA-CYLD-positive *Lm*.

a, InlC-FLAG localization in RAW264.7 cells infected with motile (Δ*inlC*) or non-motile (Δ*actA*Δ*inlC*) *Lm*, complemented with pLOV-*inlC*-FLAG expressing either wildtype *inlC* or single point mutants defective in binding to CYLD (I159D, K236A) (4 hours post-infection). Met1-linked ubiquitin was stained due to same species conflict for antibodies against FLAG and FK2. Scale bar, 5 µm. Images are representative of *N*=3 biological replicates.

b, HA-CYLD and InlC-FLAG localization in RAW264.7 cells infected with motile (Δ*inlC*) or non-motile (Δ*actA*Δ*inlC*) *Lm*, complemented with either wildtype *inlC* or *inlC*-FLAG (4 hours post-infection). Scale bar, 5 µm. Relative intensity profiles correspond to the arrow in the *Lm* channel. Images are representative of *N*=3 biological replicates. Source data are provided as a Source Data file.

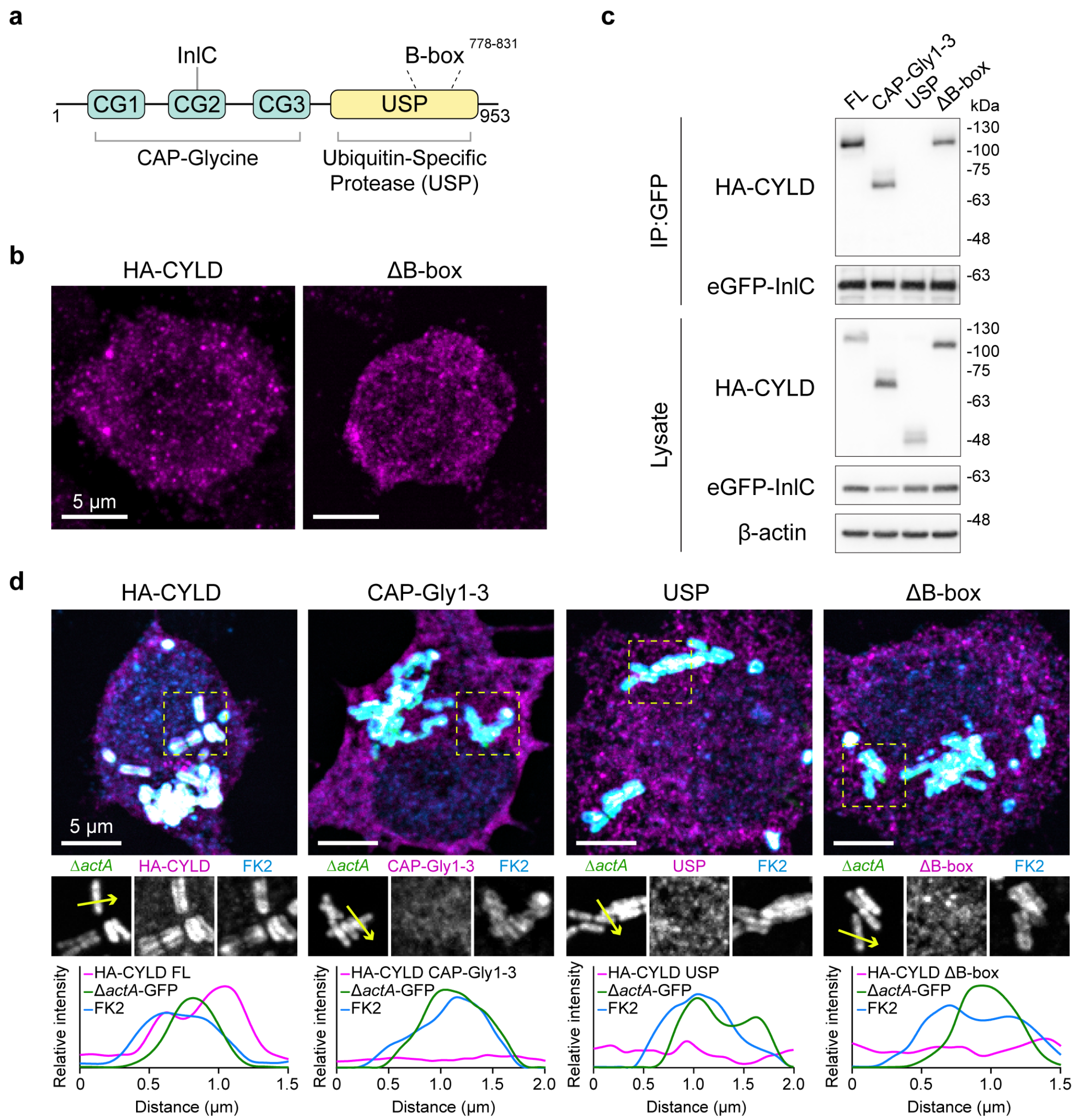

Supplementary Figure 9. The B-box module of CYLD is required for its recruitment to ubiquitin-positive *Lm*.

a, Schematic of HA-CYLD highlighting the InlC binding site (within CAP-Gly2 domain) and B-box module (within USP domain).

b, Localization of HA-CYLD and HA-CYLD$^{\Delta\text{B-box}}$ in uninfected in complemented *Cyld* KO RAW264.7 cells. Scale bar, 5 µm. Images are representative of *N*=3 biological replicates.

c, Co-immunoprecipitation of eGFP-InlC with full-length or truncated forms of HA-CYLD in *CYLD* KO HEK293T cells. Immunoblot is representative of *N*=3 biological replicates.

d, Localization of full-length or truncated forms of HA-CYLD in *Cyld* KO RAW264.7 cells infected with Δ*actA Lm* (4 hours post-infection). Scale bar, 5 µm. Relative intensity profiles correspond to the arrow in the *Lm* channel. Images are representative of three biological replicates. Source data are provided as a Source Data file.

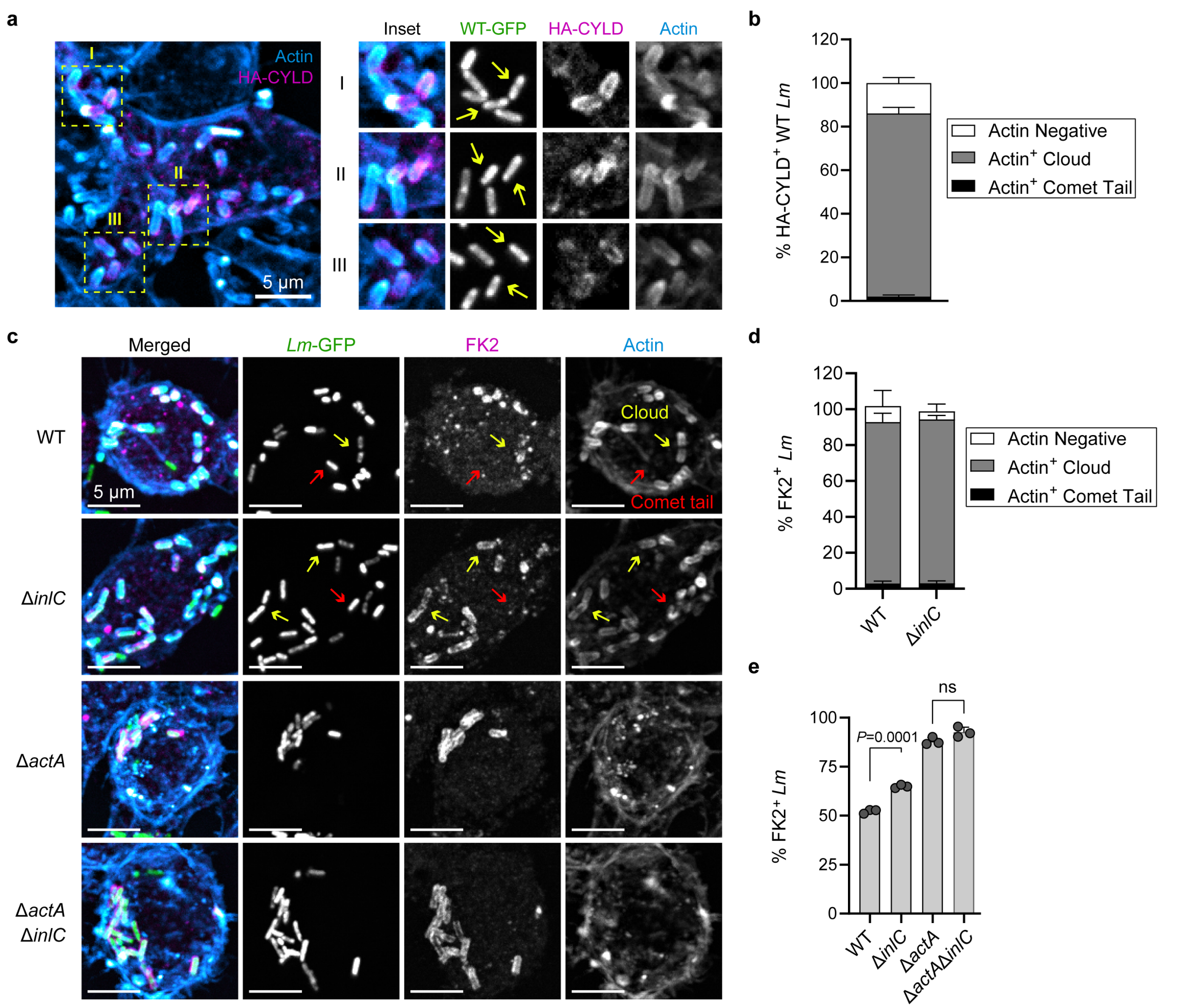

Supplementary Figure 10. HA-CYLD and ubiquitin localize with *Lm* prior to actin-based motility.

a-b, HA-CYLD and F-actin staining during infection of RAW264.7 cells with WT *Lm* (4 hours post-infection). Arrows highlight individual bacteria within regions of interest that are both HA-CYLD and F-actin-positive. Scale bar, 5 µm. Images are representative of *N*=3 biological replicates. **b,** Quantification of HA-CYLD-positive *Lm* that were either negative, or positive for F-actin in the form of an actin cloud or comet tail. Data are presented as mean values ± SD based on quantification of *N*=3 biological replicates. The number of transfected, infected RAW264.7 cells analyzed ranged from 15 to 19 per biological replicate.

c-e, Ubiquitin (FK2) and F-actin staining during infection of RAW264.7 cells with either motile or non-motile *Lm* (4 hours post-infection). Yellow and red arrows highlight individual bacteria displaying either an actin cloud or comet tail, respectively. Scale bar, 5 µm. Images are representative of *N*=3 biological replicates. **d**, Quantification of FK2-positive *Lm* that were either negative, or positive for F-actin in the form of an actin cloud or comet tail. Data are presented as

mean values ± SD based on quantification of *N*=3 biological replicates. For both WT and Δ*inlC*-infected conditions, the number of transfected, infected RAW264.7 cells analyzed ranged from 25 to 30 per biological replicate. **e**, Quantification of FK2-positive *Lm* per infected cell. Data are presented as mean values ± SD based on quantification of *N*=3 biological replicate. For each condition, the number of transfected, infected RAW264.7 cells analyzed per biological replicate ranged from 25 to 30 (WT, Δ*inlC*), 25 to 26 (Δ*actA*), and 25 (Δ*actA*Δ*inlC*). *P* values were determined using a two-tailed unpaired t-test (ns = not significant). Source data are provided as a Source Data file.

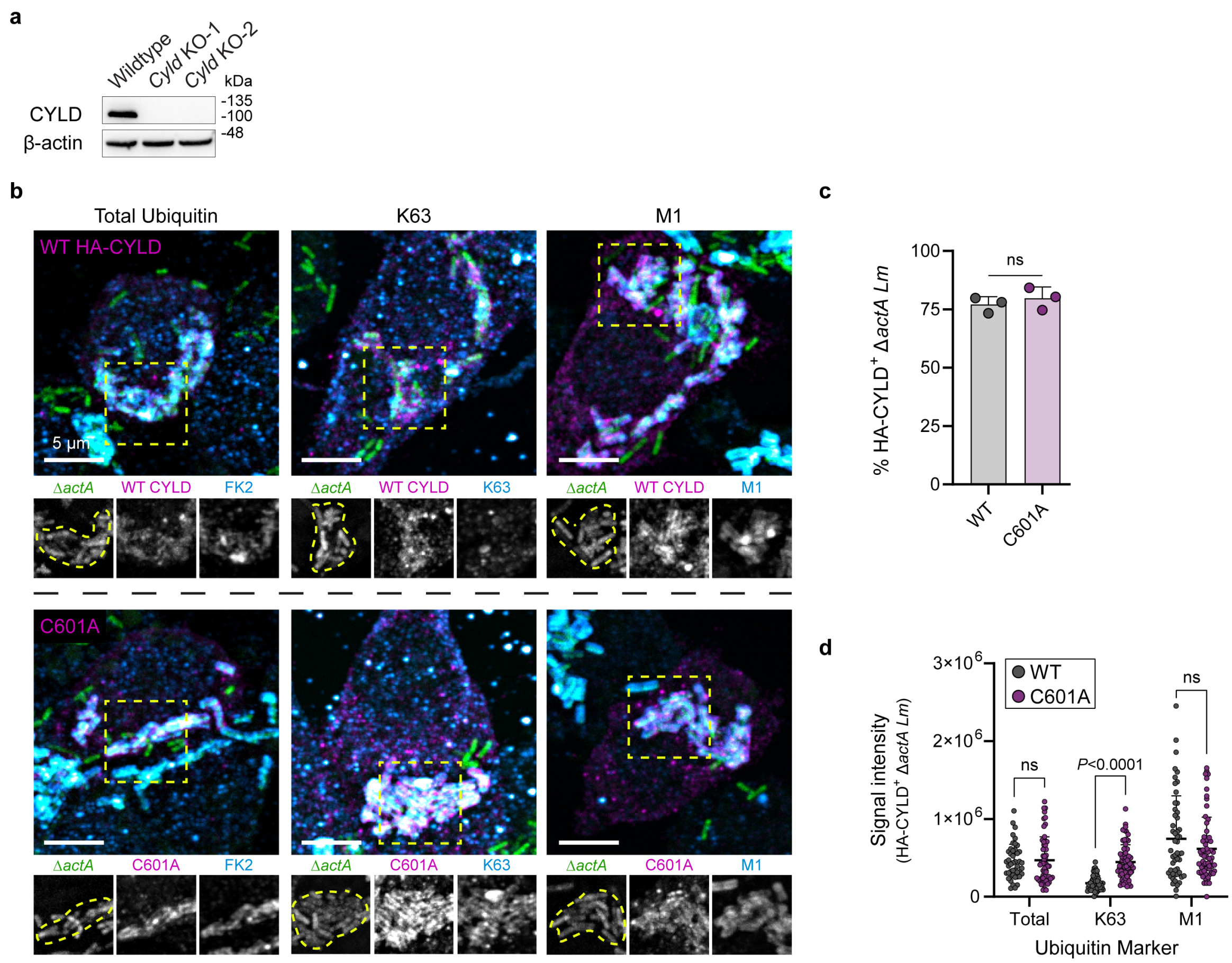

Supplementary Figure 11. CYLD deubiquitinates Lys63-linked ubiquitin at the bacterial surface.

a, Western blot validation of *Cyld* KO RAW264.7 cells. Immunoblot is representative of *N*=3 biological replicates.

b-d, Ubiquitin staining of Δ*actA* bacteria in *Cyld* KO RAW264.7 cells complemented with either wildtype or catalytically inactive (C601A) HA-CYLD (4 hours post-infection). Dashed lines highlight clusters of bacteria within the region of interest. Scale bar, 5 µm. Images are representative of *N*=3 biological replicates from one of two knockout clones. **c**, Recruitment frequency of wildtype and catalytically inactive (C601A) HA-CYLD to Δ*actA* bacteria. Data are presented as mean values ± SD based on quantification of *N*=3 biological replicates. For WT HA-CYLD, the number of transfected, infected RAW264.7 cells analyzed ranged from 15 to 17 per biological replicate. For HA-CYLD C601A, 16 transfected, infected RAW264.7 cells were analyzed per biological replicate. **d**, Quantification of ubiquitin intensity (sum) surrounding individual clusters of HA-CYLD-positive Δ*actA* bacteria. Data are presented as mean values ± SD based on quantification of individual clusters of non-motile bacteria across *N*=3 biological replicates. For WT HA-CYLD, the number of transfected, infected RAW264.7 cells analyzed per

biological replicate ranged from 13 to 17 (FK2), 15 to 25 (K63), and 14 to 19 (M1). For HA-CYLD C601A, the number of transfected, infected RAW264.7 cells analyzed per biological replicate ranged from 18 to 20 (FK2), 15 to 27 (K63), and 16 to 26 (M1). *P* values were determined using a two-tailed unpaired t-test (ns = not significant). Source data are provided as a Source Data file.

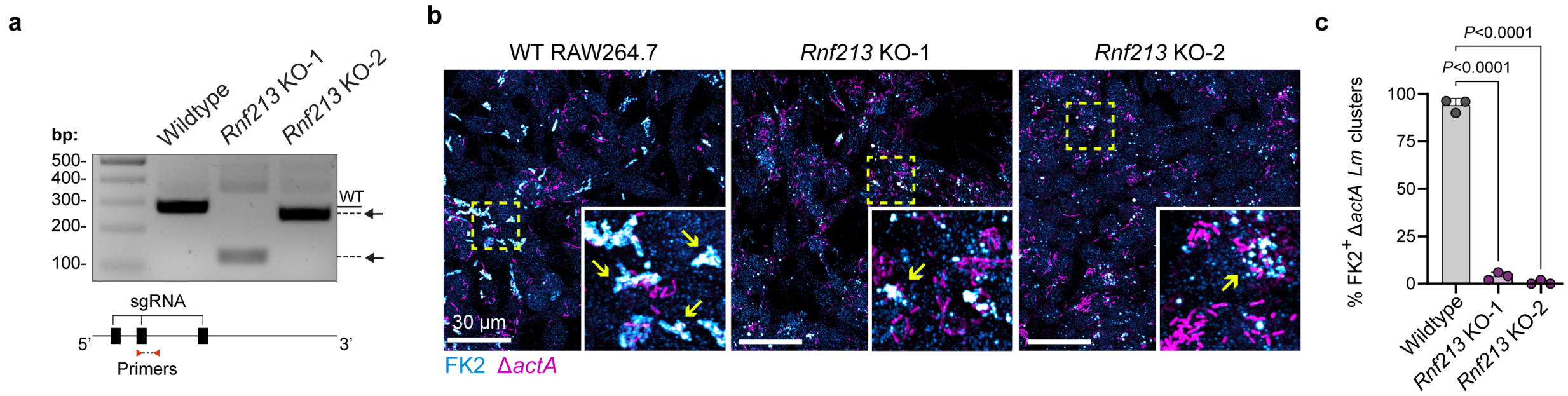

Supplementary Figure 12. Validation of *Rnf213* KO RAW264.7 cells.

a, Genomic validation of *Rnf213* disruption in RAW264.7 cells. Black bars denote exons targeted by three separate sgRNAs. Arrows indicate a weight shift in PCR product relative to WT cells. Findings are representative of N=2 biological replicates.

b-c, Total ubiquitin (FK2) staining of Δ*actA* bacteria in *Rnf213* KO RAW264.7 cells (4 hours post-infection). Scale bar, 30 µm. Arrows highlight clusters of bacteria considered to be ubiquitin-positive. **c**, Quantification of total ubiquitin-positive Δ*actA* clusters. Data are presented as mean values ± SD based on quantification of individual clusters of non-motile bacteria across N=3 biological replicates. For each condition, the number of transfected, infected RAW264.7 cells analyzed per biological replicate ranged from 50 to 53 (wildtype), 49 to 50 (*Rnf213* KO-1), and 35 to 50 (*Rnf213* KO-2). P values were determined using a one-way ANOVA with Dunnett's post-hoc test. Source data are provided as a Source Data file.

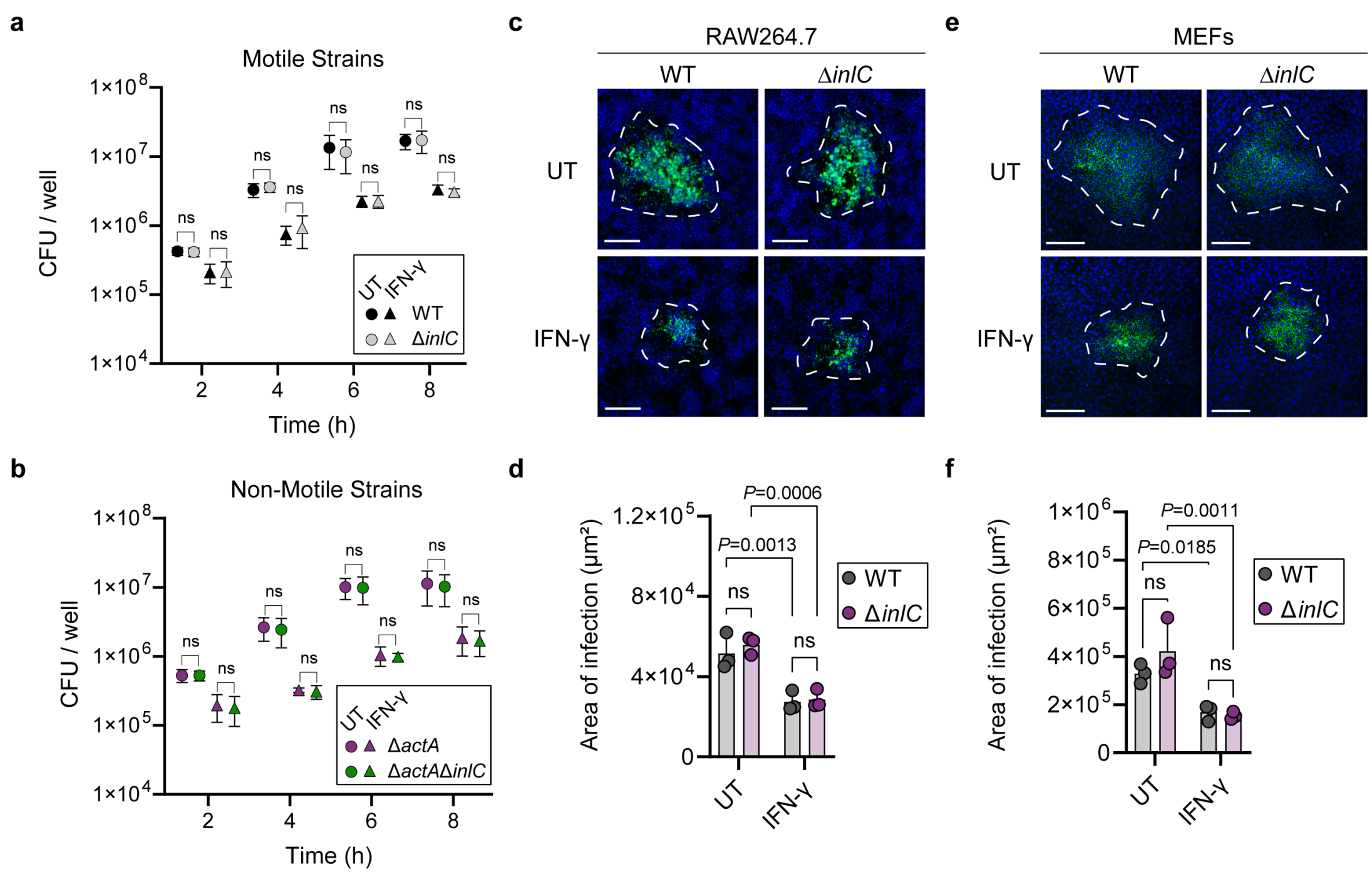

Supplementary Figure 13. Loss of InlC does not impact bacterial growth in macrophages, or *Lm* cell-to-cell spread in RAW264.7 cells and MEFs.

a-b, Bacterial growth of motile (**a**) or non-motile (**b**) *Lm* strains in untreated or IFN-γ-primed RAW264.7 cells (2-8 hours post-infection). All colony forming units (CFU) are displayed as mean ± SD enumerated across *N* =3 biological replicates. *P* values were determined using a two-way ANOVA (ns = not significant).

c-d, *Lm* cell-to-cell spread in untreated or IFN-γ-primed RAW264.7 cells (20 hours post-infection). Dotted lines delineate the edge of the infection area. Scale bar, 100 µm. Images are representative of *N*=3 biological replicates. **d**, Quantification of the area of infection (µm) displayed as mean ± SD across *N*=3 biological replicates. For both WT and Δ*inlC* conditions, 30 infection foci were quantified per biological replicate. *P* values were determined using a two-way ANOVA with Tukey's post-hoc test (ns = not significant).

e-f, *Lm* cell-to-cell spread in untreated or IFN-γ-primed MEFs (20 hours post-infection). Dotted lines delineate the edge of the infection area. Scale bar, 200 µm. Images are representative of *N*=3 biological replicates. **f**, Quantification of the area of infection (µm) displayed as mean ± SD across *N*=3 biological replicates. For the WT condition, the number of infection foci analyzed per biological replicate ranged from 15 to 26. For the Δ*inlC* condition, the number of infection foci analyzed per biological replicate ranged from 15 to 25. *P* values were determined using a two-way ANOVA with Tukey's post-hoc test (ns = not significant). Source data are provided as a Source Data file.

Supplementary Table 1. Data collection statistics for crystallization of InlC and CAP-Gly2. Numbers in brackets are for the highest resolution bin. Data was collected from a single crystal.

	InlC 35-297–CYLD 226-313
**Data collection**	
Beamline	Diamond I04
Space group	$P\,2_1 2_1 2_1$
a, *b*, *c* (Å)	58.83, 70.01, 97.72
α, β, γ (°)	90.00, 90.00, 90.00
Wavelength	0.95374
Resolution (Å)	50.4 – 1.80 (1.84 – 1.80)
R_{merge}	8.3 (131)
$< I / \sigma I >$	15.5 (1.6)
CC(1/2)	1.00 (0.78)
Completeness (%)	100.0 (100.0)
Redundancy	13.3 (13.1)
**Refinement**	
Resolution (Å)	50.40 – 1.80
No. reflections (test set)	36,260 (1,885)
R_{work} / R_{free}	20.4/23.8
No. atoms	
Protein	5,406
Water	305
Ligand	21
B-factors	
Wilson B	29.3
Protein	45.4
Water	39.2
Ligand	35.9
R.m.s deviations	
Bond lengths (Å)	0.004
Bond angles (°)	0.729
Ramachandran statistics (outliers, allowed, favoured)	0.0, 4.2, 95.8
