# Supplementary material for: Listeria promotes systemic infection by targeting the host deubiquitinase CYLD
Source: Nat Commun. 2026 Aug 31;17:10351. doi: 10.1038/s41467-026-77063-5 (PMC13623971; doi:10.1038/s41467-026-77063-5)
Supplement: Supplementary file 2 [file 41467_2026_77063_MOESM2_ESM.pdf]

**Description of Additional Supplementary Files:**

**Supplementary Data 1. Summary of BioID results for InlC-BirA* compared to BirA* control, performed in HEK293 Flp-In T-REx cells.** Each of the two biological replicates was analyzed using two technical replicates. Data were analyzed using the Trans Proteomic Pipeline56, via the ProHits software suite57. Proteins identified with a Protein Prophet cut-off of 0.9 and at least two unique peptides were analyzed with the SAINT express algorithm (v.3.6.1). Twelve control runs (consisting of Flag BirA*only) were collapsed to the two highest spectral counts for each prey, and the SAINT score cut-off value was set to a Bayesian false discovery rate (BFDR) < 0.01 (1% FDR). Listed are spectral counts (Spec) and summed spectra (SpecSum) for each protein identified, with one sheet ("Unfiltered") showing all detected proteins and a second sheet ("Filtered") showing proteins filtered for a BFDR < 0.01.

**Supplementary Data 2. List of experimental models, cell lines and strains used in this study.**

**Supplementary Data 3. List of plasmids, primers and cloning methods used in this study.**

**Supplementary Data 4. List of antibodies used in this study.**
