# Supplementary material for: Listeria promotes systemic infection by targeting the host deubiquitinase CYLD
Source: Nat Commun. 2026 Aug 31;17:10351. doi: 10.1038/s41467-026-77063-5 (PMC13623971; doi:10.1038/s41467-026-77063-5)
Supplement: Supplementary file 3 [file 41467_2026_77063_MOESM7_ESM.pdf]

nature portfolio

Corresponding author(s): Dr. John H. Brumell

Last updated by author(s): july 22, 2026



# Reporting Summary

Nature Portfolio wishes to improve the reproducibility of the work that we publish. This form provides structure for consistency and transparency in reporting. For further information on Nature Portfolio policies, see our Editorial Policies and the Editorial Policy Checklist.

## Statistics

For all statistical analyses, confirm that the following items are present in the figure legend, table legend, main text, or Methods section.

| n/a | Confirmed | |
|---|---|---|
| ☐ | ☒ | The exact sample size (*n*) for each experimental group/condition, given as a discrete number and unit of measurement |
| ☐ | ☒ | A statement on whether measurements were taken from distinct samples or whether the same sample was measured repeatedly |
| ☐ | ☒ | The statistical test(s) used AND whether they are one- or two-sided<br>*Only common tests should be described solely by name; describe more complex techniques in the Methods section.* |
| ☒ | ☐ | A description of all covariates tested |
| ☒ | ☐ | A description of any assumptions or corrections, such as tests of normality and adjustment for multiple comparisons |
| ☐ | ☒ | A full description of the statistical parameters including central tendency (e.g. means) or other basic estimates (e.g. regression coefficient) AND variation (e.g. standard deviation) or associated estimates of uncertainty (e.g. confidence intervals) |
| ☐ | ☒ | For null hypothesis testing, the test statistic (e.g. *F*, *t*, *r*) with confidence intervals, effect sizes, degrees of freedom and *P* value noted<br>*Give P values as exact values whenever suitable.* |
| ☒ | ☐ | For Bayesian analysis, information on the choice of priors and Markov chain Monte Carlo settings |
| ☒ | ☐ | For hierarchical and complex designs, identification of the appropriate level for tests and full reporting of outcomes |
| ☒ | ☐ | Estimates of effect sizes (e.g. Cohen's *d*, Pearson's *r*), indicating how they were calculated |

*Our web collection on statistics for biologists contains articles on many of the points above.*

## Software and code

Policy information about availability of computer code

**Data collection**

The software/equipment used to collect data in this study are as follows:

For BioID-MS, HPLC was performed using a Proxeon EASY-nLC 1000 pump coupled with a hybrid LTQ-Orbitrap Velos mass spectrometer (Thermo). All data collected can be found in Supplementary Data 1.

Isothermal titration calorimetry experiments were performed using an Auto-iTC200 instrument (Malvern Instruments, Malvern, UK).

Diffraction data of InlC-CAP-Gly2 crystals were collected at Diamond Light source beamline I04. The wavelength and temperature for data collection were 0.95374 Å and 77 K, respectively.

Confocal images were captured on a spinning disk system (Quorum Technologies) comprised of a DMI6000 B inverted fluorescence microscope (Leica) equipped with a Hamamatsu CMOS FL-400 camera and Volocity v6.3.1 software.

Immunoblots were imaged on a Gel Doc XR system equipped with Image Lab software (Bio-Rad, v6.1).

**Data analysis**

The software used to analyze data in this study are as follows:

For protein identification of InlC-BirA* BioID, Thermo.RAW files were converted to the .mzXML format using Proteowizard54, then searched using iProphet55 against the human (Human RefSeq v.45) database. X!Tandem search parameters were: 15 ppm parent mass error; 0.4 Da fragment mass error; complete modifications, none; cysteine modifications, none; potential modifications, +16@M and W, +32@M and W,

+42@N-terminus, +1@N and Q. Each of the two biological replicates was analyzed using two technical replicates. Data were analyzed using the Trans Proteomic Pipeline56, via the ProHits software suite57.

For isothermal titration calorimetry experiments, the changes in heat release were integrated over the entire titration and fitted to a single-site binding model using the PEAQ Analysis software (Malvern Instruments) and graphs were plotted in Prism (GraphPad, v9).

Diffraction images of InlC-CAP-Gly2 crystals were processed using DIALS59 and manually scaled using AIMLESS60 as part of the CCP4 software package61. The structure of InlC bound to CAP-Gly2 was determined by molecular replacement using PHASER62 placing CAP-Gly2 and InlC (PDB IDs: 1WHM and 1XEU respectively). Iterative rounds of model building and refinement were performed with COOT63 and PHENIX64, respectively. Data collection and refinement statistics can be found in Supplementary Table 1.

All confocal images were analyzed (e.g. manual counts across planes, fluorescence intensity, dilation, deconvolution, area of infection) using Volocity v6.3.1 software. Representative images were assembled using ImageJ and Adobe Illustrator. All line graphs display the spline curves of relative fluorescence values, displayed as arbitrary units, which were generated in Prism.

Representative immunoblots were processed using Image Lab software (Bio-Rad, v6.1).

All schematics were conceptualized and generated by D.A.A. using Adobe Illustrator 2025 (v29).

For manuscripts utilizing custom algorithms or software that are central to the research but not yet described in published literature, software must be made available to editors and reviewers. We strongly encourage code deposition in a community repository (e.g. GitHub). See the Nature Portfolio guidelines for submitting code & software for further information.

## Data

Policy information about availability of data

All manuscripts must include a data availability statement. This statement should provide the following information, where applicable:

- Accession codes, unique identifiers, or web links for publicly available datasets
- A description of any restrictions on data availability
- For clinical datasets or third party data, please ensure that the statement adheres to our policy

The raw mass spectrometry data for the InlC-BirA* BioID generated in this study has been deposited to the MassIVE public repository (https://massive.ucsd.edu) under accession code #MSV000100210 [http://doi.org/10.25345/C5CN6ZC5P]. The protein structure of InlC-CYLD CAP-Gly2 has been deposited to the Protein Data Bank (PDB: 9TMK) [http://doi.org/10.2210/pdb9TMK/pdb]. All primers and cloning methods used to generate the constructs used in this study are provided in the Supplementary Data. Source data are provided with this paper. Requests for resources and reagents should be directed to corresponding author.

## Research involving human participants, their data, or biological material

Policy information about studies with human participants or human data. See also policy information about sex, gender (identity/presentation), and sexual orientation and race, ethnicity and racism.

| | |
|---|---|
| Reporting on sex and gender | Human participants, their data, or biological material were not used in this research study. |
| Reporting on race, ethnicity, or other socially relevant groupings | Human participants, their data, or biological material were not used in this research study. |
| Population characteristics | Human participants, their data, or biological material were not used in this research study. |
| Recruitment | Human participants, their data, or biological material were not used in this research study. |
| Ethics oversight | Human participants, their data, or biological material were not used in this research study. |

Note that full information on the approval of the study protocol must also be provided in the manuscript.

# Field-specific reporting

Please select the one below that is the best fit for your research. If you are not sure, read the appropriate sections before making your selection.

- [x] Life sciences
- [ ] Behavioural & social sciences
- [ ] Ecological, evolutionary & environmental sciences

For a reference copy of the document with all sections, see nature.com/documents/nr-reporting-summary-flat.pdf

# Life sciences study design

All studies must disclose on these points even when the disclosure is negative.

| | |
|---|---|
| Sample size | For in vitro studies, no sample size calculation was performed. Sample sizes were chosen based common practice in the field and the variability within an experiment (Tan et al., 2018; Tan et al., 2021).<br><br>For in vivo experiments, a power analysis was performed in Prism (GraphPad) to determine the optimal sample size (PMID: 24250214). |

| | |
|---|---|
| Data exclusions | No data was excluded from analysis. |
| Replication | All attempts at replication for all experiments described in this study were successful.<br><br>All immunoblots and immunofluoresence images in this manuscript are representative of three, independent biological replicates performed on separate days.<br><br>Qualitative deubiquitinase assays are representative of findings from 2 biological replicates.<br><br>All ITC experiments are representative of findings from 1-3 technical replicates from 1 biological replicate per experiment.<br><br>In vivo work is representative of two, independent biological replicates performed on separate days. |
| Randomization | The experiments were not randomized. The experiments investigated specific cell types and treatments and required precise control over the experimental conditions, aspects that are not conditions amenable to randomization.<br><br>For in vivo experiments, wildtype mice were sex-, age- and weight-matched to ensure reproducibility and minimize variability. |
| Blinding | Blinding was not relevant to in vitro experiments since cells were treated with more than one condition. Blinding does not affect data outcome of in vivo infections as every mice is given the same treatment. |

# Reporting for specific materials, systems and methods

We require information from authors about some types of materials, experimental systems and methods used in many studies. Here, indicate whether each material, system or method listed is relevant to your study. If you are not sure if a list item applies to your research, read the appropriate section before selecting a response.

## Materials & experimental systems

| n/a | Involved in the study | |
|---|---|---|
| ☐ | ☒ | Antibodies |
| ☐ | ☒ | Eukaryotic cell lines |
| ☒ | ☐ | Palaeontology and archaeology |
| ☐ | ☒ | Animals and other organisms |
| ☒ | ☐ | Clinical data |
| ☒ | ☐ | Dual use research of concern |
| ☒ | ☐ | Plants |

## Methods

| n/a | Involved in the study | |
|---|---|---|
| ☒ | ☐ | ChIP-seq |
| ☒ | ☐ | Flow cytometry |
| ☒ | ☐ | MRI-based neuroimaging |

## Antibodies

| | |
|---|---|
| Antibodies used | All antibodies used in this study can be found in Supplementary Data 4.<br><br>Primary antibodies used for immunofluorescence with corresponding dilutions:<br>-Rabbit anti-Lm (BD; #223021) (1:500)<br>-Rabbit anti-HA-Tag (Cell Signaling; #C29F4) (1:200)<br>-Mouse anti-HA-Tag (Cell Signaling; #2367) (1:200)<br>-Mouse anti-FLAG-Tag (Cell Signaling; #8146) (1:200)<br>-Mouse anti-CYLD (Santa-Cruz; #sc-74435, C-term.) (1:100)<br>-Mouse anti-ubiquitin (Sigma-Aldrich; #04-263, FK2) (1:200)<br>-Rabbit anti-Lys63 ubiquitin (Sigma-Aldrich; #05-1308, Apu3) (1:100)<br>-Rabbit monoclonal anti-linear ubiquitin (Sigma-Aldrich; #ZRB2114, 1E3) (1:200)<br><br>Secondary antibodies, fluorescent probes and dyes used for immunofluorescence with corresponding dilutions<br>-Alexa Fluor-conjugated goat anti-mouse (Thermo; #A-11029, #A-11031, A-21236) (1:200)<br>-Alexa Fluor-conjugated goat anti-rabbit (Thermo; #A-11034, A-11036, A-21245) (1:200)<br>-Zenon rabbit IgG labelling kit (Thermo; #Z25306, #Z25308) (1:200)<br>-Acti-stain 670 (Cytoskeleton Inc; #PHDN1) (1:200)}<br>-4′,6-diamidino-2-phenylindole (DAPI) (Thermo; #D1306) (0.25 μg/mL)<br>Primary antibodies for western blot (1:1,000 dilution unless otherwise noted):<br>-Mouse anti-GFP (Santa Cruz; #sc-9996) (1:10,000)<br>-Mouse anti-CYLD (Santa-Cruz; #sc-74435, C-term.)<br>-Rabbit anti-CYLD (Cell Signaling; #8462, N-term.)<br>-Rabbit anti-FLAG (Cell Signaling; #14793)<br>-Rabbit anti-SPATA2 (Bethyl; #A302-494A)<br>-Rabbit anti-IKK-α (Cell Signaling; #61294) |

-Rabbit anti-IKK-β (Cell Signaling; #8943)
-Mouse anti-IKKγ (Cell Signaling; #2695)
-Mouse anti-p60 (Novus Biologicals; # NBP2-80120)
-Rabbit anti-InlC (Gift from Dr. Keith Ireton; PMID: 19767742)
-Mouse anti-β-actin (Sigma-Aldrich; A5441) (1:10,000).
Secondary antibodies for western blot (1:5,000 dilution):
-HRP-conjugated goat anti-rabbit IgG (Jackson ImmunoResearch; #111-035-144)
-HRP-conjugated goat anti-mouse IgG (Jackson ImmunoResearch; #115-035-146)

Validation

All antibodies used in this study were validated by the manufacturers. Comprehensive validation statements, alongside relevant details such as species reactivity, applications, and supporting citations, are accessible through the provided website links.

Essential information pertaining to the antibodies employed in this study are outlined below:
-Rabbit anti-Lm (BD; #223021)
Species reactivity: Listeria monocytogenes (Poly Types 1 & 4)
Application (in this study): immunofluoresence
Validation statements: https://www.bd.com/en-us/products-and-solutions/products/product-page.223021#overview

-Rabbit anti-HA-Tag (Cell Signaling; #C29F4)
Species reactivity: All
Application (in this study): immunofluorescence and western blotting
Validation statements: https://www.cellsignal.com/products/primary-antibodies/ha-tag-c29f4-rabbit-monoclonal-antibody/3724

-Mouse anti-HA-Tag (Cell Signaling; #2367)
Species reactivity: All
Application (in this study): immunofluorescence and western blotting
Validation statements: https://www.cellsignal.com/products/primary-antibodies/ha-tag-6e2-mouse-monoclonal-antibody/2367

-Mouse anti-FLAG-Tag (Cell Signaling; #8146)
Species reactivity: All
Application (in this study): immunofluorescence and western blot
Validation statements: https://www.cellsignal.com/products/primary-antibodies/dykddddk-tag-9a3-mouse-monoclonal-antibody-binds-to-same-epitope-as-sigma-aldrich-anti-flag-m2-antibody/8146

-Mouse anti-CYLD (Santa-Cruz; #sc-74435, C-term.)
Species reactivity: mouse, human
Application (in this study): immunofluorescence and western blot
Validation statements: https://www.scbt.com/p/cylindromatosis-1-antibody-e-10?srsltid=AfmBOornj5x_BvUDqwmfADnd7u7XlgLlPMCbY3vI-_3XE-pp7QdM9wK4

-Mouse anti-ubiquitin (Sigma-Aldrich; #04-263, FK2)
Species reactivity: All
Application (in this study): immunofluorescence
Validation statements: https://www.sigmaaldrich.com/CA/en/product/mm/04263?srsltid=AfmBOorXoBOIcUF56Op1jEEO67jY8besONdqaTir2pfIFiWyYfo64HS9

-Rabbit anti-Lys63 ubiquitin (Sigma-Aldrich; #05-1308, Apu3)
Species reactivity: mouse, human
Application (in this study): immunofluorescence
Validation statements: https://www.sigmaaldrich.com/CA/en/product/mm/051308

-Rabbit monoclonal anti-linear ubiquitin (Sigma-Aldrich; #ZRB2114, 1E3)
Species reactivity: human, mouse (this study)
Application (in this study): immunofluorescence
Validation statements: https://www.sigmaaldrich.com/CA/en/product/sigma/zrb2114

-Alexa Fluor-conjugated goat anti-mouse (Thermo; #A-11029, #A-11031, A-21236) (1:200)
Species reactivity: mouse IgG
Application (in this study): immunofluoresence
Validation statements: https://www.thermofisher.com/antibody/secondary/target/goat%20anti-mouse%20igg

-Alexa Fluor-conjugated goat anti-rabbit (Thermo; #A-11034, A-11036, A-21245) (1:200)
Species reactivity: rabbit IgG
Application (in this study): immunofluoresence
Validation statements: https://www.thermofisher.com/antibody/secondary/target/goat%20anti-rabbit%20igg

-Zenon rabbit IgG labelling kit (Thermo; #Z25306, #Z25308)
Species reactivity: rabbit IgG
Application (in this study): immunofluoresence

Validation statements: https://www.thermofisher.com/order/catalog/product/Z25306

-Mouse anti-GFP (Santa Cruz; #sc-9996)
Species reactivity: All
Application (in this study): immunofluorescence
Validation statements: https://www.scbt.com/p/gfp-antibody-b-2?srsltid=AfmBOoqAw438uwXaPIr2T1P7hZDU-BLagFJyQf_p3WmPJwJdwoAf43aJ

-Rabbit anti-CYLD (Cell Signaling; #8462, N-term.)
Species reactivity: mouse, human
Application (in this study): western blot
Validation statements: https://www.cellsignal.com/products/primary-antibodies/cyld-d1a10-rabbit-monoclonal-antibody/8462

-Rabbit anti-FLAG (Cell Signaling; #14793)
Species reactivity: All
Application (in this study): western blot
Validation statements: https://www.cellsignal.com/products/primary-antibodies/dykddddk-tag-d6w5b-rabbit-monoclonal-antibody-binds-to-same-epitope-as-sigma-aldrich-anti-flag-m2-antibody/14793

-Rabbit anti-SPATA2 (Bethyl; #A302-494A)
Species reactivity: human
Application (in this study): western blot
Validation statements: https://www.thermofisher.com/antibody/product/SPATA2-Antibody-Polyclonal/A302-493A

-Rabbit anti-IKK-α (Cell Signaling; #61294)
Species reactivity: human
Application (in this study): western blot
Validation statements: https://www.cellsignal.com/products/primary-antibodies/ikk-alpha-d3w6n-rabbit-monoclonal-antibody/61294

-Rabbit anti-IKK-β (Cell Signaling; #8943)
Species reactivity: human
Application (in this study): western blot
Validation statements: https://www.cellsignal.com/products/primary-antibodies/ikk-beta-d30c6-rabbit-monoclonal-antibody/8943

-Mouse anti-IKKγ (Cell Signaling; #2695)
Species reactivity: human
Application (in this study): western blot
Validation statements: https://www.cellsignal.com/products/primary-antibodies/ikk-gamma-da10-12-mouse-monoclonal-antibody/2695

-Mouse anti-p60 (Novus Biologicals; # NBP2-80120)
Species reactivity: Listeria monocytogenes
Application (in this study): western blot
Validation statements: https://www.novusbio.com/products/listeria-monocytogenes-p60-antibody-p6007_nbp2-80120?srsltid=AfmBOoqmh3LN1LjOruVcfP3o7VVv-gXvYvF8aQb-8cwWI4zD7IrK8fub

-Rabbit anti-InlC (Gift from Dr. Keith Ireton; PMID: 19767742)
Species reactivity: Listeria monocytogenes
Application (in this study): western blot
Validation statements: PMID-19767742

-Mouse anti-β-actin (Sigma-Aldrich; A5441)
Species reactivity: human, mouse
Application (in this study): western blot
Validation statements: https://www.sigmaaldrich.com/CA/en/product/sigma/a5441?srsltid=AfmBOopFtYcOKwdeeL2-U_H8bsGKlrcxkkCUEtWNOWUmVpb39iWAQzB_

-HRP-conjugated goat anti-rabbit IgG (Jackson ImmunoResearch; #111-035-144)
Species reactivity: rabbit
Application (in this study): western blot
Validation statements: https://www.jacksonimmuno.com/catalog/products/111-035-144

-HRP-conjugated goat anti-mouse IgG (Jackson ImmunoResearch; #115-035-146)
Species reactivity: mouse
Application (in this study): western blot
Validation statements: https://www.jacksonimmuno.com/catalog/products/115-035-146

## Eukaryotic cell lines

Policy information about cell lines and Sex and Gender in Research

| | |
|---|---|
| Cell line source(s) | All eukaryotic cell lines used in this study can be found in Supplementary Data 2.<br>HeLa cells (ATCC; #CCL-2, female, human)<br>HEK293T (ATCC; #CRL-3216, female, human)<br>Flp-In™ T-REx™ 293 (Invitrogen; #R78007, female, human)<br>RAW264.7 (ATCC; #TIB-71, male, murine)<br>RAW264.7 Cyld KO (Clone 1): single-cell colony of parental line (ATCC; #TIB-71)<br>RAW264.7 Cyld KO (Clone 2): single-cell colony of parental line (ATCC; #TIB-71)<br>RAW264.7 Rnf213 KO (Clone 1): single-cell colony of parental line (ATCC; #TIB-71)<br>RAW264.7 Rnf213 KO (Clone 2): single-cell colony of parental line (ATCC; #TIB-71)<br>Mouse Embryonic Fibroblasts (MEFs) (ATCC; #CRL-2991, murine)<br>L929 Fibroblasts (ATCC; #CCL-1, male, murine)<br>Primary bone marrow-derived macrophages were generated from wildtype male C57BL/6N mice (Charles River Laboratories, Strain #027). |
| Authentication | Cells lines were authenticated on two levels. Firstly, cell lines from ATCC were authenticated by the organization. All cell lines used in this study were authenticated by SickKids BioBank<br>via STR profiling using the GenePrint 10 System. |
| Mycoplasma contamination | All cell lines used were tested negative for mycoplasma contamination. |
| Commonly misidentified lines<br>(See ICLAC register) | No commonly misidentified cell lines were used in the study. |

## Animals and other research organisms

Policy information about studies involving animals; ARRIVE guidelines recommended for reporting animal research, and Sex and Gender in Research

| | |
|---|---|
| Laboratory animals | Wildtype, male C57BL/6N mice were purchased from Charles River Laboratories (strain #: 027) and acclimatized for at least one week in a specific pathogen-free unit (22°C, 40% humidity) maintained on a 12 h light-dark cycle with food and water available ad libitum. Systemic infections were performed on 8-9 week-old mice. |
| Wild animals | This study did not involve wild animals. |
| Reporting on sex | In vivo experiments were strictly performed using male mice based on known sex-dependent differences in susceptibility to Listeria monocytogenes (PMID: 16113316). Due to technical constraints and resource availability, only male mice were examined. |
| Field-collected samples | This study did not involve samples collected from the field. |
| Ethics oversight | All experiments described were carried out in accordance with the Guide for the Humane Use and Care of Laboratory Animals and ethical approval was obtained from The Hospital for Sick Children's Animal Care Committee (AUP #65606). |

Note that full information on the approval of the study protocol must also be provided in the manuscript.

## Plants

| | |
|---|---|
| Seed stocks | N/A |
| Novel plant genotypes | N/A |
| Authentication | N/A |
