# Supplementary material for: Listeria promotes systemic infection by targeting the host deubiquitinase CYLD
Source: Nat Commun. 2026 Aug 31;17:10351. doi: 10.1038/s41467-026-77063-5 (PMC13623971; doi:10.1038/s41467-026-77063-5)
Supplement: Supplementary file 4 [file 41467_2026_77063_MOESM8_ESM.pdf]

nature portfolio

Peer Review File

Listeria promotes systemic infection by targeting the host deubiquitinase CYLD

Corresponding Author: Dr John Brumell

This file contains all reviewer reports in order by version, followed by all author rebuttals in order by version.

Version 0:

Reviewer comments:

Reviewer #1

(Remarks to the Author)
The authors speculated that the secreted Listeria monocytogenes virulence factor InlC (Internalin C) has further roles during infection besides its known binding to TUBA and IKK promoting cell to cell spread and dampen innate immunity signalling, respectively. Using BioID they identified the host deubiquitinase CYLD as an interactor of InlC. By analysing truncations in IP and ITC experiments, they were also able to determine that only the CAP-Gly2 domain of CYLD is required for this interaction. The structure of the InlC/CAP-Gly2 complex was solved and the interface was thoroughly characterised by mutational analysis combined with IP and ITC experiments. Thereby the authors identified a novel binding site within the LRR domain of InlC, which is distinct from the known TUBA/IKK binding site. This enabled the authors to specifically dissect the InlC-dependent role of CYLD in infected cells. Finally, they showed that CYLD is dependent on the presence of InlC and RNF213 recruited to the Lm surface and able to reduce the local amount of K63-linked ubiquitination, contributing to the virulence in infected mice.
This study will have a major impact on the field of bacterial effectors, particularly on the field of ubiquitin-related effectors. Recently, several bacterial strategies have been discovered to prevent RNF213-dependent ubiquitination, but these were based on the secretion of bacterial deubiquitinases or ligases. Instead, Lm recruits a host-derived deubiquitinase for this task. I will not hesitate to recommend this article for acceptance with the following minor points to be considered for improving the manuscript.

Line 127: The GKNDG-motif is shown to be highly conserved in species suffering from listeriois, but is it ONLY conserved in these species and absent from others?
Line 130: In Figure 2C N275 was substituted by an Ala residue for the IP, while in Fig 2D it was substituted by a Glu residue which is a much more drastic change. Although it will make probably no big difference in the analysis of the interface, it would be great to use consistent mutants in the different assays.
Line 136: It would be helpful to add a structure figure to Sup figure 1 which shows were F288 is located in context of the CAP-Gly2/InlC interface
Line 148: For me it remains unclear why this analysis was done. The analysed Lys-residues are distributed over the entire InlC while K236 was part of the identified binding interface.
Line 153: SupFig1e: Please write out the abbreviation “cg2” in full.
Line 153: Why was Ile159 chosen for the conclusion although F182 had a much greater impact on the binding affinity?
Line 207: Add this point of the manuscript it is not clear whether CYLD is recruited to ubiquitinated or non-motile Lm. The later analysis of the RNF213 KO cells (line 230) shows that it is Ub-dependent, but at this point of the manuscript it should be more carefully described

Reviewer #2

(Remarks to the Author)
The authors reported an interesting discovery of Listeria targeting a host deubiquitinase CYLD via a secreted bacterial factor InlC. Mechanistically, the LRR domain of InlC interacts with CYLD through its CAP-Gly2 domain, independently of InlC’s binding site for other host targets. Furthermore, InlC promotes CYLD recruitment to ubiquitinated bacteria in the cytosol of host cells, leading to a significant decrease of K63-linked ubiquitin signals. Functionally, InlC-CYLD binding contributes to Listeria virulence during systemic infection. Overall, the study is well-conducted, though the reviewer has some major

concerns mostly regarding the immunofluorescence data.

1.The authors claim that InlC target CYLD to the bacterial surface, yet there is no evidence of InlC anchoring to the bacterium itself. Without such evidence, the “targeting” model is logically incomplete. Therefore, it would be necessary to acquire high-resolution immunofluorescence images and show direct co-localization of InlC together with CYLD at the bacterial surface during Lm infection.

2.As for the quantification of the IF imaging in Figures 3B and 3D, the recruitment rate is calculated as a percentage of "HA-CYLD positive bacteria". However, in the ΔactA background, bacteria frequently form dense clusters. According to the Methods section, the authors use a "1x dilation tool" to account for the ubiquitin signal surrounding the membrane and it is technically very difficult to distinguish individual bacterial membranes within these clusters. To ensure the accuracy of these statistics, I would suggest these experiments are repeated at a lower MOI to minimize bacterial clustering. Additionally, a more detailed description of the fluorescence quantification workflow should be included in the Methods section as well.

3.Most of the IF imaging data (Figure 3 and Supplementary Figure 3) rely on the transient overexpression of HA-tagged CYLD. It is well recognized that protein overexpression can sometimes lead to non-physiological aggregation or non-specific binding artifacts. To further strengthen the conclusion, I strongly recommend IF staining against endogenous CYLD during infection and make sure that the recruitment patterns remain consistent.

4.The IF experiments rely heavily on the ΔactA mutant. It seems that the WT strain clusters much less than the mutant. The authors should clarify the percentages of WT bacteria that are actually FK2-positive in these assays. Given that the WT represents the natural state of infection, it would be much more convincing if one can show the quantification data of CYLD recruitment on the surface of wild-type bacteria (rather than the ΔactA strain).

Other minor comments:
1.The authors got both the crystal structure of InlC-CAP-Gly2 domain of CYLD and the Alfafold-predicted structure of InlC-BirA2. Is the structure of InlC from these two datasets similar to each other? Does CAP-Gly2 interact with InlC at an interface different from that of BirA2?
2.As for the mass spec analyses of InlC-interacting proteins, it seems that # of replicates in controls is much larger than that in the experimental group (i.e., duplicates). I didn’t quite get the rationale behind this setup.
3.In Fig. 2G, the WB signals of IKK-alpha seem to be quite low in IP samples (actually much lower than the input signals), which makes the conclusion less convincing.

Reviewer #3

(Remarks to the Author)
This study identifies a strategy by which a bacterial effector manipulates host ubiquitin signaling during infection. The authors show that the secreted Listeria effector InlC interacts with the host deubiquitinase CYLD and that this interaction, together with RNF213-dependent ubiquitination, is required for CYLD recruitment to cytosolic bacteria. They further demonstrate that this axis influences bacterial virulence in vivo. The concept that pathogens counteract ubiquitin-mediated host defense by hijacking deubiquitinating enzymes is important and of broad interest to the field of host–pathogen interactions, and several lines of evidence presented here support this model. However, the principal limitation of the study is the lack of mechanistic resolution connecting these molecular events to a defined intracellular antibacterial pathway and, ultimately, to bacterial growth or survival. While the data establish InlC–CYLD interaction and RNF213 dependence, it remains unclear how CYLD recruitment functionally alters host ubiquitin-dependent defenses (e.g., xenophagy, actin-based motility regulation, or inflammatory signaling) and which of these processes explains the observed virulence phenotype. As a result, the study currently bridges molecular interaction and in vivo outcome without demonstrating the causal intracellular mechanism linking them. Addressing this gap would substantially strengthen the impact and mechanistic completeness of the work.

Major concerns
1. The authors show that CYLD localization to bacteria requires both InlC–CYLD interaction and RNF213 activity. However, the mechanistic basis of this recruitment remains unclear. CYLD is a K63-specific deubiquitinase and would be expected to recognize ubiquitin chains independently of InlC. It is therefore difficult to understand how binding to InlC enables CYLD to sense or engage ubiquitinated bacteria. The current data support that InlC–CYLD interaction is necessary, but do not explain how this interaction functionally contributes to CYLD positioning relative to the ubiquitin coat.
2. In addition, the subcellular localization of InlC itself is not clearly addressed. Since the proposed model implies that InlC functions at the bacterial surface to recruit or stabilize CYLD, it would be important to determine:
・Whether InlC accumulates in the vicinity of cytosolic bacteria.
・Whether such localization depends on CYLD and/or RNF213-mediated ubiquitination.
・Whether disruption of CYLD binding alters InlC distribution.
Without these data, the conclusion that InlC directly recruits CYLD to bacteria appears mechanistically overstated. Clarifying the spatial relationship among InlC, CYLD, and the ubiquitin coat would significantly strengthen the proposed model.
3. The manuscript shows that InlC recruits CYLD to remove RNF213-dependent ubiquitin from cytosolic Lm. However, it remains unclear which host defense pathway is functionally counteracted by this deubiquitination. The in vivo virulence phenotype is presented without linking it to a defined intracellular process. Is the primary effect on xenophagy, actin-based motility/cell-to-cell spread, or inflammatory signaling?
4. Although InlC–CYLD interaction is demonstrated by co-IP, CYLD recruitment to bacteria requires RNF213-dependent

ubiquitination. This suggests that ubiquitin serves as a primary scaffold for CYLD localization, while InlC may function in stabilizing or positioning CYLD rather than directly recruiting it. The authors should clarify this hierarchical recruitment model.
5. The study relies on RNF213 knockout to infer that ubiquitination is required for CYLD recruitment. However, RNF213 may have scaffolding functions independent of its ligase activity. The authors should test an E3-deficient RNF213 mutant (e.g., RING domain mutant) to determine whether CYLD localization depends specifically on RNF213 catalytic activity rather than its mere presence.

Minor comments
1. The manuscript alternates between “gram-positive” and “Gram-positive.” Please standardize capitalization throughout.
2. Both spellings “Ubiquitination” vs “ubiquitylation” are used. Please adopt a single convention according to journal style.
3. Methods wording
•“scarified” should be “sacrificed”
•“bacterial/mL” should read “bacteria/mL”
•“Clonetech” should be “Clontech”
•“Tween 20” vs “Tween-20” should be unified

Version 1:

Reviewer comments:

Reviewer #2

(Remarks to the Author)
The authors have addressed the core technical concerns raised in the initial review. While some mechanistic gaps remain regarding the spatial recruitment model, the newly added experimental data have strengthened the manuscript.

The new immunofluorescence data showed that InlC does not accumulate at the bacterial surface alongside CYLD. Therefore, the exact mechanism underlying this recruitment remains undefined. The authors' choice to tone down their language, shifting from descriptive terms like "targeting" to strictly defining it as a biochemical "protein-protein interaction," is a necessary and responsible correction. While the proposed displacement or transient "hit-and-run" models in the Discussion are speculative, they have properly recognized this structural limitation.

The endogenous CYLD staining experiment is a useful addition, especially with the Cyld KO control. However, the signal appears relatively weak, and the manuscript should clearly acknowledge that most localization data still rely on HA-CYLD expression.

Overall, I am satisfied with the authors’ responses and would support publication of this work upon minor revisions to avoid overstatement of a direct targeting/recruitment mechanism and to clearly state current limitations of the imaging data.

Reviewer #3

(Remarks to the Author)
I thank the authors for their careful and constructive revisions. The revised manuscript includes several new experiments and textual changes that substantially address my previous concerns, particularly regarding InlC localization, CYLD recruitment, and the hierarchical relationship between bacterial ubiquitination and CYLD association.
Although some mechanistic details remain unresolved, including the precise downstream antibacterial pathway and the specific requirement for RNF213 catalytic activity, the authors have reasonably acknowledged these limitations. I do not think these remaining issues compromise the main conclusions of the study.
I am satisfied with the revision and support acceptance of the manuscript.

Open Access This Peer Review File is licensed under a Creative Commons Attribution 4.0 International License, which permits use, sharing, adaptation, distribution and reproduction in any medium or format, as long as you give appropriate credit to the original author(s) and the source, provide a link to the Creative Commons license, and indicate if changes were made.
In cases where reviewers are anonymous, credit should be given to 'Anonymous Referee' and the source.
The images or other third party material in this Peer Review File are included in the article's Creative Commons license, unless indicated otherwise in a credit line to the material. If material is not included in the article's Creative Commons license and your intended use is not permitted by statutory regulation or exceeds the permitted use, you will need to obtain permission directly from the copyright holder.
To view a copy of this license, visit https://creativecommons.org/licenses/by/4.0/

Reviewer #1:

"The authors speculated that the secreted Listeria monocytogenes virulence factor InlC (Internalin C) has further roles during infection besides its known binding to TUBA and IKK promoting cell to cell spread and dampen innate immunity signalling, respectively. Using BioID they identified the host deubiquitinase CYLD as an interactor of InlC. By analysing truncations in IP and ITC experiments, they were also able to determine that only the CAP-Gly2 domain of CYLD is required for this interaction. The structure of the InlC/CAP-Gly2 complex was solved and the interface was thoroughly characterised by mutational analysis combined with IP and ITC experiments. Thereby the authors identified a novel binding site within the LRR domain of InlC, which is distinct from the known TUBA/IKK binding site. This enabled the authors to specifically dissect the InlC-dependent role of CYLD in infected cells. Finally, they showed that CYLD is dependent on the presence of InlC and RNF213 recruited to the Lm surface and able to reduce the local amount of K63-linked ubiquitination, contributing to the virulence in infected mice.
This study will have a major impact on the field of bacterial effectors, particularly on the field of ubiquitin-related effectors. Recently, several bacterial strategies have been discovered to prevent RNF213-dependent ubiquitination, but these were based on the secretion of bacterial deubiquitinases or ligases. Instead, Lm recruits a host-derived deubiquitinase for this task. I will not hesitate to recommend this article for acceptance with the following minor points to be considered for improving the manuscript".

Response: We thank the reviewer for their supportive and constructive comments.

"Line 127: The GKNDG-motif is shown to be highly conserved in species suffering from listeriois, but is it ONLY conserved in these species and absent from others?"

Response: *L. monocytogenes* can elicit listeriosis in a broad range of species, challenging our ability to assess whether the GKNDG-motif is absent in species that do not develop this disease. To clarify the conservation of the GKNDG motif across different groups of hosts susceptible to *Lm* infection, we have updated Figure 2B (see below) by expanding and annotating the sequence alignment to include the following categories: primates, rodents, and livestock:

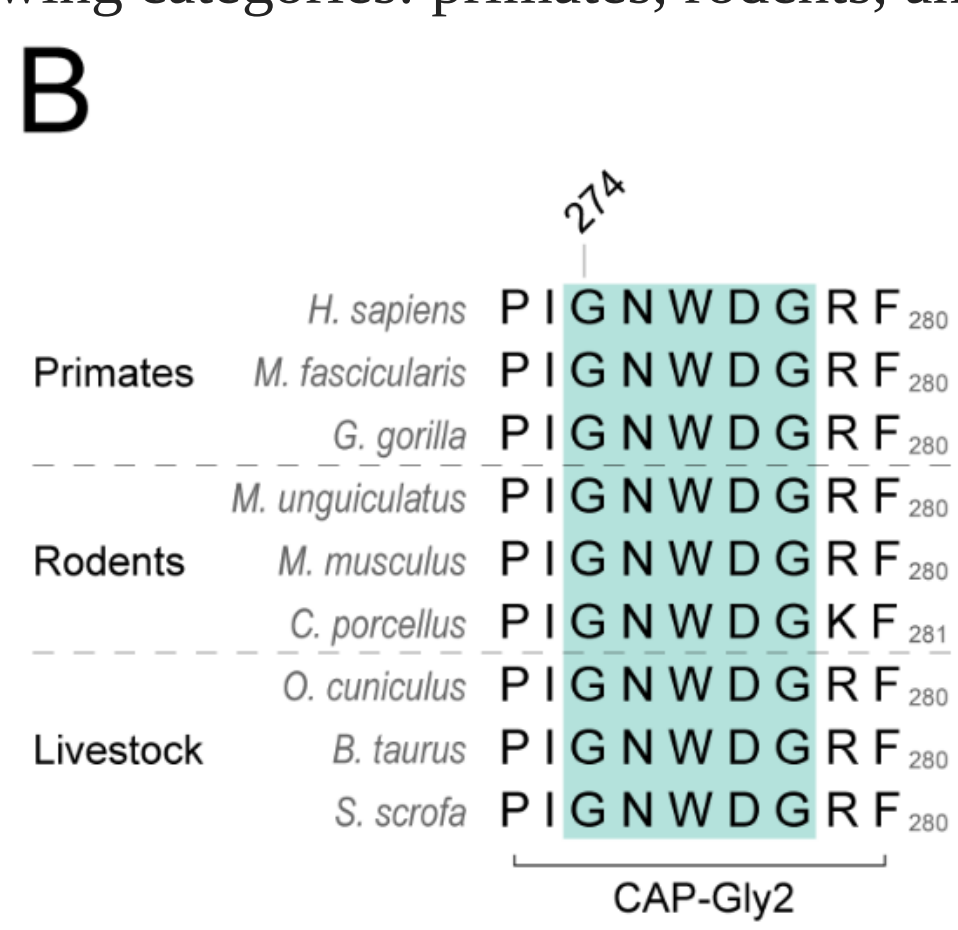

In contrast to vertebrate species, only one CAP-Gly domain is present in invertebrates susceptible to *L. monocytogenes* infection (*C. elegans* and *D. melanogaster*). As poor conservation of the 'GKNDG' motif is observed (see alignment below), we decided to omit this analysis from the manuscript as we felt it was too speculative in nature:

% Identity (BLAST Alignment)

H. sapiens	*D. melanogaster* CAP-Gly 143-219	*C. elegans* CAP-Gly 460-522
CAP-Gly1 127-203	25.56%	27.78%
CAP-Gly2 232-302	28.57%	19.72%
CAP-Gly3 472-540	45.45%	30.61%

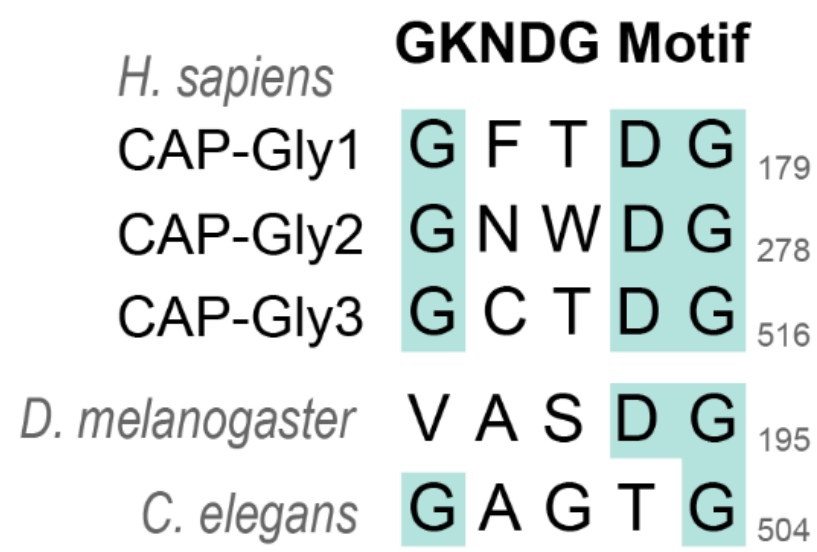

"Line 130: In Figure 2C N275 was substituted by an Ala residue for the IP, while in Fig 2D it was substituted by a Glu residue which is a much more drastic change. Although it will make probably no big difference in the analysis of the interface, it would be great to use consistent mutants in the different assays."

Response: We thank the reviewer for this comment. We used different Asn275 mutations to gain a detailed understanding of the CYLD-InlC interface. First, we performed alanine scanning mutagenesis as a screen to assess which of the five residues within the 'GKNDG' motif were implicated in InlC-CYLD binding where we observed a reduction, but not a complete loss, of interaction with loss of the Asn275 side chain interaction (N275A). This was surprising given the observed position of Asn275 in the middle of the CYLD-InlC interface.

To investigate the contribution of this interaction further, we tested the steric contribution (N275Q which weakened the affinity by ~8 fold) or the effect of introducing a repulsive charge (N275E) which weakened the interaction further still (~60 fold reduction overall). Together our extended mutational analyses indicate that Asn275 in the 'GKNDG' motif contributes to the CYLD-InlC interaction.

We have updated the text in the results section to clarify the use of these N275 mutations:

Page 4, line132 from revised manuscript:
ITC analysis additionally confirmed that mutation of G274A prevented recombinant InlC binding, whereas mutation of CYLD Asn275 to introduce steric hinderance (N275Q) or a repulsive charge (N275E) only reduced the affinity for InlC (Fig. 2D).

"**Line 136: It would be helpful to add a structure figure to Sup figure 1 which shows were F288 is located in context of the CAP-Gly2/InlC interface"**

Response: We have updated the new Supplementary Figure 2A to include a structure figure highlighting where CAP-Gly2 Phe288 (ubiquitin-binding) lies relative to Gly274 (InlC binding) at the CAP-Gly2/InlC interface:

A

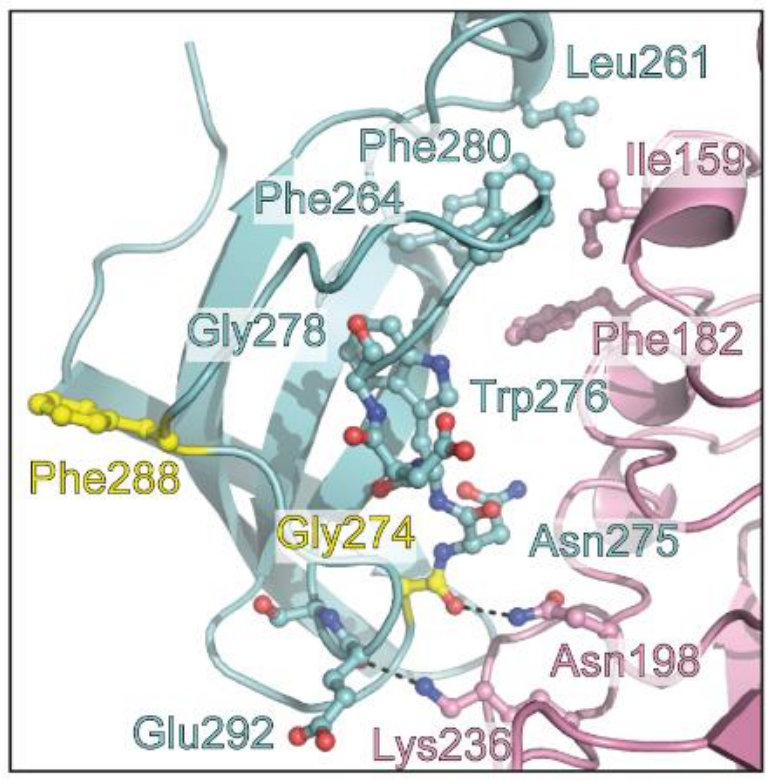

"**Line 148: For me it remains unclear why this analysis was done. The analysed Lys-residues are distributed over the entire InlC while K236 was part of the identified binding interface."**

Response: A previous report (PMID: 31848284) revealed that InlC is targeted for monoubiquitination during infection on Lys244 and that this modification impacts the ability of InlC to interact with host machinery (e.g. InlC-S100A9).

While InlC Lys236 mediates CYLD binding at the protein interface, we postulated that host ubiquitin-mediated regulation of InlC at distal sites (e.g. ubiquitination of Lys244) may impact the interaction between InlC and CYLD. Alongside Lys244, we performed alanine scanning mutagenesis to rule out the involvement of other lysine residues in regulating InlC-CYLD binding, potentially through host modifications (e.g. ubiquitination, SUMOylation).

We have clarified our rationale for this experiment in the results section:

Page 5, line148 from revised manuscript:
Previous work revealed that host-mediated ubiquitination of InlC impacts its interaction with host proteins during *Lm* infection[27]. This led us to question whether modification of other InlC lysine residues, potential targets of ubiquitination[20], influenced binding to CYLD in a cellular context. However, mutagenesis of all other lysine residues of InlC did not affect CYLD binding (Supplementary Fig. 2D and 2E).

"Line 153: SupFig1e: Please write out the abbreviation "cg2" in full."

Response: We have corrected the new Supplementary Figure 2F (formerly Supp. Fig. 1E), as shown below:

F

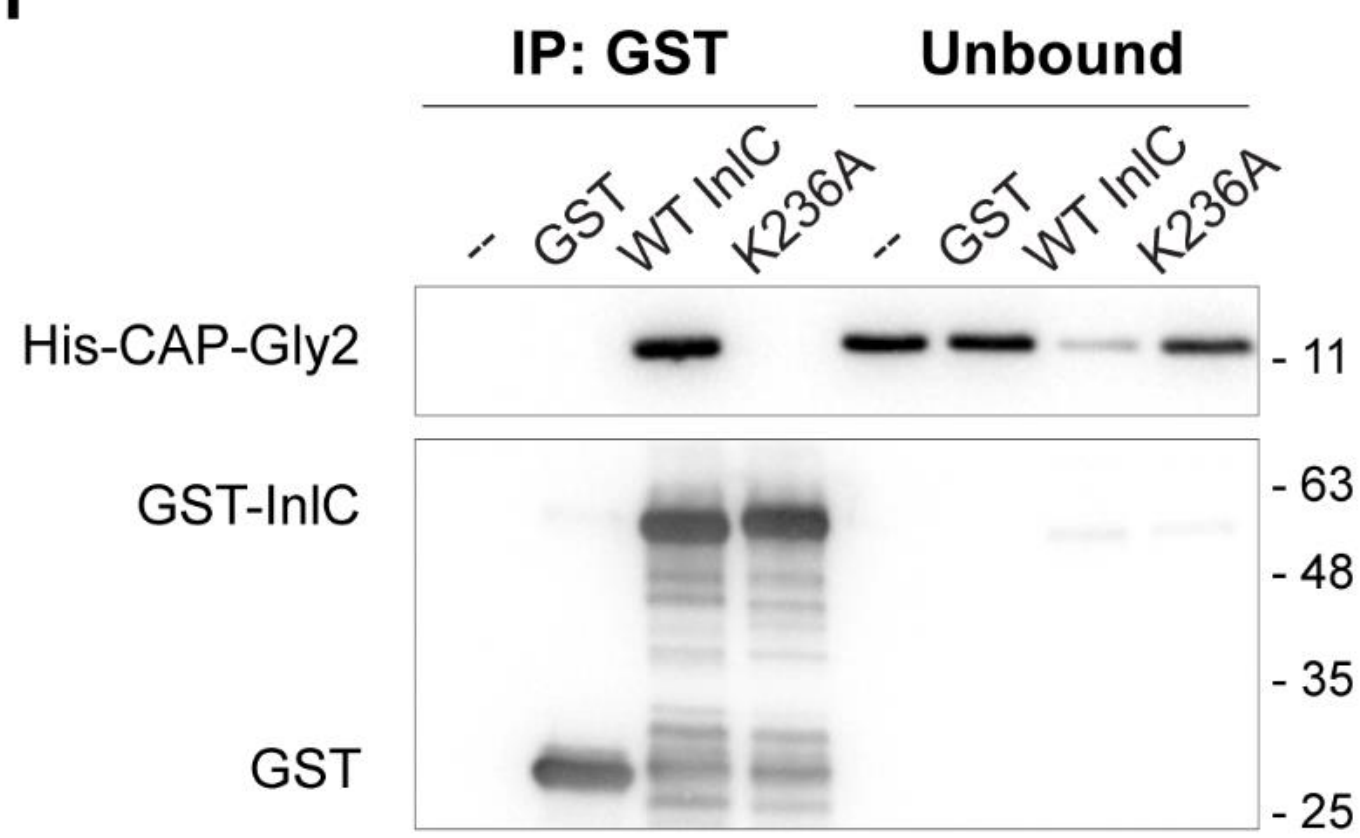

"Line 153: Why was Ile159 chosen for the conclusion although F182 had a much greater impact on the binding affinity?"

Response: The purpose of these mutagenesis experiments was to identify an InlC mutation that would abolish binding to CAP-Gly2. We have rearranged the order of how we discuss these mutations to emphasize the deleterious impact of I159D:

Page 5, line144 from revised manuscript:
Mutation of bulky hydrophobic residues I159A or F182A within InlC reduced binding affinity to CAP-Gly2 (4-fold and 166-fold, respectively) whereas introduction of a negative charge abolished (I159D) or severely reduced (F182E, 2,568-fold) binding.

"Line 207: Add this point of the manuscript it is not clear whether CYLD is recruited to ubiquitinated or non-motile Lm. The later analysis of the RNF213 KO cells (line 230) shows that it is Ub-dependent, but at this point of the manuscript it should be more carefully described"

Response: A similar point was raised by Reviewer #2 (Comment 4). In response, we have performed new experiments to further address this question. Our new data are described in a new results section (see below) and the data is presented in the new Supplementary Figure 9A and 9B (see below).

Page 7, line 248 from revised manuscript:
CYLD is recruited to the bacterial surface prior to actin-based motility
Previous studies highlighted the critical role of ActA in evading host-ubiquitin defenses[10,31,32]. As Δ*actA* bacteria are vulnerable to detection by host ubiquitin defenses, we questioned the motility status of CYLD-positive WT *Lm*. Co-staining of HA-CYLD alongside filamentous actin (F-actin) revealed that the majority of CYLD-positive WT *Lm* were surrounded by non-polarized actin (referred to as actin clouds), indicating that the bacteria were not yet motile (**Supplementary Fig. 9A and 9B**). Only a small fraction of CYLD-positive *Lm* was devoid of polymerized actin, and in rare instances, displayed a comet tail. In agreement with these observations, we found that actin clouds were present at most ubiquitin-positive WT and Δ*inlC* bacteria (**Supplementary Fig. 9C and 9D**). Notably, amongst motile strains we observed a modest yet significant increase in the frequency of ubiquitin-positive bacteria upon loss of InlC (**Supplementary Fig. 9E**). These observations led us to speculate that the InlC-dependent recruitment of CYLD to the bacterial surface serves to accelerate ubiquitin clearance prior to actin-based motility.

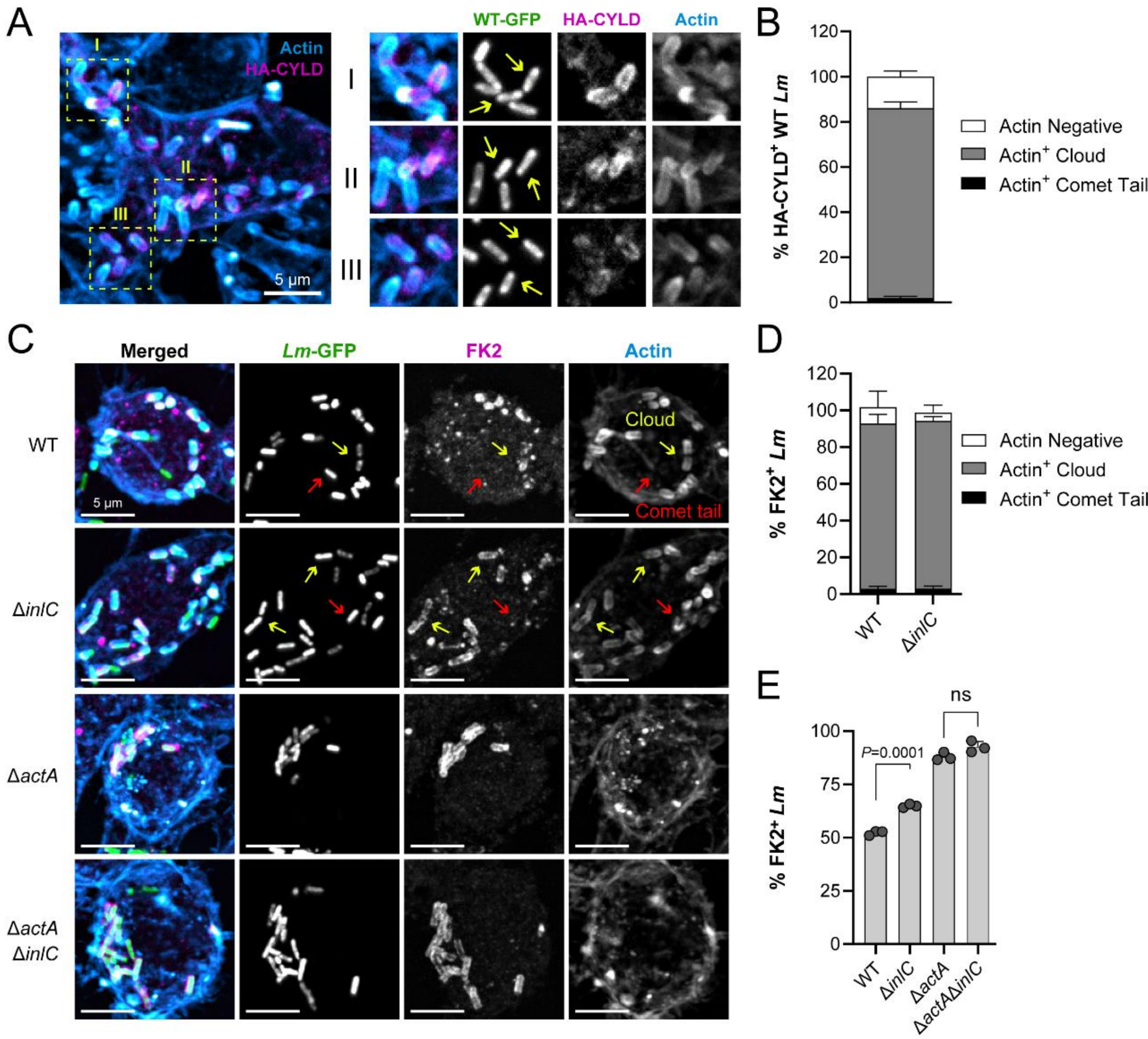

Supplementary Figure 9. HA-CYLD and ubiquitin localize with *Lm* prior to actin-based motility.

(A-B) HA-CYLD and F-actin staining during infection of RAW264.7 cells with WT *Lm* (4 hours post-infection). Arrows highlight individual bacteria within regions of interest that are both HA-CYLD and F-Actin-positive. Scale bar, 5 µm. Images are representative of three experimental replicates. (B) Quantification of HA-CYLD-positive *Lm* that were either negative, or positive for F-actin in the form of an actin cloud or comet tail. Mean ± SD is displayed based on quantification of (15-19) RAW264.7 cells per experimental replicate.

(C-E) Ubiquitin (FK2) and F-actin staining during infection of RAW264.7 cells with either motile or non-motile *Lm* (4 hours post-infection). Yellow and red arrows highlight individual bacteria displaying either an actin cloud or comet tail, respectively. Scale bar, 5 µm. Images are representative of three experimental replicates. (D) Quantification of FK2-positive *Lm* that were either negative, or positive for F-actin in the form of an actin cloud or comet tail. (E) Quantification of FK2-positive *Lm* per infected cell. Quantifications display mean ± SD based on quantification of (25-30) RAW264.7 cells per experimental replicate. *p* values were determined using an unpaired t-test.

Reviewer #2:

"The authors reported an interesting discovery of Listeria targeting a host deubiquitinase CYLD via a secreted bacterial factor InlC. Mechanistically, the LRR domain of InlC interacts with CYLD through its CAP-Gly2 domain, independently of InlC's binding site for other host targets. Furthermore, InlC promotes CYLD recruitment to ubiquitinated bacteria in the cytosol of host cells, leading to a significant decrease of K63-linked ubiquitin signals. Functionally, InlC-CYLD binding contributes to Listeria virulence during systemic infection. Overall, the study is well-conducted, though the reviewer has some major concerns mostly regarding the immunofluorescence data."

Response: We thank the reviewer for their supportive and constructive comments.

"1.The authors claim that InlC target CYLD to the bacterial surface, yet there is no evidence of InlC anchoring to the bacterium itself. Without such evidence, the "targeting" model is logically incomplete. Therefore, it would be necessary to acquire high-resolution immunofluorescence images and show direct co-localization of InlC together with CYLD at the bacterial surface during Lm infection."

Response: We have added a new results section (see below) to accompany a new Supplementary Figure 7 (see below) highlighting the lack of InlC accumulation alongside ubiquitin- or HA-CYLD-positive bacteria.

Page 6, line 217 from revised manuscript:
InlC does not accumulate alongside CYLD at the bacterial surface
Next, we asked if InlC also accumulates at the surface of ubiquitin-positive bacteria. For this, we infected cells with *Lm* expressing pLOV-*inlC*-FLAG for complementation of Δ*inlC Lm* with either wildtype *inlC* or single point mutants defective in binding to CYLD (I159D, K236A). At 4 hours post infection, cells were fixed and stained for the FLAG epitope to visualize localization of InlC-FLAG. We observed a diffuse, cytosolic signal for InlC that was above the background of the FLAG epitope antibody observed in cells infected with bacteria with empty vector (i.e. no FLAG tag expressed), in accordance with previous studies[13,21] (**Supplementary Fig. 7A**). However, we did not observe enrichment of InlC at ubiquitin-positive bacteria, including non-motile *Lm* (Δ*actA*Δ*inlC*). Similar cytosolic staining was observed in *Lm* strains expressing either WT InlC or CYLD-defective binding mutants, suggesting that InlC localization in the cytosol is unaltered by CYLD binding. Accordingly, we did not detect co-enrichment of InlC alongside HA-CYLD at ubiquitin-positive bacteria using both motile and non-motile *Lm* (**Supplementary Fig. 7B**). Taken together, these results suggest that InlC does not accumulate at the surface of ubiquitin-positive *Lm* alongside CYLD.

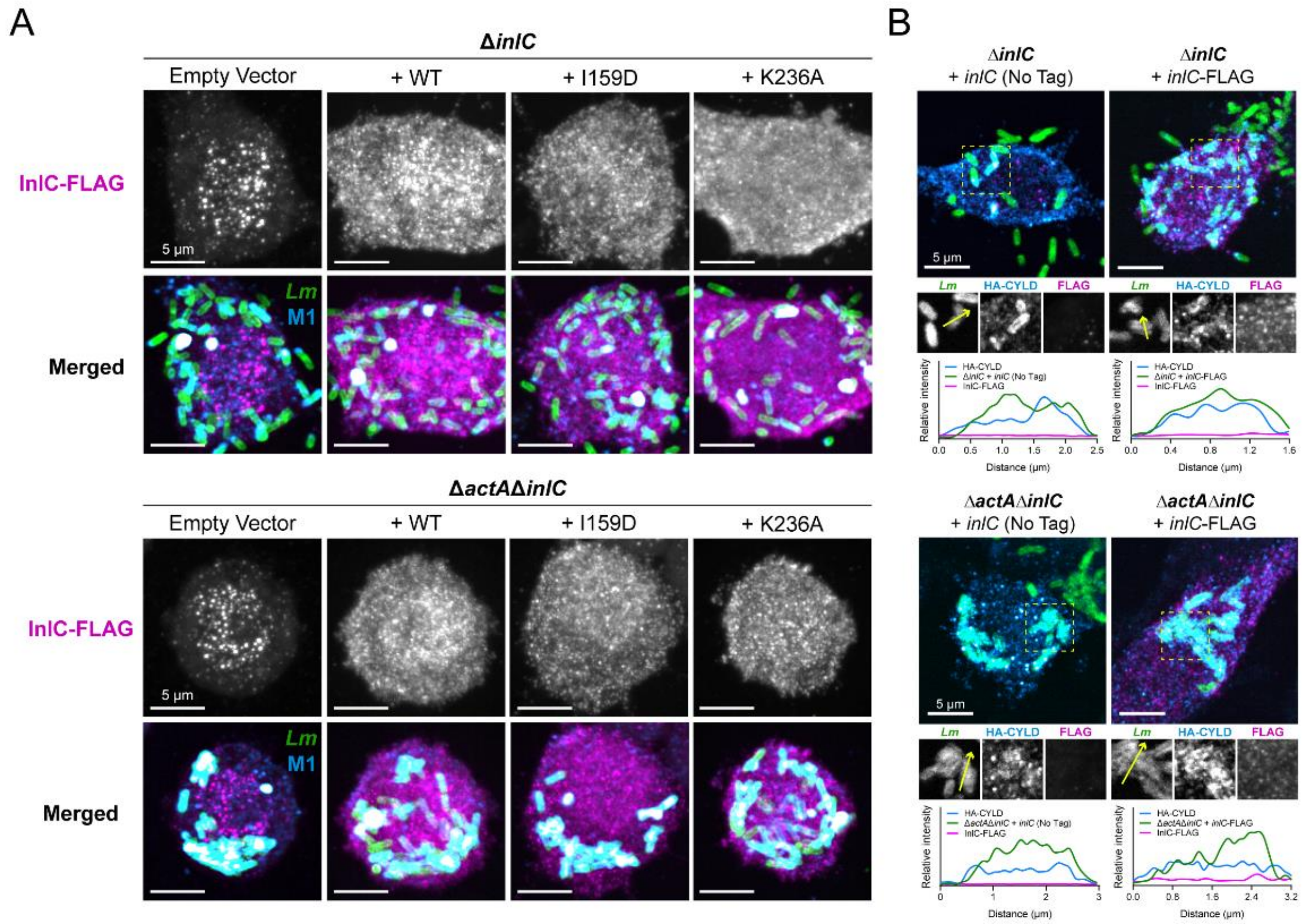

Supplementary Figure 7. InlC is not recruited to ubiquitin- or HA-CYLD-positive *Lm*. (A) InlC-FLAG localization in RAW264.7 cells infected with motile (Δ*inlC*), or non-motile (Δ*actA*Δ*inlC*) *Lm* complemented with pLOV-*inlC*-FLAG, expressing either wildtype *inlC* or single point mutants defective in binding to CYLD (I159D, K236A) (4 hours post-infection). Met1-linked ubiquitin was stained due to same species conflict for antibodies against FLAG and FK2. Scale bar, 5 µm. Images are representative of three experimental replicates.
(B) HA-CYLD and InlC-FLAG localization in RAW264.7 cells infected with motile (Δ*inlC*), or non-motile (Δ*actA*Δ*inlC*) *Lm* complemented with either wildtype *inlC* or *inlC*-FLAG (4 hours post-infection). Scale bar, 5 µm. Relative intensity profiles correspond to the arrow in the *Lm* channel. Images are representative of three experimental replicates.

Based on these results, we have modified our use of the word "target/targeting" to strictly describe the protein-protein interaction between InlC and CYLD (see Lines #81, 316, 393 of the revised manuscript). Furthermore, we have updated the Discussion to highlight potential mechanisms by which InlC promotes CYLD recruitment to ubiquitin-positive *Lm*:

Page 9, line 334 from revised manuscript:

How does InlC promote recruitment of CYLD to the bacterial surface? The lack of InlC enrichment at the surface of ubiquitin-positive bacteria suggests several possible mechanisms. First, InlC may traffic CYLD to the bacterial surface and then become rapidly displaced upon the association of CYLD with target substrates. Such a dynamic interaction of InlC with the bacterial surface would likely be undetected with the immunofluorescence microscopy approach applied in this study. Second, InlC-CYLD binding may operate through a displacement model,

analogous to InlC-TUBA binding which involves the displacement of N-WASP[14]. In this scenario, InlC binding to the CAP-Gly2 domain would displace CYLD from preexisting locations, such as microtubules and immune receptors[25,26]. In support of this mechanism, recent evidence highlights the peptide-binding abilities of CYLD's CAP-Gly domains which serve as auxiliary regions to regulate deubiquitinase localization[37]. Third, it remains possible that another host or bacterial factor is required to translocate CYLD to the bacterial surface. The inability of InlC to promote the recruitment of a truncated form of CYLD comprising only CAP-Gly1-3, despite successful binding, revealed a requirement for CYLD's C-terminus for successful recruitment. In the context of immune signaling, the B-box-dependent dimerization of CYLD permits binding to SPATA2, an adaptor protein that facilitates CYLD recruitment to immune receptors[23]. Our identification of the B-box as a necessary determinant for CYLD translocation suggests a similar role for CYLD dimerization and additional host factors to mediate CYLD delivery.

"2.As for the quantification of the IF imaging in Figures 3B and 3D, the recruitment rate is calculated as a percentage of "HA-CYLD positive bacteria". However, in the ΔactA background, bacteria frequently form dense clusters. According to the Methods section, the authors use a "1x dilation tool" to account for the ubiquitin signal surrounding the membrane and it is technically very difficult to distinguish individual bacterial membranes within these clusters. To ensure the accuracy of these statistics, I would suggest these experiments are repeated at a lower MOI to minimize bacterial clustering. Additionally, a more detailed description of the fluorescence quantification workflow should be included in the Methods section as well."

<u>Response:</u> Firstly, we appreciate this reviewer's attention to detail. The quantification of HA-CYLD-positive bacteria presented in Figures 3B and 3D was performed manually. Despite forming clusters, we were able to accurately distinguish and score individual Δ*actA* bacteria by scanning through XY slices of our confocal images. Meanwhile, clustering challenged our ability to analyze the ubiquitin signal surrounding individual Δ*actA* bacteria which is why dilation was applied (see panel A below):

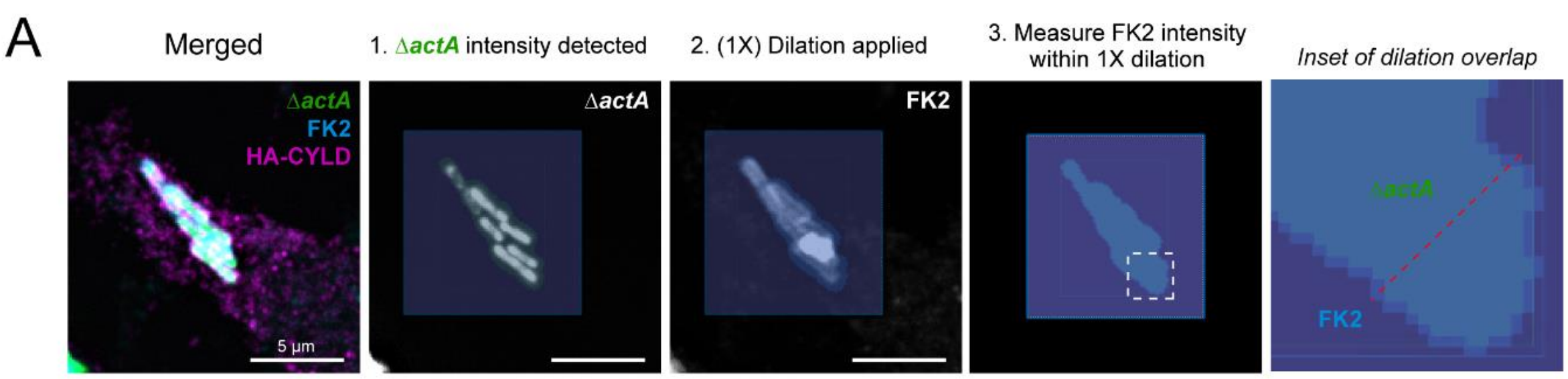

We have updated our methods section to better detail how each immunofluorescence experiment was quantified:

Page 17, line 671 from revised manuscript:
Immunofluorescence Quantification

Confocal images of *Lm*-infected cells were analyzed in Volocity as follows. For quantification of HA-CYLD-positive *Lm* (%) (Fig. 3B, D, F, Supplementary Fig. 4C, Supplementary Fig. 9B, Supplementary Fig. 10C), individual bacteria were manually scored from randomly selected transfected cells. For quantification of ubiquitin (FK2)-positive *Lm* (%) (Supplementary Fig. 4D, Supplementary Fig. 9D and 9E), individual bacteria were manually scored from transfected cells that displayed a ubiquitin response against *Lm* (>1 FK2+ bacteria). For quantification of ubiquitin (FK2)-positive Δ*actA* clusters (Supplementary Fig. 11C), (35-50) clusters were manually scored across (5-8) randomly selected fields of view.

For quantification of ubiquitin (FK2, Lys63- or Met1-linked) intensity (Supplementary Fig. 10D), confocal planes were merged and single clusters of HA-CYLD-positive bacteria were selected across (>15) individual cells per experimental replicate. Using Volocity, a region of interest was determined based on *Lm*-GFP intensity, applying the dilation tool (1x) to account for ubiquitin signal surrounding the bacterial membrane. The sum of intensity for ubiquitin fluorescence was recorded and normalized to the area (μm^2) of the region of interest.

For quantification of *Lm* cell-to-cell spread (Supplementary Fig. 12D and 12F), regions of interest were manually drawn around *Lm*-infected regions, and the area (μm^2) was recorded using Volocity.

Lastly, while we acknowledge the rationale for repeating these experiments with a lower MOI, we observed clustering of the Δ*actA* strain across a range of MOIs (see panel B and C below). This suggests that in our experimental model, clustering is inherent to the Δ*actA* strain rather than a result of multiple bacteria infecting a single cell. A higher MOI was especially important for transfection experiments to increase the likelihood of infecting a transfected cell. As well described (PMID: 26132366), RAW264.7 macrophages are difficult to transfect as we qualitatively observed roughly 1-3% transfection efficiency per experiment.

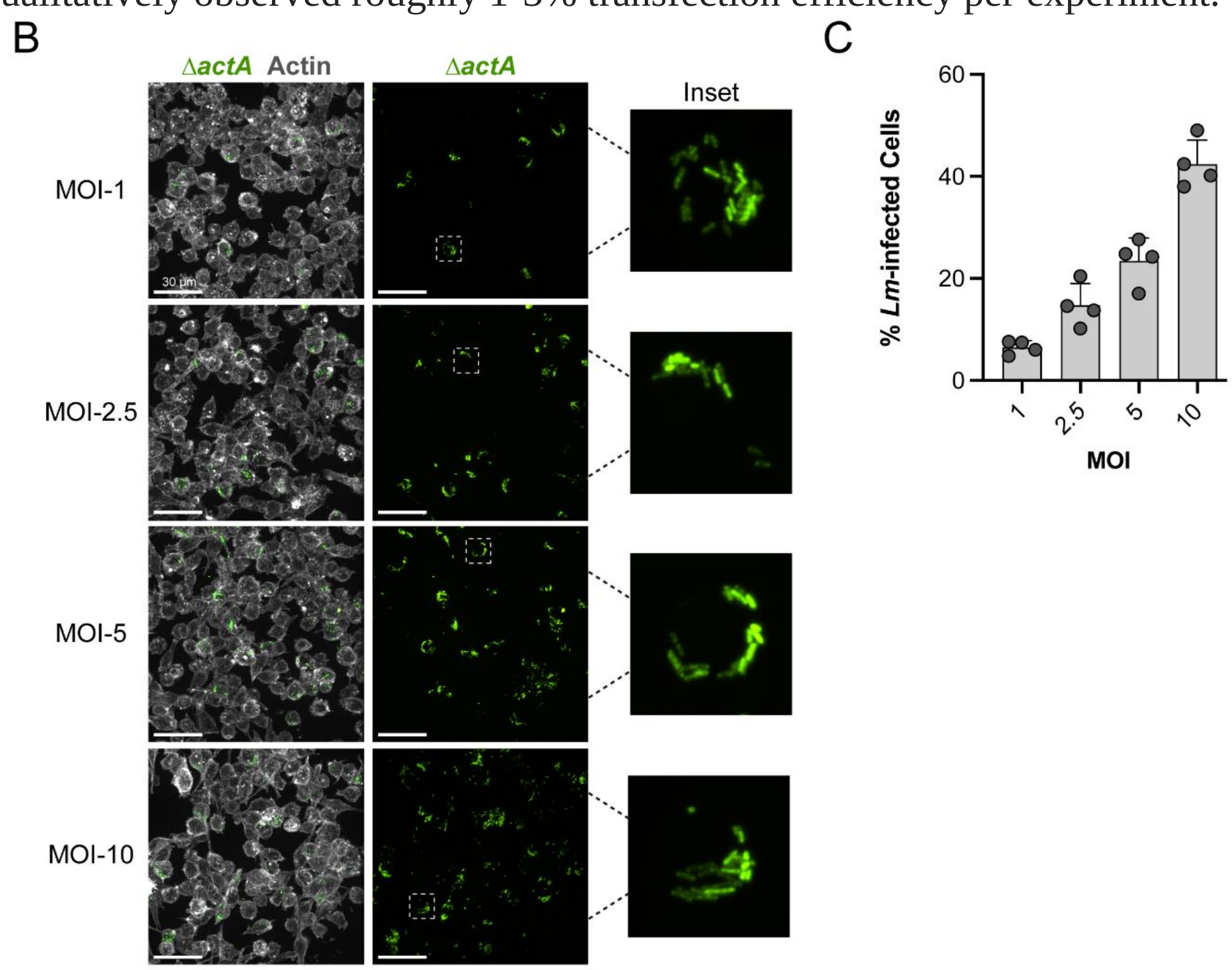

"3.Most of the IF imaging data (Figure 3 and Supplementary Figure 3) rely on the transient overexpression of HA-tagged CYLD. It is well recognized that protein overexpression can sometimes lead to non-physiological aggregation or non-specific binding artifacts. To further strengthen the conclusion, I strongly recommend IF staining against endogenous CYLD during infection and make sure that the recruitment patterns remain consistent."

Response: In response to this reviewer's concern, we tested 5 commercially available antibodies against CYLD. To verify specificity, we used these antibodies to stain *Cyld*$^{-/-}$ RAW264.7 cells complemented with myc-tagged Cyld (mouse origin). An important control was to show that transfected cells expressed the myc-tagged Cyld, as determined by staining cells with an anti-Myc antibody (see panel A, left side, below):

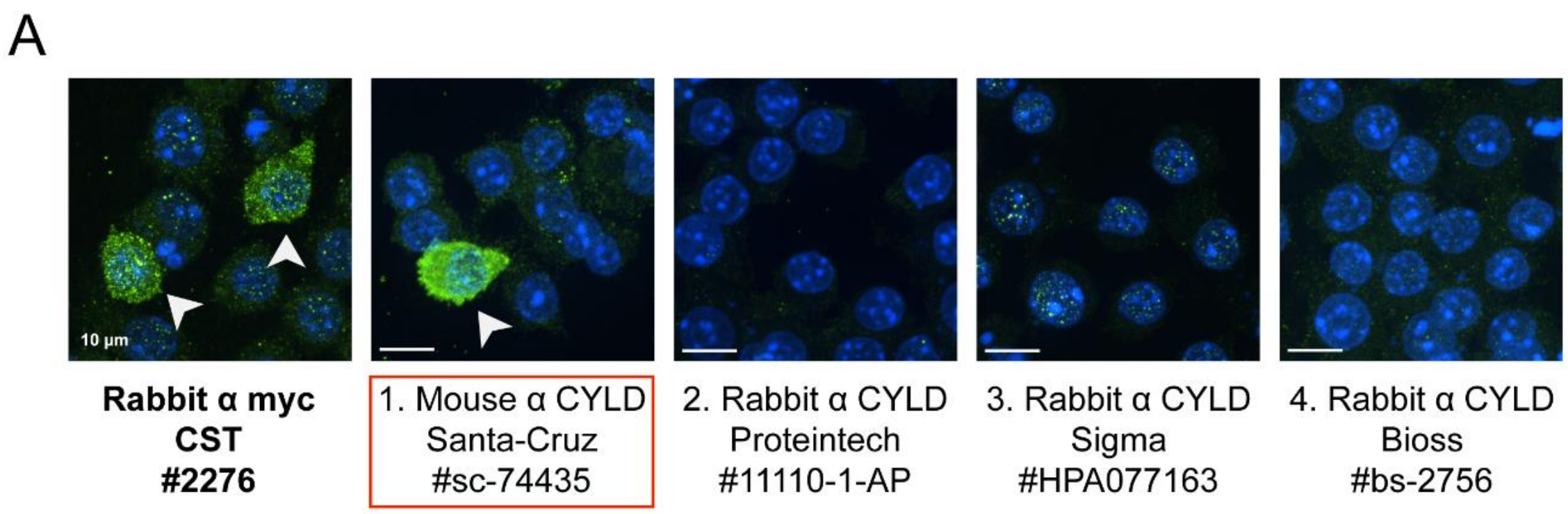

Of the 5 CYLD antibodies that we tested, one antibody (#sc-74435) provided a sufficient signal to detect CYLD in the transfected cells but not the untransfected cells (see red box in panel A above).

We then confirmed that this CYLD antibody produced a higher fluorescence signal, albeit a marginal difference, in wildtype RAW264.7 cells compared to *Cyld*$^{-/-}$ cells (see panel B, below):

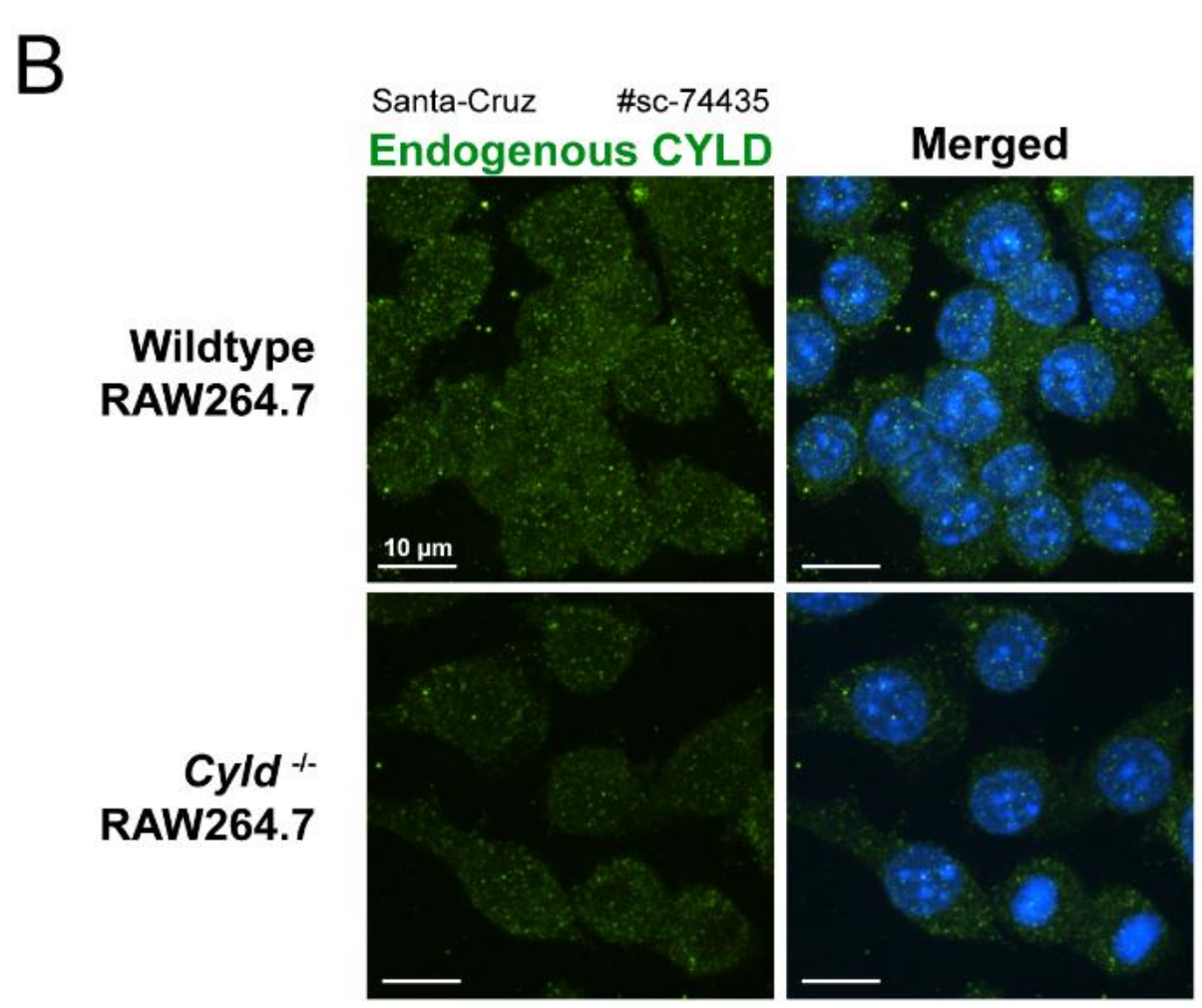

Using this antibody, we were able to detect endogenous CYLD recruitment to both motile and non-motile *Lm*. This new data is now included as a new Supplementary Figure 5 (see below):

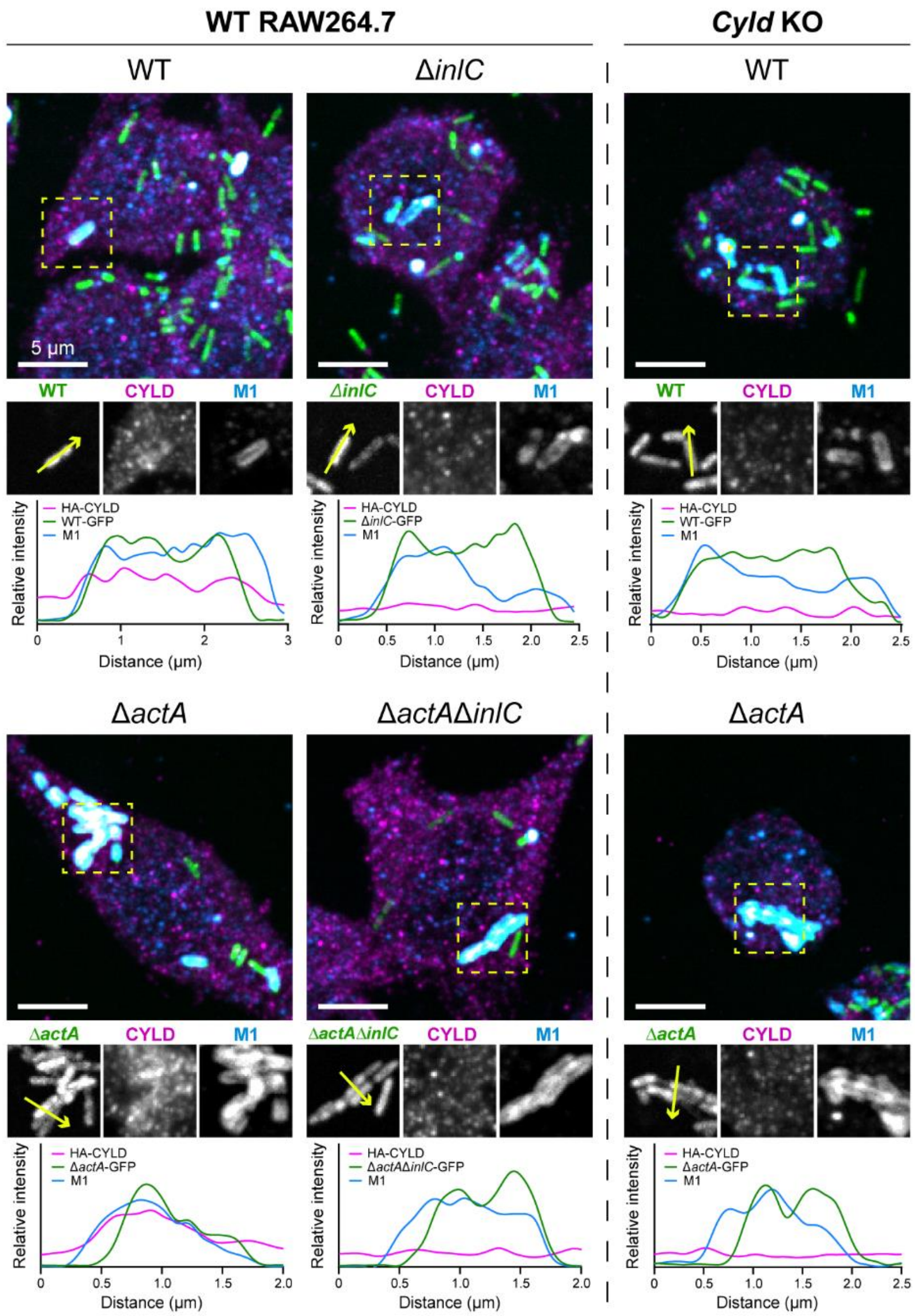

Supplementary Figure 5. Detection of endogenous CYLD at ubiquitin-positive *Lm*. Following 4 hours post-infection with either motile or non-motile *Lm*, WT or *Cyld* KO RAW264.7 cells were stained for endogenous CYLD and ubiquitin (Met1-linked). Met1-linked ubiquitin was stained due to same species conflict for antibodies against endogenous CYLD and FK2. Scale bar, 5 μm. Relative intensity profiles correspond to the arrow in the *Lm* channel. Images are representative of three experimental replicates.

“4.The IF experiments rely heavily on the ΔactA mutant. It seems that the WT strain clusters much less than the mutant. The authors should clarify the percentages of WT bacteria that are actually FK2-positive in these assays. Given that the WT represents the natural state of infection, it would be much more convincing if one can show the quantification data of CYLD recruitment on the surface of wild-type bacteria (rather than the ΔactA strain).”

Response: We appreciate this reviewer’s concern about our usage of the Δ*actA* mutant, and we agree that the WT strain represents the natural state of infection. This led to additional experiments that clarified the recruitment of HA-CYLD to WT *Lm* in relation to ubiquitin and ActA-mediated actin polymerization. Reviewer #1 had a similar comment. Please refer to our response to Reviewer #1 (Page 5 above, in “Line207” comment) which outlines a new results section that corresponds to a new Supplementary Figure 9.

“Other minor comments:
1.The authors got both the crystal structure of InlC-CAP-Gly2 domain of CYLD and the Alfafold-predicted structure of InlC-BirA2. Is the structure of InlC from these two datasets similar to each other? Does CAP-Gly2 interact with InlC at an interface different from that of BirA2?”

Response: The InlC-CAP-Gly2 model was determined experimentally through X-ray crystallography whereas InlC-BirA* Alphafold2 modelling was performed as a control to confirm no interactions between InlC and BirA*. The Predicted Alignment Error (PAE) plot (new Supplementary Figure 1) showed no interactions confirming the BioID strategy would capture InlC interaction partners.

“2.As for the mass spec analyses of InlC-interacting proteins, it seems that # of replicates in controls is much larger than that in the experimental group (i.e., duplicates). I didn’t quite get the rationale behind this setup.”

Response: The BioID dataset for InlC was a part of a larger screen of *Lm* virulence factors, each of which had its own set of corresponding controls. All experiments were performed on the same day, likewise for processing via LC/MS-MS. To avoid bias, we included all 12 control samples in our dataset. Notably, for all the positive hits identified by InlC-BirA* (Figure 1B), no peptides were detected across all 12 control samples (see table below):

Protein	Sum	InlC-BirA*	BirA*
CYLD	**323**	168 | 155	0 | 0 | 0 | 0 | 0 | 0 | 0 | 0 | 0 | 0 | 0 | 0
SPATA2	**116**	64 | 52	0 | 0 | 0 | 0 | 0 | 0 | 0 | 0 | 0 | 0 | 0 | 0
IKK-α	**30**	12 | 18	0 | 0 | 0 | 0 | 0 | 0 | 0 | 0 | 0 | 0 | 0 | 0
IKK-γ	**19**	13 | 6	0 | 0 | 0 | 0 | 0 | 0 | 0 | 0 | 0 | 0 | 0 | 0

BFDR < 0.01 Peptide Count (N1 | N2)

“3.In Fig. 2G, the WB signals of IKK-alpha seem to be quite low in IP samples (actually much lower than the input signals), which makes the conclusion less convincing.”

<u>Response:</u> We acknowledge that the IKK-α signal is lower in the IP samples obtained from primary macrophages. This representative image reflects our best efforts to improve the signal including the use of a high sensitivity ECL substrate and loading ~65 µg of protein lysate (25% v/v).

This low signal obtained from primary macrophage lysates was our rationale for repeating this experiment in HeLa cells (Supplementary Figure 3D).

Alongside confirming our results in HeLa cells (InlC F146A is unable to bind IKK-α), we also noticed that the signal of bound IKK-α was far weaker than that of non-bound IKK-α (see below):

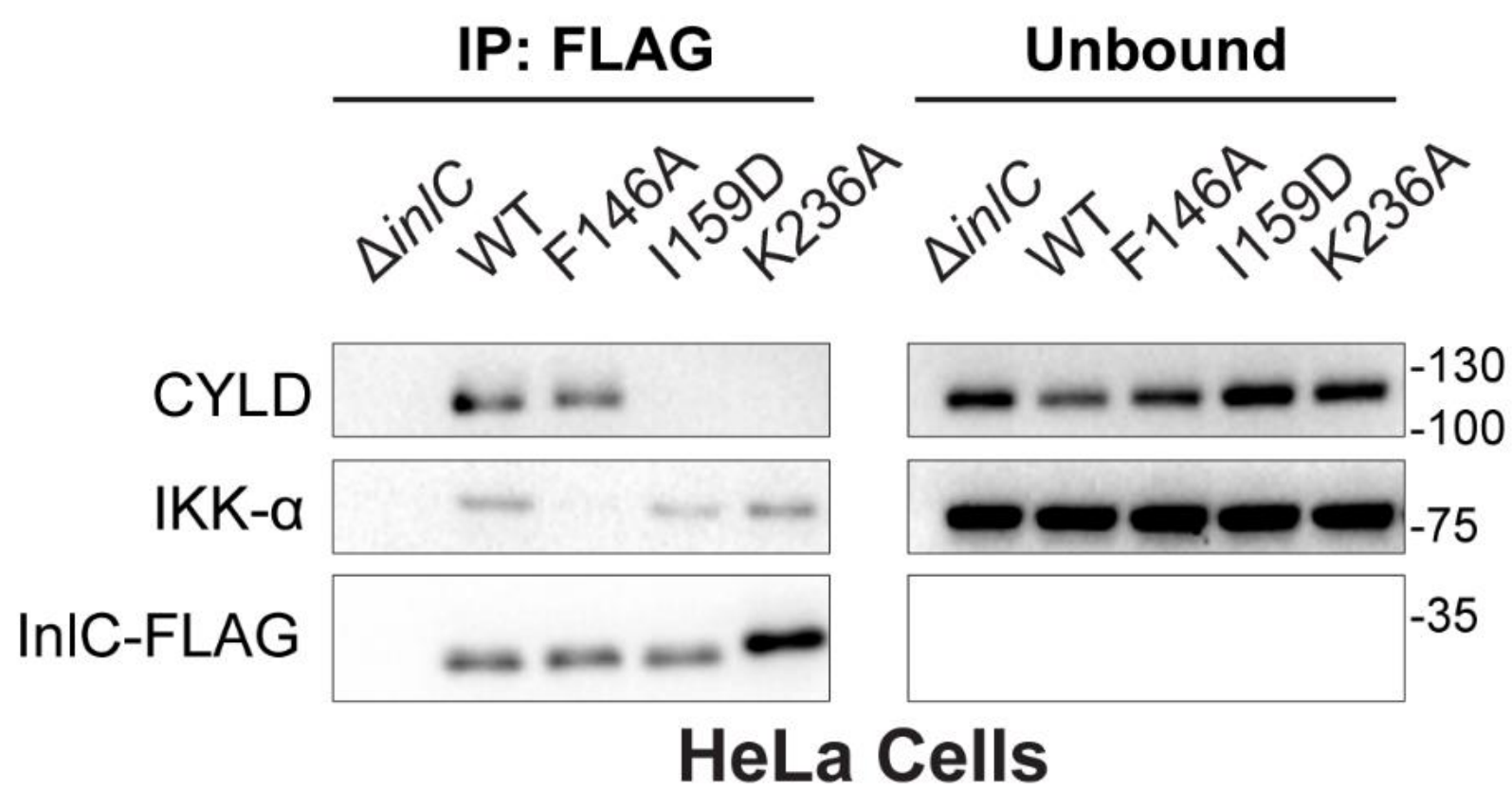

This leads us to speculate that only a fraction of total IKK-α is bound to InlC, however the lack of quantitative measures to support this notion led us to refrain from claiming so in the manuscript. Nonetheless, the western blots related to these observations are included in the accompanying Source Data file.

Reviewer #3:

"This study identifies a strategy by which a bacterial effector manipulates host ubiquitin signaling during infection. The authors show that the secreted Listeria effector InlC interacts with the host deubiquitinase CYLD and that this interaction, together with RNF213-dependent ubiquitination, is required for CYLD recruitment to cytosolic bacteria. They further demonstrate that this axis influences bacterial virulence in vivo. The concept that pathogens counteract ubiquitin-mediated host defense by hijacking deubiquitinating enzymes is important and of broad interest to the field of host–pathogen interactions, and several lines of evidence presented here support this model. However, the principal limitation of the study is the lack of mechanistic resolution connecting these molecular events to a defined intracellular antibacterial pathway and, ultimately, to bacterial growth or survival. While the data establish InlC–CYLD interaction and RNF213 dependence, it remains unclear how CYLD recruitment functionally alters host ubiquitin-dependent defenses (e.g., xenophagy, actin-based motility regulation, or inflammatory signaling) and which of these processes explains the observed virulence phenotype. As a result, the study currently bridges molecular interaction and in vivo outcome without demonstrating the causal intracellular mechanism linking them. Addressing this gap would substantially strengthen the impact and mechanistic completeness of the work."

Response: We thank the reviewer for their supportive and constructive comments.

"Major concerns
1. The authors show that CYLD localization to bacteria requires both InlC–CYLD interaction and RNF213 activity. However, the mechanistic basis of this recruitment remains unclear. CYLD is a K63-specific deubiquitinase and would be expected to recognize ubiquitin chains independently of InlC. It is therefore difficult to understand how binding to InlC enables CYLD to sense or engage ubiquitinated bacteria. The current data support that InlC–CYLD interaction is necessary, but do not explain how this interaction functionally contributes to CYLD positioning relative to the ubiquitin coat."

Response: We appreciate this thought-provoking comment that led to experiments we have now included in a new Results section (see below) describing a new Supplementary Figure 8 (see below). In brief, we found that deletion of an extra-catalytic region of CYLD's C-terminus (a zinc binding B-box feature) impaired CYLD recruitment to ubiquitin-positive bacteria despite InlC-CAP-Gly2 binding. CYLD's B-box feature is known to regulate CYLD dimerization, a key requirement for recruitment to immune receptors via association with the adaptor protein SPATA2 (PMID: 27591049).

Page 7, line 233 from revised manuscript:
The B-box module of CYLD is required for its recruitment to ubiquitin-positive bacteria
The absence of InlC enrichment at CYLD-positive bacteria led us to question how CYLD is recruited to cytosolic *Lm*. We postulated that beyond InlC binding, there may be intrinsic factors of CYLD required for successful delivery and/or retention at the bacterial surface. Within

CYLD's USP domain lies a zinc-binding B-box module (**Supplementary Fig. 8A**) that permits intermolecular interactions and partially regulates CYLD localization[23,30]. In uninfected RAW264.7 macrophages, HA-CYLD retained cytosolic localization upon deletion of this module (**Supplementary 8B**). While deletion of this module did not impair InlC-CYLD binding (**Supplementary Fig. 8C**), we observed impaired recruitment of HA-CYLD$^{\Delta\text{B-box}}$ to ubiquitin-positive Δ*actA* bacteria (**Supplementary Fig. 8D**). A lack of recruitment was also observed with truncated versions of HA-CYLD lacking either its USP or CAP-Gly domains, despite the former retaining InlC-CAP-Gly2 binding (**Supplementary Fig. 8C and 8D**). These findings suggest a co-requirement of InlC-CAP-Gly2 binding and the presence of CYLD's B-box module for InlC-dependent CYLD recruitment to the bacterial surface.

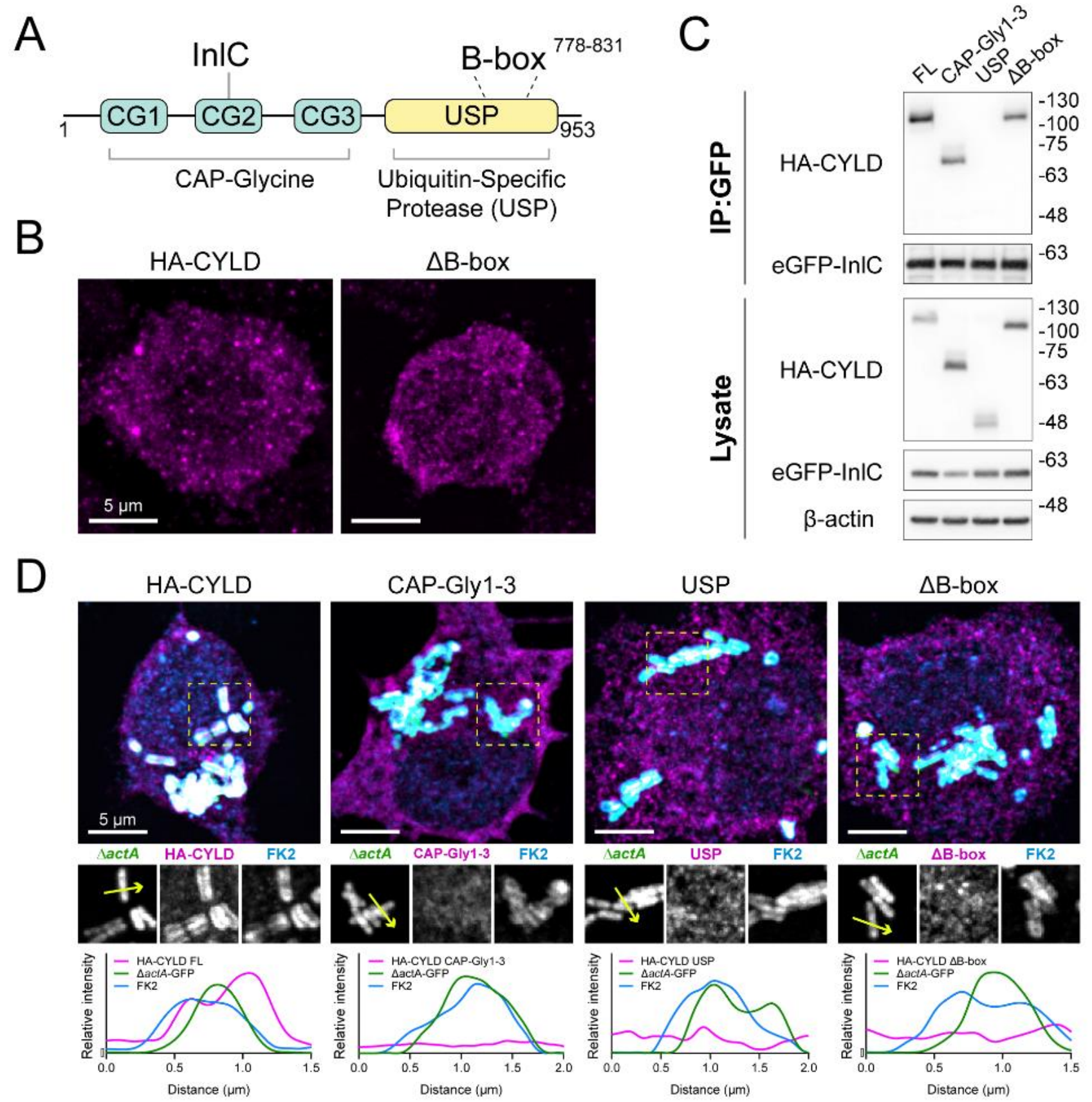

Supplementary Figure 8. The B-box module of CYLD is required for its recruitment to ubiquitin-positive *Lm*. (A) Schematic of HA-CYLD highlighting the InlC binding site (CAP-Gly2) and B-box module (USP). (B) Localization of HA-CYLD and HA-CYLD$^{\Delta\text{B-box}}$ in uninfected, *Cyld* KO RAW264.7 cells. Scale bar, 5 μm. Images are representative of three experimental replicates. (C) Co-immunoprecipitation of eGFP-InlC with full-length or truncated forms of HA-CYLD in *CYLD* KO HEK293T cells. (D) Localization of full-length or truncated forms of HA-CYLD in *Cyld* KO RAW264.7 cells infected with Δ*actA Lm* (4 hours post-infection). Scale bar, 5 μm. Relative intensity profiles correspond to the arrow in the *Lm* channel. Images are representative of three experimental replicates.

Furthermore, we have also updated our Discussion to speculate how InlC-CYLD binding promotes CYLD recruitment. Please see above for our response to Reviewer #2, Point 1.

"2. In addition, the subcellular localization of InlC itself is not clearly addressed. Since the proposed model implies that InlC functions at the bacterial surface to recruit or stabilize CYLD, it would be important to determine:

・1) Whether InlC accumulates in the vicinity of cytosolic bacteria.

・2) Whether such localization depends on CYLD and/or RNF213-mediated ubiquitination.

・3) Whether disruption of CYLD binding alters InlC distribution.

Without these data, the conclusion that InlC directly recruits CYLD to bacteria appears mechanistically overstated. Clarifying the spatial relationship among InlC, CYLD, and the ubiquitin coat would significantly strengthen the proposed model."

Response: Please see our response to Reviewer #2 (Page 7,8 above) regarding the 1st and 3rd points regarding InlC localization. In brief, InlC does not accumulate on the surface of cytosolic bacteria and we found disrupting InlC-CYLD binding did not alter InlC's diffuse, cytosolic localization. As noted in our response to Reviewer #2, we have included these findings as Supplementary Figure 7 and modified our Discussion.

In response to the 2nd comment as to whether CYLD or RNF213-mediated ubiquitination impacts InlC distribution, we assessed InlC localization in wildtype, *Cyld*$^{-/-}$, or *Rnf213*$^{-/-}$ knockout RAW264.7 cells. We observed similar diffuse, cytosolic localization of InlC across all three cell lines using both motile and non-motile bacteria (see below). As these indifferent findings are redundant with Supplementary Figure 7A, we have opted to omit this figure from the manuscript.

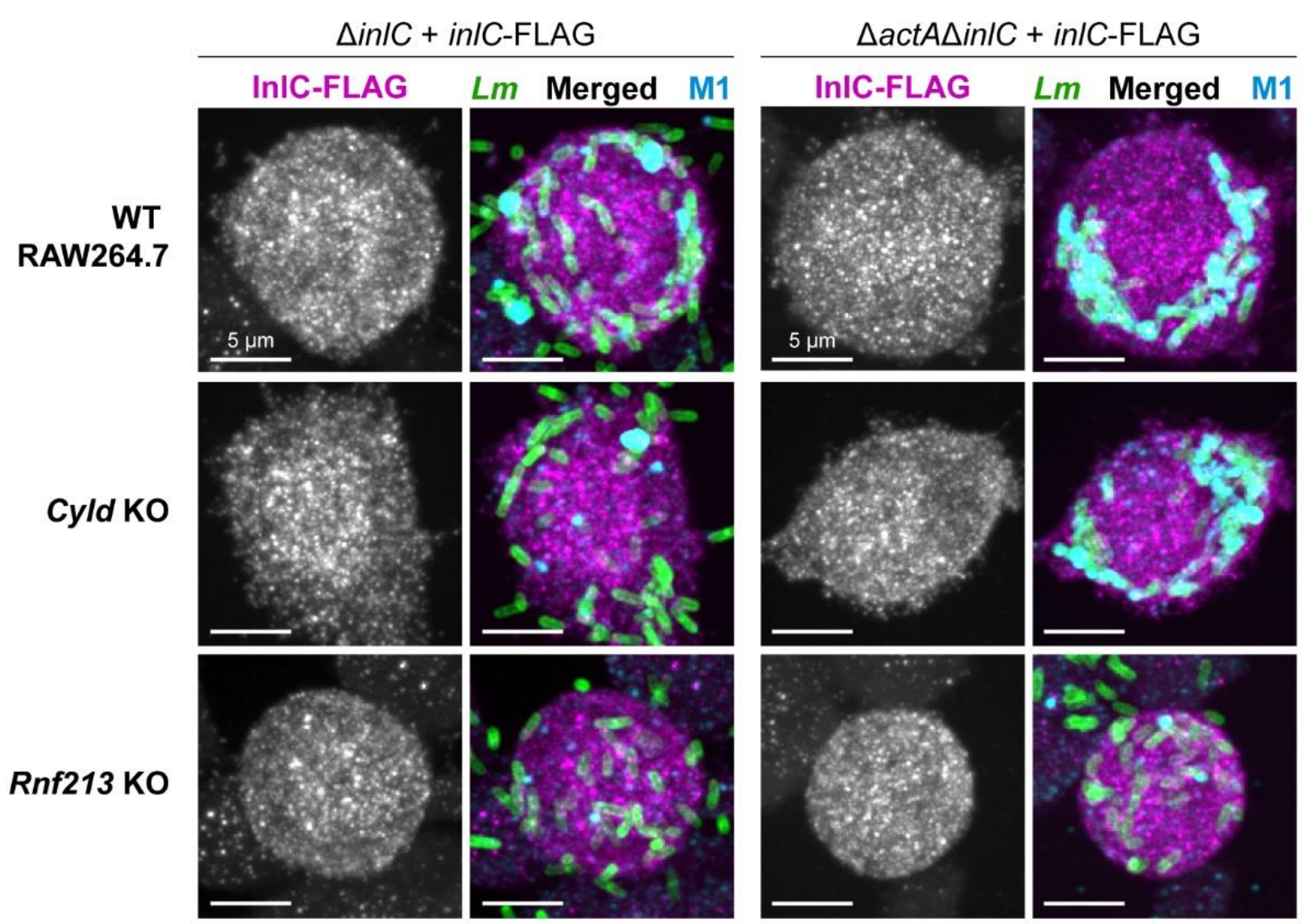

"3. The manuscript shows that InlC recruits CYLD to remove RNF213-dependent ubiquitin from cytosolic Lm. However, it remains unclear which host defense pathway is functionally counteracted by this deubiquitination. The in vivo virulence phenotype is presented without linking it to a defined intracellular process. Is the primary effect on xenophagy, actin-based motility/cell-to-cell spread, or inflammatory signaling?"

Response: We did not observe a role for InlC on bacterial growth in macrophages *in vitro*, consistent with prior observations (PMID: 8878044). We also did not observe an impact upon loss of InlC on *Lm* cell-to-cell spread in both RAW264.7 macrophages and MEFs, two cell types in which we observed InlC-dependent CYLD recruitment. As RNF213 is an interferon-stimulated gene (PMID: 37655297), these assays were also performed in IFN-γ-primed cells, but again no differences were observed. Nonetheless, we value the importance of these results and have incorporated them into our manuscript as a new Supplementary Figure 12 (see below).

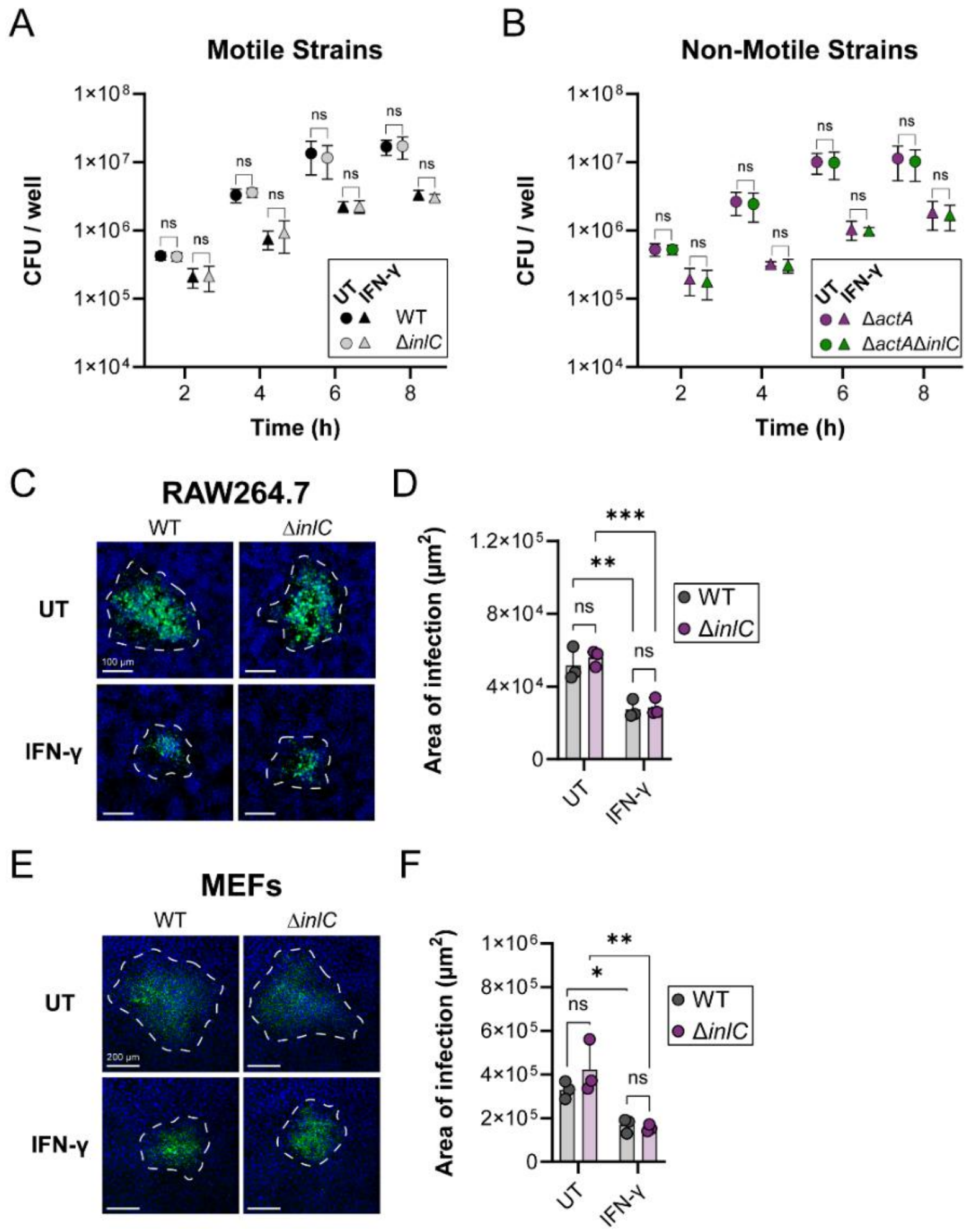

Supplementary Figure 12. Loss of InlC does not impact bacterial growth in macrophages, or *Lm* cell-to-cell spread in RAW264.7 cells and MEFs. (A-B) Bacterial growth of motile (A) or non-motile (B) *Lm* strains in untreated or IFN-γ-primed RAW264.7 cells (2-8 hours post-infection). All colony forming units (CFU) are displayed as mean ± SD enumerated across three experimental replicates. *P* values were determined using a two-way ANOVA (ns = not significant). (C-D) *Lm* cell-to-cell spread in untreated or IFN-γ-primed RAW264.7 cells (20 hours post-infection). Dotted lines delineate the edge of the infection area. Scale bar, 100 µm. Images are

representative of three experimental replicates. (D) Quantification of the area of infection (μm) was assessed from (30) foci per experimental replicate and displayed as mean ± SD across three replicates. *P* values were determined using a two-way ANOVA with Tukey's post-hoc test (ns = not significant). (E-F) *Lm* cell-to-cell spread in untreated or IFN-γ-primed MEFs (20 hours post-infection). Dotted lines delineate the edge of the infection area. Scale bar, 200 μm. Images are representative of three experimental replicates. (D) Quantification of the area of infection (μm) was assessed from (15-26) foci per experimental replicate and displayed as mean ± SD across three replicates. *P* values were determined using a two-way ANOVA with Tukey's post-hoc test (ns = not significant).

To explore the potential impact of InlC-CYLD binding on inflammatory signaling, we assessed the TNF-α-induced activation of NF-κB signaling based on a well-established assay in *Lm*-infected HeLa cells (PMID: 20855622, 23403554). During *Lm* infection, we observed comparable levels of InlC-dependent inhibition of p65 nuclear translocation that does not specifically require InlC-CYLD binding (see below):

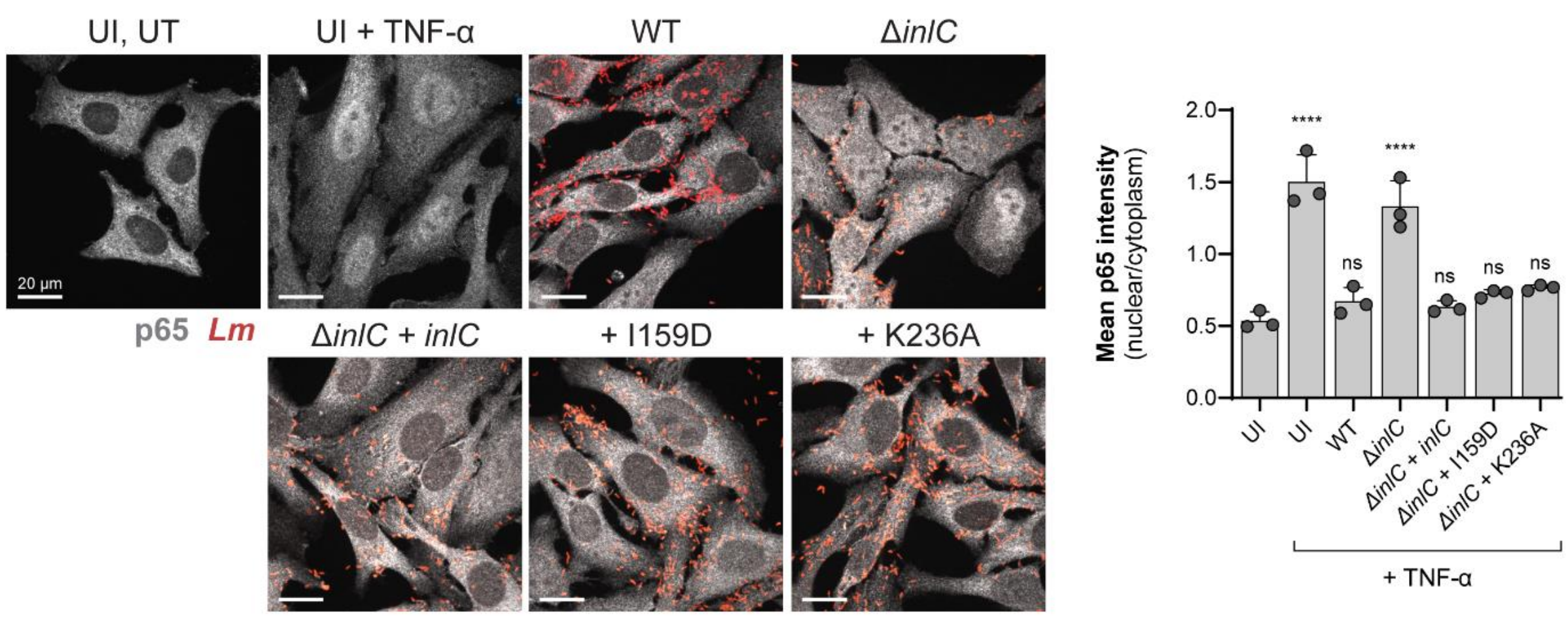

Our attempts to repeat this assay in a more relevant model, such as macrophages, were unsuccessful due to biological differences including higher levels of inherent p65 translocation during infection. For this reason, we opted to omit these results from the manuscript as this evidence alone feels insufficient to exclude a role for InlC-CYLD binding in altering inflammatory signaling.

"4. Although InlC–CYLD interaction is demonstrated by co-IP, CYLD recruitment to bacteria requires RNF213-dependent ubiquitination. This suggests that ubiquitin serves as a primary scaffold for CYLD localization, while InlC may function in stabilizing or positioning CYLD rather than directly recruiting it. The authors should clarify this hierarchical recruitment model."

Response: We thank the others for raising this concern. To clarify this hierarchical recruitment model, we performed a time course experiment using Δ*actA Lm*. As depicted in the new Supplementary Figure 4, we observed that InlC accumulation in host cells coincides with the emergence of HA-CYLD-positive bacteria at 3-4 hours post-infection, whereas at earlier timepoints ubiquitin-positive *Lm* appear void of CYLD. Furthermore, we clarify that all CYLD-positive bacteria are ubiquitin-positive in both WT and Δ*actA* bacteria.

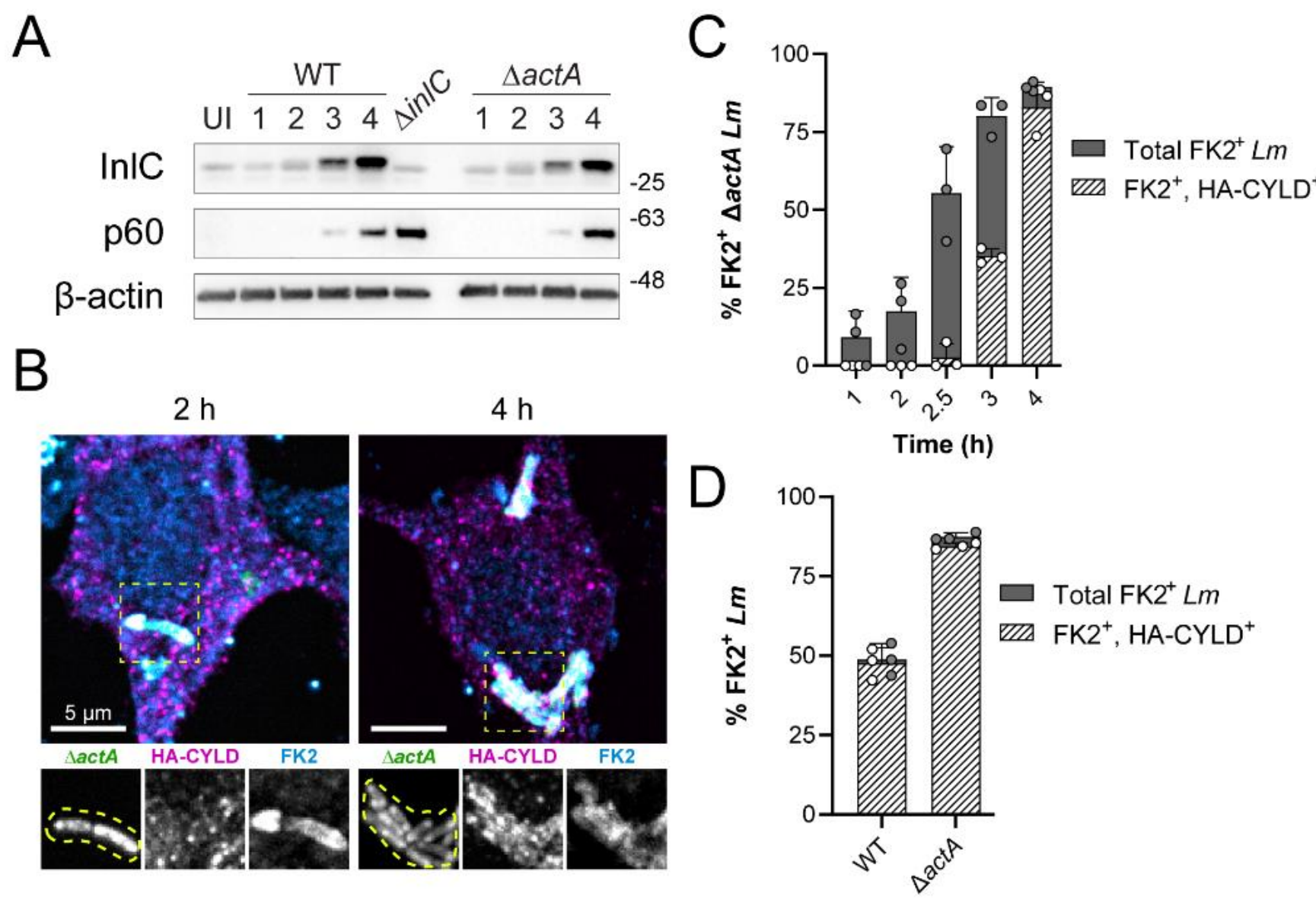

Supplementary Figure 4. InlC secretion coincides with CYLD recruitment to ubiquitin-positive bacteria.
(A) Immunoblot of InlC secretion during *Lm* infection of RAW264.7 cells. Detection of *Lm* p60 confirms infection efficacy. (B-C) Time course of HA-CYLD recruitment to ubiquitin (FK2)-positive Δ*actA* bacteria during infection of RAW264.7 cells. Representative images of ubiquitin-positive *Lm* at 2- and 4-hours post-infection. Dashed lines highlight clusters of non-motile bacteria within the region of interest. Scale bar, 5 µm. Images are representative of three experimental replicates. (C) Quantification of total FK2-positive and FK2-, CYLD-positive bacteria. Mean ± SD is displayed based on quantification of (15-20) RAW264.7 cells per experimental replicate. (D) HA-CYLD localization to ubiquitin (FK2)-positive WT and Δ*actA* bacteria during infection of RAW264.7 cells (4 hours post-infection). Quantification of total FK2-positive and FK2-, CYLD-positive bacteria. Mean ± SD is displayed based on quantification of (15-16) RAW264.7 cells per experimental replicate.

We have also updated our model (new Supplementary Figure 13, see below) to depict this hierarchical recruitment:

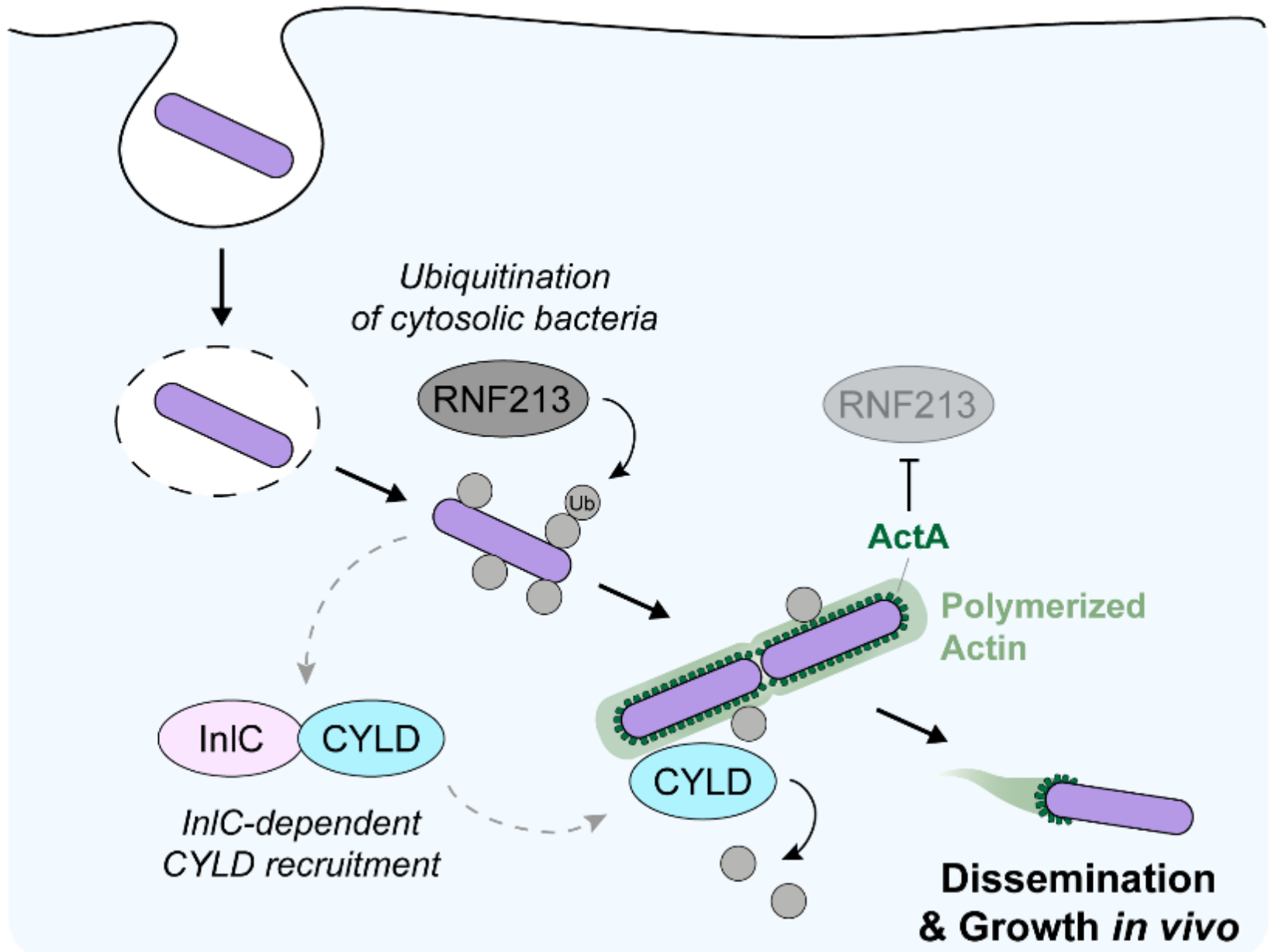

"5. The study relies on RNF213 knockout to infer that ubiquitination is required for CYLD recruitment. However, RNF213 may have scaffolding functions independent of its ligase activity. The authors should test an E3-deficient RNF213 mutant (e.g., RING domain mutant) to determine whether CYLD localization depends specifically on RNF213 catalytic activity rather than its mere presence."

Response: Technical limitations prevented us from further studying the role of RNF213 at the bacterial surface including the lack of commercially available resources, challenges in cloning epitope-tagged RNF213 (~5,200 aa.), and limitations in overexpressing a large plasmid in our transfection-based system where the efficiency is already quite low (1-3%).

Nonetheless, we value the depth of this comment and have updated the Discussion (see below) with this possibility. In addition, we cite a recent study that corroborates RNF213 E3 ligase activity as the determinant for InlC-dependent CYLD recruitment (PMID: 41757053), alongside evidence that supports potential interplay between RNF213 and CYLD at the bacterial surface (PMID: 39506105, 35780935).

Page 9, Line 352 of the revised manuscript
Here, we identify RNF213 as a critical host determinant for the ubiquitination of *Lm* in macrophages. While RNF213 possesses a RING domain, coating of *Lm* has been demonstrated to occur non-canonically through an RZ finger domain[2]. In the case of gram-negative bacteria, RNF213-mediated ubiquitination leads to the recruitment of additional E3 ligases such as the linear ubiquitin chain assembly complex (LUBAC)[3]. Recent evidence suggests that ubiquitination by RNF213 is the predominant cue for CYLD recruitment to *Lm* rather than ensuing E3 ligases such as LUBAC[36]. The ability of RNF213 to interact with, and even

ubiquitinate CYLD raises the possibility of interplay between these two proteins at the bacterial surface[37,38]. Whether RNF213 has scaffolding functions at the bacterial surface independent of its E3 ligase activity remains an area of future exploration.

Minor comments

1. The manuscript alternates between "gram-positive" and "Gram-positive." Please standardize capitalization throughout.

Response: We have corrected this in the manuscript.

2. Both spellings "Ubiquitination" vs "ubiquitylation" are used. Please adopt a single convention according to journal style.

Response: We have corrected this in the manuscript.

3. Methods wording

•"scarified" should be "sacrificed"

Response: We have corrected this in the manuscript.

•"bacterial/mL" should read "bacteria/mL"

Response: We have corrected this in the manuscript.

•"Clonetech" should be "Clontech"

Response: We have corrected this in the manuscript.

•"Tween 20" vs "Tween-20" should be unified

Response: We have corrected this in the manuscript.

Response to Reviewer's comments

Reviewer #2:

"The authors have addressed the core technical concerns raised in the initial review. While some mechanistic gaps remain regarding the spatial recruitment model, the newly added experimental data have strengthened the manuscript.

The new immunofluorescence data showed that InlC does not accumulate at the bacterial surface alongside CYLD. Therefore, the exact mechanism underlying this recruitment remains undefined. The authors' choice to tone down their language, shifting from descriptive terms like "targeting" to strictly defining it as a biochemical "protein-protein interaction," is a necessary and responsible correction. While the proposed displacement or transient "hit-and-run" models in the Discussion are speculative, they have properly recognized this structural limitation.

The endogenous CYLD staining experiment is a useful addition, especially with the Cyld KO control. However, the signal appears relatively weak, and the manuscript should clearly acknowledge that most localization data still rely on HA-CYLD expression.

Overall, I am satisfied with the authors' responses and would support publication of this work upon minor revisions to avoid overstatement of a direct targeting/recruitment mechanism and to clearly state current limitations of the imaging data."

<u>Response:</u> We thank this reviewer for their constructive feedback. In the Results and Discussion section, we have further adjusted our descriptive language to avoid overstating a direct targeting/recruitment mechanism:

Removed sentence from page 9, line 316 from discussion of revised manuscript:
"To our knowledge, InlC is the first known bacterial virulence factor that targets CYLD."

Modified sentence from page 9, line 326 from discussion of revised manuscript:
"To counter this, InlC binds CYLD to indirectly promote deubiquitinase recruitment to the bacterial surface."

Modified the legend of our model, now included as Figure 5 (formerly Supp. Fig. 13):
"Figure 5. Model depicting the InlC-dependent recruitment of CYLD to the *Lm* surface to promote systemic disease."

Furthermore, in the discussion we now clearly state a major limitation of our imaging data is the use of overexpression

Page 9, line 343 from revised manuscript:
"A major limitation in our ability to assess this displacement model arises from the use of CYLD overexpression. Live-cell imaging of endogenously tagged CYLD may elucidate whether InlC promotes the recruitment of distinct populations of CYLD."

Reviewer #3:

"I thank the authors for their careful and constructive revisions. The revised manuscript includes several new experiments and textual changes that substantially address my previous concerns, particularly regarding InlC localization, CYLD recruitment, and the hierarchical relationship between bacterial ubiquitination and CYLD association.

Although some mechanistic details remain unresolved, including the precise downstream antibacterial pathway and the specific requirement for RNF213 catalytic activity, the authors have reasonably acknowledged these limitations. I do not think these remaining issues compromise the main conclusions of the study.|

I am satisfied with the revision and support acceptance of the manuscript."

<u>Response</u>: We thank Reviewer 3 for their support of our manuscript.
